# Design, synthesis and evaluation of novel 2-oxoindoline-based acetohydrazides as antitumor agents

**DOI:** 10.1038/s41598-022-06887-0

**Published:** 2022-02-21

**Authors:** Do T. M. Dung, Eun J. Park, Duong T. Anh, Dung T. P. Phan, Ik H. Na, Joo H. Kwon, Jong S. Kang, Truong T. Tung, Sang-Bae Han, Nguyen-Hai Nam

**Affiliations:** 1 Hanoi University of Pharmacy, 13-15 Le Thanh Tong, Hanoi, Vietnam; 2 College of Pharmacy, Chungbuk National University, 194-31, Osongsaengmyung-1, Heungdeok, Cheongju, Chungbuk 28160 Republic of Korea; 3 Korea Research Institute of Bioscience and Biotechnology, Cheongju, Chungbuk Republic of Korea; 4 Faculty of Pharmacy, PHENIKAA University, Hanoi, 12116 Vietnam; 5 PHENIKAA Institute for Advanced Study (PIAS), PHENIKAA University, Hanoi, 12116 Vietnam

**Keywords:** Chemical libraries, Drug discovery and development, Lead optimization, Screening, Structure-based drug design

## Abstract

In our search for novel small molecules activating procaspase-3, we have designed and synthesized two series of novel (*E*)-*N'*-arylidene-2-(2-oxoindolin-1-yl)acetohydrazides (**4)** and *(Z)-*2-(5-substituted-2-oxoindolin-1-yl)-*N'*-(2-oxoindolin-3-ylidene)acetohydrazides (**5)**. Cytotoxic evaluation revealed that the compounds showed notable cytotoxicity toward three human cancer cell lines: colon cancer SW620, prostate cancer PC-3, and lung cancer NCI-H23. Especially, six compounds, including **4f–h** and **4n–p**, exhibited cytotoxicity equal or superior to positive control PAC-1, the first procaspase-3 activating compound. The most potent compound **4o** was three- to five-fold more cytotoxic than PAC-1 in three cancer cell lines tested. Analysis of compounds effects on cell cycle and apoptosis demonstrated that the representative compounds **4f, 4h, 4n, 4o** and **4p** (especially **4o**) accumulated U937 cells in S phase and substantially induced late cellular apoptosis. The results show that compound **4o** would serve as a template for further design and development of novel anticancer agents.

## Introduction

Normal cells in human body divide and die in a tightly regulated manner. Cell cycle and apoptosis are two processes linked to normal cellular growth and death. In case abnormality occurs, the cells keep dividing and are able to escape apoptosis, leading to the formation of extra mass tissue in the body, known as tumors. Malignant tumors, or cancer, remains one of the deadliest diseases nowadays since the cancer cells are able to spread throughout the body and metastasize to other organs, making the treatment extremely difficult^1^. Over decades, targeting cell cycle and apoptosis, especially apoptosis or programmed cell death process, are among the most common and effective approaches for anticancer drug development^2,3^.

With advances in molecular cell biology, many proteins involved in cellular apoptotic pathways, e.g. BIM, BAX, Bcl-2, p53, RIP, DED, Apo2L, and XIAP, to name a few, have been identified and employed as molecular targets for anticancer therapy^3^. As a result, a number of small molecules targeting these proteins have been discovered. For example, GDC-0152 (a XIAP’s inhibitor), tenovin-1 (a p53 activator), or ABT-199 (an inhibitor of Bcl-2) have been demonstrated to effectively induce apoptosis and ultimately caused the death of cancer cells^4–6^.

**Figure 1:**
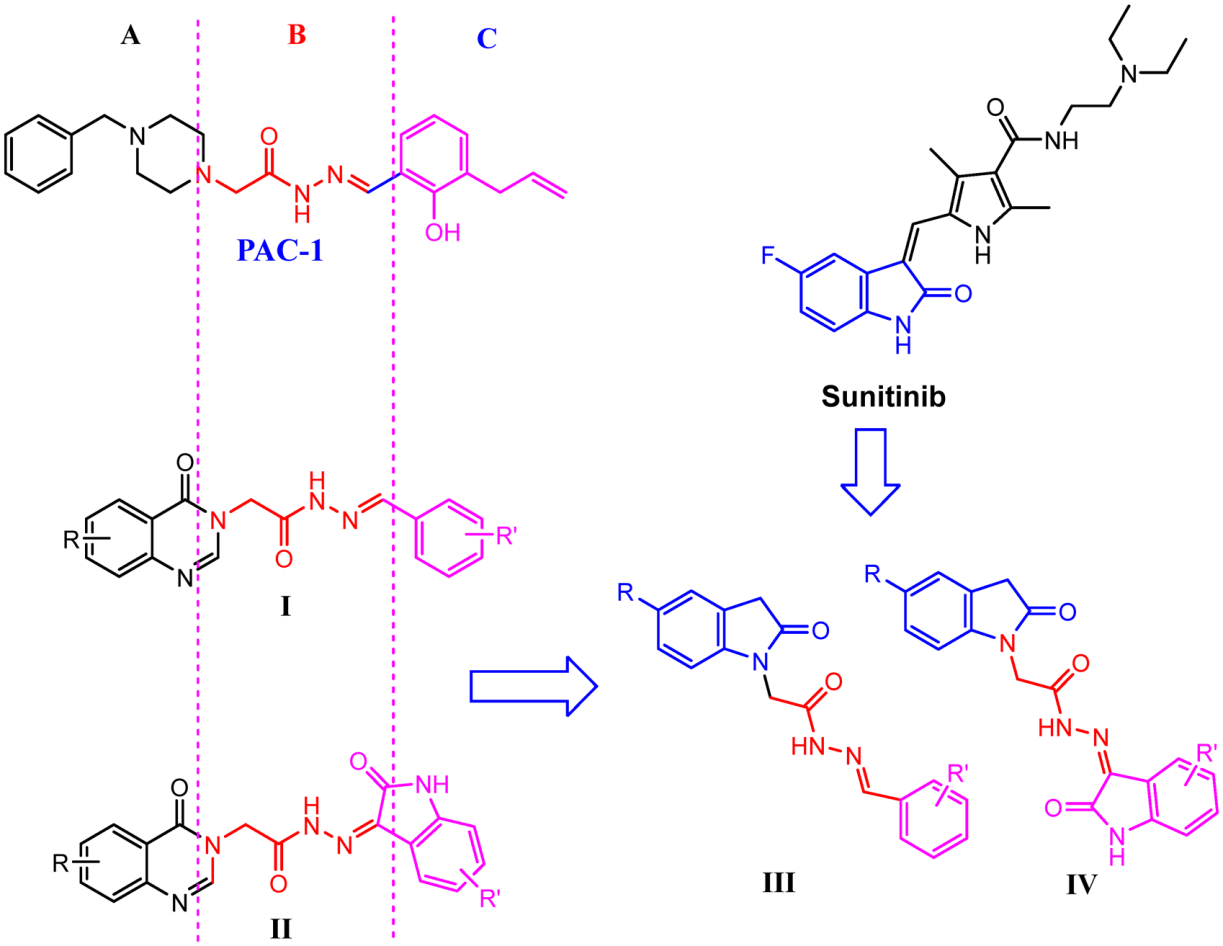
Structure of PAC-1, 4-oxoquinazoline-acetohydrazides (**I**, **II**) and design of novel 2-oxoindoline-based acetohydrazides (**III, IV**).

Also played important roles in regulation of apoptotic pathways are caspases^7,8^. Currently, caspases, with at least fourteen members, are a large group of of cysteine proteases enzymes^7,8^. These enzymes are involved in both extrinsic and intrinsic pathways of the apoptotic machine^7,8^. Among these, caspase-3, known as the executioner caspase, is one of the key enzymes regulating apoptosis responses^7,8^. Caspase-3 exists as a low activity zymogen in cells, known as procaspase-3^7,8^, which has been found to be overexpressed in many types of human cancers^9^ (e.g. neuroblastoma^10^, breast cancer^11^, lung carcinoma^12^, hepatocellular carcinoma^13^, lymphoma and Hodgkin's Disease^14^). Due to their overexpression in cancer cells, it is well established that targeting caspases would be more advantageous over inhibiting other apoptotic proteins^9^. Great efforts of medicinal chemists have therefore placed on the development of novel caspase activators. Consequently, several small molecules as caspase activators have been reported^15–18^. In 2016, PAC-1, the first procaspase activating compound (Fig. 1), was granted as Orphan Drug designation for treatment of blioblastoma by the U.S. FDA. Analysis of structure–activity relationships of PAC-1 clearly indicates the importance of the ortho-hydroxy-*N*-acylhydrazone moiety (B-region, Fig. 1) in the interaction with zinc ion in the active binding site of caspases to form a strong complex structure^17,18^. Based on that feature, we recently reported several series of 4-oxoquinazoline-based acetohydrazides (**I**, **II**) which incorporated the *N*-acylhydrazone functionality and found many compounds with potent procaspase-3 activating activity as well as strong antitumor cytotoxicity^19,20^. Encouraged by these results, in this investigation we expand our design to compounds series **III** and **IV** bearing 2-oxoindoline ring. The 2-oxoindoline is an important scaffold with diverse biological potentials^21^. This paper describes the results obtained from synthesis, bioevaluation of these novel compounds.

## Materials and methods

### Chemistry

The reagents, solvents used in this work were purchased from commercially available vendors (mainly, Aldrich, Fluka Chemical Corp. (Milwaukee, WI, USA), or Merck) and used directly unless otherwise indicated. Thin layer chromatography (TLC) was performed in Whatman Silica Gel GF_250_. The TLC plate was visualized using 254 nm UV light. Gallenkamp (LabMerchant, UK) melting Point Apparatus was used for recording melting points of the compounds and are uncorrected. Re-crystallization in solvents or column chromatography on silica gel was used for purification of final compounds. Merck (silica gel 240 to 400 mesh) was used as stationary phase in column flash chromatography. ^1^H NMR were analyzed on a 500 MHz spectrometer (Bruker). DMSO-*d*_6_ was used as NMR solvent unless otherwise indicated. Chemical shifts are reported ppm. Mass spectra of the compounds were performed in PE Biosystems API2000 (electron ionization (EI), Perkin Elmer-USA) and Mariner (Electrospray ionization (ESI), Azco Biotech-USA) mass spectrometers, respectively. The elemental analyses (C, H, N) of the final compounds were recored on a Perkin Elmer elemental analyzer (model 2400).

### Cytotoxicity assay

Three human cancer cell lines: colon cancer (SW620), prostate cancer (PC3), and lung cancer (NCI-H23) were used for sceening the cytotoxicity of the compounds. The cancer cells were purchased from American Type Culture Collection (Manassas, VA, USA). Other reagents/media for cell culture were obtained from GIBCO (Grand Island, New York, USA). The testing cancer cells were culture in Dulbecco’s Modified Eagle Medium until confluence. Then, they were trypsinized and suspended at the level of 3 × 10^4^ cells/mL of cell culture medium. On day 0, cancer cells were seeded at a volume of 180 µL/well of 96-well plates and incubated for 24 h at 37 °C in a 5% CO_2_ incubator. On day 1, 20 µL of various concentrations of testing compounds were added to each well of the 96-well plates. Chemicals were dissolved in dimethyl sulfoxide (DMSO, stock) and diluted in culture medium (1% DMSO) before adding to the culture. After 48 h incubation, the sulforhodamine B assay was used for cell density determination with slight modifications^22–26^. The Probits were used for the calculation of IC_50_ values. The reported IC_50_ were the averages of three independent screening (SD ≤ 10%)^27^.

### Cell cycle analysis

U937 human lymphoma cells (5 × 10^5^/mL per well) were plated in 6-well culture plates and allowed to grow for 24 h. In our first experiment, we examined the effects of 4f, 4h, 4n, 4o, 4p, and PAC-1 on cell cycles at 50 µM. In the second experiment, we examined the dose-dependent effect of 4o at 5, 10, and 30 µM and PAC-1 at 30 µM on cell cycles. The cells were treated with compounds for 24 h, and then harvested. The harvested cells were washed twice with ice-cold PBS, fixed in 75% ice-cold ethanol, and stained with propidium iodide (PI) in the presence of RNase at room temperature for 30 min. The stained cells were analyzed for DNA content using a FACScalibur flow cytometer (BD Biosciences, San Jose, CA, USA) and the data were processed using Cell Quest Pro software (BD Biosciences).

### Apoptosis assay

The Annexin V-FITC/PI dual staining assay was used to determine the percentage of apoptotic cells. U937 cells (5 × 10^5^/mL per well) were plated in 6-well culture plates and allowed to grow for 24 h. In our first experiment, we examined the effects of 4f, 4h, 4n, 4o, 4p, and PAC-1 on apoptosis at 50 µM. In the second experiment, we examined the dose-dependent effect of 4o at 5, 10, and 30 µM and PAC-1 at 30 µM on apoptosis. The cells were treated with compounds for 24 h, and then harvested. The harvested cells were washed twice with ice-cold PBS and incubated in the dark at room temperature in 100 mL of 1 × binding buffer containing 1 µL Annexin V-FITC and 12.5 mL PI. After 15 min incubation, cells were analyzed for percentage undergoing apoptosis using a FACScalibur flow cytometer (BD Biosciences). The data were processed using Cell Quest Pro software (BD Biosciences).

### Caspase-3 activation assay

Caspase activity was measured by using caspase 3 assay kit according to the manufacturer’s instructions (abcam, MA, USA). U937 human lymphoma cells (5 × 10^5^/mL per well) were plated in 6-well culture plates and allowed to grow for 24 h. The cells were treated with compounds for 24 h, and then harvested. The harvested cells were washed twice with ice-cold PBS and treated with lysis buffer included in the kit. Cell lysate (100 µg/50 µL) was mixed with 50 µL of 2 × reaction buffer and 5 µL of DEVD-*p*-NA substrate as the instruction of caspase-3 assay kit (Abcam, cat. N. ab39401). Fluorescence was measured after one-hour incubation.

## Results and discussion

### Chemistry

**Figure 2:**
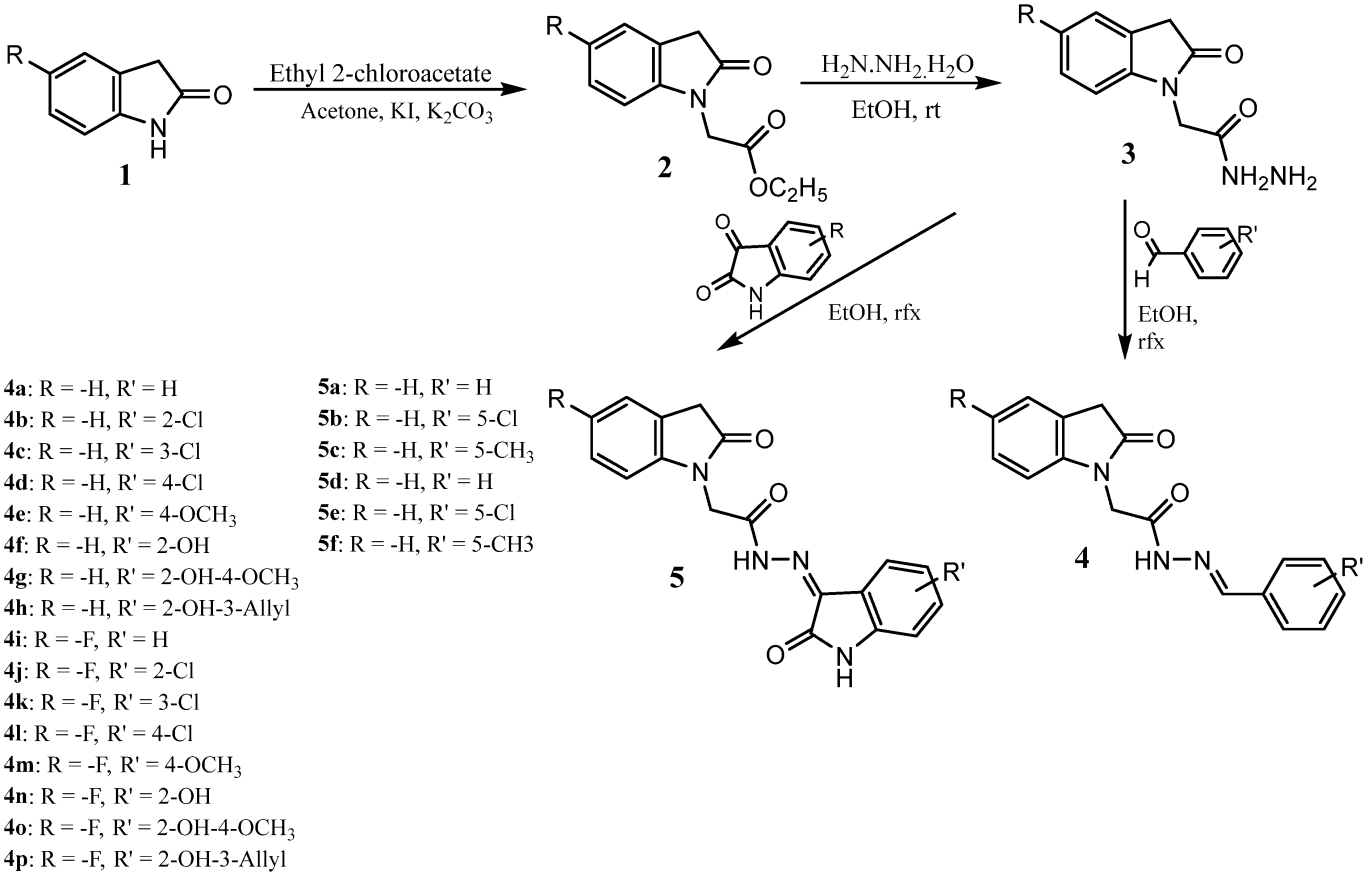
Synthesis of novel 2-oxoindoline-based acetohydrazides (**4, 5**).

Figure 2 illustrates the synthesis of the target 2-oxoindoline-based acetohydrazides (**4**, **5**). The synthesis of final compounds proceeded via three steps. The first step was a nucleophilic substitution between 2-oxoindoline derivatives and ethyl chloroacetate with the presence of potassium carbonate with a catalytic amount of KI in acetone to afford the selectively *N-*alkylated intermediate esters (**2**). The yields of this step were generally excellent (90–93%). The second step was acyl transfer reaction of the esters **2a–b** with hydrazine monohydrate afforded the hydrazides **3a–b**. This reaction occurred under refluxing conditions, and ethanol was found as an effective solvent for this reaction. With hydrazids **3a–b** in hand**,** the desired products **4a–p**, **5a–f** were obtanined in moderate overall yields via aldol condensation of **3a–b** with benzaldehydes or isatins (Fig. 2).

The identification of the structures of the **4**, **5** were performed using the analysis of IR, MS, ^1^H NMR and ^13^C NMR. The most important peak was the singlet at around 4.4–5.3 ppm of ^1^H NMR spectra which attributable for two protons occurred of the methylene protons of *N*-alkylated compounds. Please see Supporting Information for copy of ^1^H NMR and ^13^C NMR spectra. The configuration of the compound was well established previously^28–31^.

The preparation of the acetohydrazides incorporating 2-oxoindoline (**4**, **5**) was described as follow: K_2_CO_3_ (206.9 mg, 1.5 mmol) were added to a solution of 5-substituted-2-oxoindoline (1 mmol) in 50 mL acetone. The mixtures were then refluxed for 30 min. A catalytic amount of KI (16.6 mg, 0.1 mmol) was then added to the mixture. The whole mixture was stirred for additional 15 min followed by dropwise of 0.13 mL of ethyl chloroacetate (1.2 mmol). Finally, the mixture was heated to 60 °C for additional of 3 h. After reaction completion showed by TLC, the reaction solvents were evaporated. The residues were then re-dissolved in DCM (50 mL), filtered and evaporatred the solvent under reduced pressure to afford the intermediate ester derivatives **2**. The compounds were used for the next step without additional purification.

Each of the intermediate esters **2** (0.5 mmol) was dissolved in ethanol (10 mL). Then, hydrazin hydrate 80% (0.12 mL) was added dropwise. The reaction mixture was stirred at 25 °C until all starting material consumed. The obtained white precipitates solid were filtered, washed with 3 × 20 mL of cold-ethanol. The solid turned yellow as pure **3a–b** were dried and used to the next step without additional purification.

Absolute ethanol (20 mL) was added to dissolve the acetohydrazides **3a–b** (0.5 mmol), followed by 2 drops of glacial acetic acid, benzaldehyde or isatine derivatives (1.0 mmol) were added. The mixtures were refluxed until the reaction completed by TLC (4–6 h). The resulting precipitate was filtered and washed with ethanol (3 times). The obtained yellow solid residuces were dried under reduced pressure. The residuces were purified using either re-crystalised in ethanol, or column chromatography (MeOH:DCM) to obtain the desired product **4a–p**, **5a**–**f**.

#### (E)-N'-Benzylidene-2-(2-oxoindolin-1-yl)acetohydrazide (4a)

Yellow solid; Yield: 55%. mp: 167–168 °C. *R*_*f*_ = 0.51 (DCM:MeOH = 14:1). *IR (KBr, cm*^*−1*^*):* 3186 (NH); 3059 (CH aren); 2968, 2851 (CH, CH_2_); 1721, 1678 (C=O); 1614 (C=C). ^1^H-NMR (500 MHz, DMSO-*d*_*6*_): δ 11.72, 11.70 tautomeric NH (keto-enol), exchangeable by D_2_O (s, 1H, CONH); 8.25, 8.06 (s, 1H, CH=N); 7.75, 7.71 (dd, *J* = 7.75 Hz, *J*′ = 1.75 Hz, 2H, H-2′, H-6′); 7.48–7.44 (m, 3H, H-3′, H-4′, H-5′); 7.29 (d, *J* = 7.00 Hz, 1H, H-7); 7.23 (t, *J* = 7.50 Hz, 1H, H-5); 7.02 (t, *J* = 7.50 Hz, 1H, H-6); 6.95 (d, *J* = 8.00 Hz, 1H, H-4); 4.89, 4.47 (s, 1.50H, 0.50H, CH_2_-CONH); 3.64 (s, 2H, H-3a, H-3b). ^13^C-NMR (125 MHz, DMSO-*d*_*6*_): δ 175.20, 168.50, 163.78, 147.76, 145.18, 144.49, 134.54, 134.47, 130.63, 130.74, 129.30, 127.91, 127.59, 127.43, 125.02, 124.90, 124.73, 124.64, 122.41, 122.23, 109.23, 109.12, 41.29, 35.49. HR-MS (ESI) *m/z*: 294.1225 [M + H]^+^. HR-MS (ESI) *m/z* calculated for C_17_H_16_N_3_O_2_ [M + H]^+^ 294.1243. Found 294.1225.

#### (E)-N'-(2-Chlorobenzylidene)-2-(2-oxoindolin-1-yl)acetohydrazide (*4b*)

Yellow solid; Yield: 60%. mp: 179–181 °C. *R*_*f*_ = 0.53 (DCM:MeOH = 14:1). *IR (KBr, cm*^*−1*^*):* 3179 (NH); 3069 (aromatic CH, & CH2 aliphatic); 2984, 2945 (CH, CH_2_); 1717, 1680 (C=O); 1613 (C=C). ^1^H-NMR (500 MHz, DMSO-*d*_*6*_): δ 11.95, 11.85 (s, 1H, CONH); 8.64, 8.44 (s, 1H, CH=N); 8.07, 7.95 (dd, *J* = 7.50 Hz, *J*′ = 2.00 Hz, 1H, H-6′); 7.56–7.42 (m, 3H, H-3′, H-4′, H-5′); 7.30 (d, *J* = 7.00 Hz, 1H, H-7); 7.23 (t, *J* = 7.25 Hz, 1H, H-5); 7.02 (t, *J* = 7.75 Hz, 1H, H-6); 6.96 (d, *J* = 8.00 Hz, 1H, H-4); 4.91, 4.48 (s, 2H, CH_2_–CONH); 3.64 (s, 2H, H-3a, H-3b). ^13^C-NMR (125 MHz, DMSO-*d*_*6*_): δ 175.20, 175.12, 168.66, 163.00, 145.12, 144.85, 143.65, 140.53, 133.65, 133.47, 132.09, 131.89, 131.74, 130.39, 128.14, 128.09, 127.95, 127.91, 127.53, 127.38, 125.02, 124.90, 124.76, 124.65, 122.44, 122.25, 109.24, 109.12, 42.08, 41.33, 40.61, 35.49. HR-MS (ESI) *m/z*: 328.0846 {[M + H]^+^, ^35^Cl}; 330.0815 {[M + H]^+^, ^37^Cl}. HR-MS (ESI) *m/z* calculated for C_17_H_15_ClN_3_O_2_ [M + H]^+^ 328.0853. Found 328.0846.

#### (E)-N'-(3-Chlorobenzylidene)-2-(2-oxoindolin-1-yl)acetohydrazide (*4c*)

Yellow solid; Yield: 58%. mp: 181–182 °C. *R*_*f*_ = 0.53 (DCM:MeOH = 14:1). *IR (KBr, cm*^*−1*^*):* 3065 (CH aren); 2957 (CH, CH_2_); 1717, 1676 (C=O); 1614 (C=C). ^1^H-NMR (500 MHz, DMSO-*d*_*6*_): δ 12.50, 12.20 (s, 1H, CONH); 8.45 (s, 1H, CH=N); 8.27, 8.20 (s, 1H, H-2′); 8.13–8.10 (m, 1H, H-6′); 7.93–7.89 (m, 2H, H-4′, H-5′); 7.71 (d, *J* = 7.00 Hz, 1H, H-7); 7.65 (t, *J* = 7.50 Hz, 1H, H-5); 7.44 (t, *J* = 7.50 Hz, 1H, H-6), 7.37 (d, *J* = 8.00 Hz, 1H, H-4), 5.32, 4.90 (s, 1.50H, 0.50H, CH_2_–CONH); 4.06 (s, 2H, H-3a, H-3b). ^13^C-NMR (125 MHz, DMSO-*d*_*6*_): δ 175.65, 175.56, 169.14, 164.46, 146.55, 145.58, 143.33, 137.15, 134.61, 131.61, 130.70, 130.55, 128.34, 127.39, 127.03, 126.77, 126.62, 125.33, 125.08, 122.87, 122.68, 109.66, 109.55, 41.81, 35.92. HR-MS (ESI) *m/z*: 328.0851 {[M + H]^+^, ^35^Cl}; 330.0821 {[M + H]^+^, ^37^Cl}. HR-MS (ESI) *m/z* calculated for C_17_H_15_ClN_3_O_2_ [M + H]^+^ 328.0853. Found 328.0851.

#### (E)-N'-(4-Chlorobenzylidene)-2-(2-oxoindolin-1-yl)acetohydrazide (*4d*)

Yellow solid; Yield: 62%. mp: 185–187 °C. *R*_*f*_ = 0.53 (DCM:MeOH=14:1). *IR (KBr, cm*^*−1*^*):* 3183 (NH); 3061 (CH aren); 2959, 2922, 2851 (CH, CH_2_); 1711, 1674 (C=O); 1616 (C=C). ^1^H-NMR (500 MHz, DMSO-*d*_*6*_): δ 11.76 (s, 1H, CONH); 8.24, 8.04 (s, 1H, CH=N); 7.78, 7.73 (d, *J* = 8.50 Hz, 2H, H-2′, H-6′); 7.53, 7.51 (d, *J* = 8.50 Hz, 2H, H-3′, H-5′); 7.29 (d, *J* = 7.00 Hz, 1H, H-7); 7.23 (t, *J* = 7.50 Hz, 1H, H-5); 7.01 (t, *J* = 7.25 Hz, 1H, H-6); 6.95 (d, *J* = 7.50 Hz, 1H, H-4); 4.89, 4.45 (s, 1.50H, 0.50H, CH_2_–CONH); 3.64 (s, 2H, H-3a, H-3b). ^13^C-NMR (125 MHz, DMSO-*d*_*6*_): δ 175.19, 175.09, 168.57, 163.88, 146.46, 145.15, 144.87, 143.20, 134.89, 133.51, 133.44, 129.37, 129.23, 129.09, 127.90, 125.01, 124.89, 124.73, 124.64, 122.42, 122.23, 109.23, 109.11, 42.02, 41.31, 35.49. HR-MS (ESI) *m/z*: 328.0844 {[M + H]^+^, ^35^Cl}; 330.0813 {[M + H]^+^, ^37^Cl}. HR-MS (ESI) *m/z* calculated for C_17_H_15_ClN_3_O_2_ [M + H]^+^ 328.0853. Found 328.0844.

#### (E)-N'-(4-Methoxybenzylidene)-2-(2-oxoindolin-1-yl)acetohydrazide (*4e*)

Yellow solid; Yield: 51%. mp: 168–169 °C. *R*_*f*_ = 0.47 (DCM:MeOH = 14:1). *IR (KBr, cm*^*−1*^*):* 3188 (NH); 3092, 3065 (CH aren); 2970, 2839 (CH, CH_2_); 1703, 1674 (C=O); 1605 (C=C). ^1^H-NMR (500 MHz, DMSO-*d*_*6*_): δ 11.57 (s, 1H, CONH); 8.19, 7.99 (s, 1H, CH=N); 7.69, 7.65 (d, *J* = 9.00 Hz, 2H, H-2′, H-6′); 7.29 (d, *J* = 7.00 Hz, 1H, H-7); 7.22 (t, *J* = 7.25 Hz, 1H, H-5); 7.04–7.00 (m, 2H, H-4, H-6); 6.94 (d, *J* = 7.50 Hz, 2H, H-3′, H-5′); 4.86, 4.44 (s, 1.50H, 0.50H, CH_2_–CONH); 3.81 (s, 3H, CH_3_); 3.63 (s, 2H, H-3a, H-3b). ^13^C-NMR (125 MHz, DMSO-*d*_*6*_): δ 175.18, 175.08, 168.22, 163.48, 161.38, 161.23, 147.65, 145.20, 144.91, 144.36, 129.20, 129.02, 127.90, 127.07, 125.01, 124.89, 124.72, 124.62, 122.39, 122.20, 144.79, 109.23, 109.11, 55.78, 41.00, 41.27, 35.50. HR-MS (ESI) *m/z*: 324.1342 [M + H]^+^. HR-MS (ESI) *m/z* calculated for C_18_H_18_N_3_O_3_ [M + H]^+^ 324.1348. Found 324.1342.

#### (E)-N'-(2-Hydroxybenzylidene)-2-(2-oxoindolin-1-yl)acetohydrazide (*4f*)

Yellow solid; Yield: 45%. mp: 173–174 °C. *R*_*f*_ = 0.45 (DCM:MeOH = 14:1). *IR (KBr, cm*^*−1*^*):* 3183 (NH); 3061 (CH aren); 2974, 2914, 2839 (CH, CH_2_); 1713, 1670 (C=O); 1611 (C=C). ^1^H-NMR (500 MHz, DMSO-*d*_*6*_): δ 11.90, 11.65 (s, 0.50H, 0.50H, CONH); 10.95, 10.08 (s, 0.50H, 0.50H, OH); 8.46, 8.36 (s, 0.50H, 0.50H, CH=N); 7.77, 7.55 (dd, *J* = 7.75 Hz, *J*′ = 1.75 Hz, 0.50H, 0.50H, H-6′); 7.31–7.21 (m, 3H, H-5, H-7, H-4′); 7.05–6.94 (m, 4H, H-4, H-6, H-3′, H-5′); 4.86, 4.49 (s, 1.00H, 1.00H, CH_2_–CONH); 3.63 (s, 2H, H-3a, H-3b). ^13^C-NMR (125 MHz, DMSO-*d*_*6*_): δ 175.19, 175.14, 168.13, 163.70, 157.76, 156.90, 147.92, 145.18, 144.84, 141.93, 131.97, 131.72, 129.61, 127.95, 127.89, 126.84, 125.04, 124.89, 124.75, 124.62, 122.45, 122.21, 120.57, 119.89, 119.83, 119.10, 116.83, 116.63, 109.26, 109.13, 41.94, 41.24, 40.60, 40.43, 35.50. HR-MS (ESI) *m/z*: 310.1183 [M + H]^+^. HR-MS (ESI) *m/z* calculated for C_17_H_16_N_3_O_3_ [M + H]^+^ 310.1192. Found 310.1183.

#### (E)-N'-(2-Hydroxy-4-methoxybenzylidene)-2-(2-oxoindolin-1-yl)acetohydrazide (*4g*)

Yellow solid; Yield: 52%. mp: 177–178 °C. *R*_*f*_ = 0.43 (DCM:MeOH = 14:1). *IR (KBr, cm*^*−1*^*):* 3184 (NH); 3063 (CH aren); 2972, 2922, 2851 (CH, CH_2_); 1709 1670 (C=O); 1628, 1609 (C=C). ^1^H-NMR (500 MHz, DMSO-*d*_*6*_): δ 11.50 (s, 1H, CONH); 8.37, 8.26 (s, 0.50H, 0.50H, CH=N); 7.66, 7.44 (d, *J* = 8.50 Hz, 0.50H, 0.50H, H-6′); 7.29 (t, *J* = 7.50 Hz, 1H, H-5); 7.23 (dd, *J* = 7.88 Hz, *J*′ = 3.88 Hz, 1H, H-7); 7.05–7.00 (m, 1H, H-4); 6.95 (t, *J* = 7.50 Hz, 1H, H-6); 6.53–6.47 (m, 2H, H-3′, H-5′); 4.83, 4.47 (s, 1.00H, 1.00H, CH_2_–CONH); 3.77 (s, 3H, CH_3_); 3.63 (s, 2H, H-3a, H-3b). ^13^C-NMR (125 MHz, DMSO-*d*_*6*_): δ 175.18, 175.13, 167.78, 163.40, 162.62, 162.41, 159.77, 158.50, 148.55, 145.20, 144.85, 142.47, 131.34, 128.39, 127.94, 127.88, 125.03, 124.88, 124.73, 124.60, 122.43, 122.19, 113.52, 112.11, 109.27, 109.13, 106.93, 106.86, 101.60, 101.40, 55.77, 55.65, 41.90, 41.20, 35.50. HR-MS (ESI) *m/z*: 340.1289 [M + H]^+^. HR-MS (ESI) *m/z* calculated for C_18_H_18_N_3_O_4_ [M + H]^+^ 340.1297. Found 340.1289.

#### (E)-N'-(3-Allyl-2-hydroxybenzylidene)-2-(2-oxoindolin-1-yl)acetohydrazide (*4h*)

Yellow solid; Yield: 64%. mp: 185–186 °C. *R*_*f*_ = 0.55 (DCM:MeOH = 14:1). *IR (KBr, cm*^*−1*^*):* 3190 (NH); 3057 (CH aren); 2974, 2913, 2853 (CH, CH_2_); 1715, 1668 (C=O); 1614 (C=C). ^1^H-NMR (500 MHz, DMSO-*d*_*6*_): δ 11.95 (s, 1H, CONH); 11.65 (s, 1H, OH); 8.40, 8.28 (s, 0.70H, 0.30H, CH = N); 7.42–7.19 (m, 4H, H-5, H-7, H-6′, H-4′); 7.05–6.88 (m, 3H, H-4, H-6, H-5′); 6.01–5.97 (m, 1H, CH=CH_2_); 5.09–5.01 (m, 2H, CH=CH_2_); 4.87, 4.52 (s, 0.60H, 1.40H, CH_2_–CONH); 3.64 (s, 2H, H-3a, H-3b); 3.41, 3.36 (d, *J* = 6.50 Hz, 2H, CH_2_–CH). ^13^C-NMR (125 MHz, DMSO-*d*_*6*_): δ 175.15, 67.60, 163.79, 155.93, 154.85, 150.22, 146.59, 145.10, 144.81, 136.98, 132.22, 129.75, 128.65, 127.95, 127.85, 127.81, 127.61, 125.04, 124.88, 124.77, 124.61, 122.48, 122.25, 120.22, 119.59, 119.12, 117.75, 116.30, 116.18, 109.41, 109.14, 41.91, 41.09, 35.50, 35.75. HR-MS (ESI) *m/z*: 350.1500 [M + H]^+^. HR-MS (ESI) *m/z* calculated for C_20_H_20_N_3_O_3_ [M + H]^+^ 350.1505. Found 350.1500.

#### (E)-N'-Benzylidene-2-(5-fluoro-2-oxoindolin-1-yl)acetohydrazide (*4i*)

Yellow solid; Yield: 62%. mp: 173–174 °C. *R*_*f*_ = 0.55 (DCM:MeOH = 14:1). *IR (KBr, cm*^*−1*^*):* 3184 (NH); 3090 (CH aren); 2968, 2918, 2849 (CH, CH_2_); 1717, 1674 (C=O); 1607 (C=C). ^1^H-NMR (500 MHz, DMSO-*d*_*6*_): δ 11.71 (s, 1H, CONH); 8.24, 8.05 (s, 1H, CH=N); 7.75, 7.71 (dd, *J* = 7.25 Hz, *J*′ = 1.75 Hz, 2H, H-2′, H-6′); 7.48–7.44 (m, 3H, H-3′, H-4′, H-5′); 7.20 (dd, *J* = 8.50 Hz, *J*′ = 2.50 Hz, 1H, H-7); 7.07 (td, *J* = 9.13 Hz, *J*′ = 2.33 Hz, 1H, H-6); 6.97 (dd, *J* = 8.50 Hz, *J*′ = 4.00 Hz, 1H, H-4); 4.89, 4.47 (s, 1.50H, 0.50H, CH_2_–CONH); 3.68 (s, 2H, H-3a, H-3b). ^13^C-NMR (125 MHz, DMSO-*d*_*6*_): δ 174.98, 174.88, 168.44, 163.70, 159.57, 157.69, 147.78, 144.50, 141.46, 141.20, 134.52, 134.45, 130.65, 130.48, 129.30, 127.59, 127.42, 126.88, 126.80, 126.73, 114.07, 113.89, 112.80, 112.67, 112.60, 112.47, 109.97, 109.91, 109.78, 42.14, 41.47, 40.61, 40.44, 35.88. HR-MS (ESI) *m/z*: 312.1139 [M + H]^+^. HR-MS (ESI) *m/z* calculated for C_17_H_15_FN_3_O_2_ [M + H]^+^ 312.1148. Found 312.1139.

#### (E)-N'-(2-Chlorobenzylidene)-2-(5-fluoro-2-oxoindolin-1-yl)acetohydrazide (*4j*)

Yellow solid; Yield: 65%. mp: 185–186 °C. *R*_*f*_ = 0.57 (DCM:MeOH = 14:1). *IR (KBr, cm*^*−1*^*):* 3183 (NH); 3096 (CH aren); 2953, 2918, 2851 (CH, CH_2_); 1680 (C=O); 1607 (C=C). ^1^H-NMR(500 MHz, DMSO-*d*_*6*_): δ 11.95, 11.89 (s, 1H, CONH); 8.63, 8.44 (s, 1H, CH=N); 8.07, 7.94 (dd, 1H, *J* = 7.50 Hz, *J*′ = 2.00 Hz, H-6′); 7.56–7.42 (m, 3H, H-3′, H-4′, H-5′), 7.20 (dd, *J* = 8.00 Hz, *J*′ = 2.50 Hz, 1H, H-7); 7.07 (td, *J* = 9.25 Hz, *J*′ = 2.50 Hz, 1H, H-6); 6.98 (dd, *J* = 8.50 Hz, *J*′ = 4.50 Hz, 1H, H-4); 4.91, 4.81 (s, 1H, CH_2_–CONH); 3.68 (s, 2H, H-3a, H-3b). ^13^C-NMR (125 MHz, DMSO-*d*_*6*_): δ 174.99, 174.90, 168.61, 163.91, 159.58, 157.70, 143.66, 141.41, 140.54, 133.65, 133.47, 132.11, 131.91, 131.72, 130.39, 128.15, 128.08, 127.51, 127.38, 126.80, 126.73, 114.08, 113.90, 112.69, 112.63, 112.49, 109.98, 109.92, 55.38, 42.20, 41.51, 35.87. HR-MS (ESI) *m/z*: 346.0753 {[M + H]^+^, ^35^Cl}; 348.0723 {[M + H]^+^, ^37^Cl}. HR-MS (ESI) *m/z* calculated for C_17_H_14_ClFN_3_O_2_ [M + H]^+^ 346.0759. Found 346.0753.

#### (E)-N'-(3-Chlorobenzylidene)-2-(5-fluoro-2-oxoindolin-1-yl)acetohydrazide (*4k*)

Yellow solid; Yield: 58%. mp: 187–188 °C. *R*_*f*_ = 0.57 (DCM:MeOH = 14:1). *IR (KBr, cm*^*−1*^*):* 3183 (NH); 3088 (CH aren); 2918, 2851 (CH, CH_2_); 1719, 1676 (C=O); 1607 (C=C). ^1^H-NMR (500 MHz, DMSO-*d*_*6*_): δ 11.82 (s, 1H, CONH); 8.23, 8.03 (s, 1H, CH=N); 7.84, 7.76 (s, 1H, H-2′); 7.70–7.68 (m, 1H, H-6′); 7.51–7.47 (m, 2H, H-4′, H-5′); 7.20 (dd, *J* = 8.00 Hz, *J*′ = 2.00 Hz, 1H, H-7); 7.07 (td, *J* = 9.13 Hz, *J*′ = 2.17 Hz, 1H, H-6); 6.97 (dd, *J* = 8.50 Hz, *J*′ = 4.50 Hz, 1H, H-4); 4.91, 4.48 (s, 2H, CH_2_–CONH); 3.68 (s, 2H, H-3a, H-3b). ^13^C-NMR (125 MHz, DMSO-*d*_*6*_): δ 174.99, 174.89, 168.65, 163.94, 159.57, 157.69, 146.11, 142.88, 141.44, 136.80, 136.71, 134.18, 134.09, 131.21, 131.17, 130.27, 130.12, 126.97, 126.80, 126.73, 126.58, 126.35, 126.17, 114.07, 113.89, 112.69, 112.61, 112.49, 109.97, 109.90, 109.78, 42.14, 41.57, 35.87. HR-MS (ESI) *m/z*: 346.0751 {[M + H]^+^, ^35^Cl}; 348.0720 {[M + H]^+^, ^37^Cl}. HR-MS (ESI) *m/z* calculated for C_17_H_14_ClFN_3_O_2_ [M + H]^+^ 346.0759. Found 346.0751.

#### (E)-N'-(4-Chlorobenzylidene)-2-(5-fluoro-2-oxoindolin-1-yl)acetohydrazide (*4l*)

Yellow solid; Yield: 63%. mp: 191–192 °C. *R*_*f*_ = 0.57 (DCM:MeOH = 14:1). *IR (KBr, cm*^*−1*^*):* 3183 (NH); 3094 (CH aren); 2968, 2853 (CH, CH_2_); 1714, 1672 (C=O); 1611 (C=C). ^1^H-NMR (500 MHz, DMSO-*d*_*6*_): δ 11.77 (s, 1H, CONH); 8.24, 8.04 (s, 1H, CH=N); 7.78, 7.73 (d, *J* = 8.75 Hz, 1.50H, 0.50H, H-2′, H-6′); 7.52 (d, *J* = 8.50 Hz, 2H, H-3′, H-5′); 7.20 (dd, *J* = 8.00 Hz, *J*′ = 2.00 Hz, 1H, H-7); 7.06 (td, *J* = 9.13 Hz, *J*′ = 2.17 Hz, 1H, H-6); 6.97 (dd, *J* = 9.00 Hz, *J*′ = 4.50 Hz, 1H, H-4); 4.89, 4.47 (s, 1.50H, 0.50H, CH_2_–CONH); 3.68 (s, 2H, H-3a, H-3b). ^13^C-NMR (125 MHz, DMSO-*d*_*6*_): δ 174.98, 174.88, 168.52, 157.69, 146.47, 143.21, 141.45, 135.09, 134.90, 133.42, 129.37, 129.23, 129.09, 126.80, 126.72, 114.07, 113.89, 112.68, 112.48, 109.97, 109.90, 43.14, 41.48, 35.87. HR-MS (ESI) *m/z*: 346.0751 {[M + H]^+^, ^35^Cl}; 348.0721 {[M + H]^+^, ^37^Cl}. HR-MS (ESI) *m/z* calculated for C_17_H_14_ClFN_3_O_2_ [M + H]^+^ 346.0759. Found 346.0751.

#### (E)-N'-(4-Methoxybenzylidene)-2-(5-fluoro-2-oxoindolin-1-yl)acetohydrazide (*4m*)

Yellow solid; Yield: 60%. mp: 175–176 °C. *R*_*f*_ = 0.53 (DCM:MeOH = 14:1). *IR (KBr, cm*^*−1*^*):* 3184 (NH); 3088 (CH aren); 2936, 2837 (CH, CH_2_); 1709, 1670 (C=O); 1607 (C=C). ^1^H-NMR (500 MHz, DMSO-*d*_*6*_): δ 11.57 (s, 1H, CONH), 8.18, 7.99 (s, 1H, CH=N); 7.68, 7.65 (d, *J* = 9.00 Hz, 2H, H-2′, H-6′); 7.20 (d, *J* = 7.00 Hz, 1H, H-7); 7.06 (t, *J* = 9.25 Hz, 1H, H-6); 7.01 (d, *J* = 9.00 Hz, 2H, H-3′, H-5′); 6.96 (dd, *J* = 7.75 Hz, *J*′ = 4.25 Hz, 1H, H-4); 4.90, 4.44 (s, 1.50H, 0.50H, CH_2_-CONH); 3.81 (s, 3H, CH_3_); 3.67 (s, 2H, H-3a, H-3b). ^13^C-NMR (125 MHz, DMSO-*d*_*6*_): δ 174.97, 174.86, 168.16, 163.40, 161.38, 161.23, 159.55, 157.67, 147.67, 144.38, 141.50, 141.22, 129.21, 129.01, 127.05, 126.79, 126.71, 1114.79, 114.06, 113.88, 112.78, 112.66, 112.59, 112.46, 109.96, 109.90, 109.82, 55.79, 42.12, 41.45, 35.88. HR-MS (ESI) *m/z*: 342.1247 [M + H]^+^. HR-MS (ESI) *m/z* calculated for C_18_H_17_FN_3_O_3_ [M + H]^+^ 342.1254. Found 342.1247.

#### (E)-N'-(2-Hydroxybenzylidene)-2-(5-fluoro-2-oxoindolin-1-yl)acetohydrazide (*4n*)

Yellow solid; Yield: 55%. mp: 173–174 °C. *R*_*f*_ = 0.51 (DCM:MeOH = 14:1). *IR (KBr, cm*^*−1*^*):* 3188 (NH); 3063 (CH aren); 2922, 2853 (CH, CH_2_); 1719, 1670 (C=O); 1622 (C=C). ^1^H-NMR (500 MHz, DMSO-*d*_*6*_): δ 11.90, 11.75 (0.50H, 0.50H, CONH); 10.95, 10.05 (s, 0.50H, 0.50H, OH); 8.46, 8.35 (s, 0.50H, 0.50H, CH=N); 7.76, 7.56 (dd, 0.50H, 0.50H, H-6′); 7.31–7.25 (m, 1H, H-4′); 7.21 (td, *J* = 9.38 Hz, *J*′ = 3.00 Hz, 1H, H-5′); 7.11–7.05 (m, 1H, H-3′); 6.99–6.86 (m, 3H, H-4, H-6, H-7); 4.87, 4.49 (s, 1.00H, 1.00H, CH_2_–CONH); 3.67 (s, 2H, H-3a, H-3b). ^13^C-NMR (125 MHz, DMSO-*d*_*6*_): δ 159.56, 157.78, 157.75, 157.68, 156.90, 147.91, 141.95, 141.48, 141.13, 131.98, 131.73, 129.59, 126.98, 126.90, 126.83, 126.79, 126.71, 120.55, 119.89, 119.84, 119.10, 116.82, 116.62, 114.13, 114.06, 113.94, 113.88, 112.81, 112.65, 112.61, 112.45, 109.99, 104.93, 109.86, 109.80, 42.06, 41.43, 35.88. HR-MS (ESI) *m/z*: 328.1087 [M + H]^+^. HR-MS (ESI) *m/z* calculated for C_17_H_15_FN_3_O_3_ [M + H]^+^ 328.1097. Found 328.1087.

#### (E)-N'-(2-Hydroxy-4-methoxybenzylidene)-2-(5-fluoro-2-oxoindolin-1-yl)acetohydrazide (*4o*)

Yellow solid; Yield: 52%. mp: 186–187 °C. *R*_*f*_ = 0.53 (DCM:MeOH = 14:1). *IR (KBr, cm*^*−1*^*):* 3177 (NH); 3065 (CH aren); 2926 (CH, CH_2_); 1715, 1668 (C=O); 1607 (C=C). ^1^H-NMR (500 MHz, DMSO-*d*_*6*_): δ 11.80, 11.51 (s, 0.50H, 0.50H, CONH); 11.27, 10.17 (s, 0.50H, 0.50H, OH); 8.36, 8.25 (s, 0.50H, 0.50H, CH=N); 7.66, 7.44 (d, *J* = 9.00 Hz, 0.50H, 0.50H, H-6′); 7.20 (td, *J* = 9.50 Hz, *J*′ = 2.50 Hz, 1H, H-6); 7.12–7.04 (m, 1H, H-7); 6.98–6.95 (m, 1H, H-4); 6.53–6.47 (m, 2H, H-3′, H-5′); 4.83, 4.47 (s, 1.00H, 1.00H, CH_2_-CONH); 3.77 (s, 3H, –OCH_3_); 3.63 (s, 2H, H-3a, H-3b). ^13^C-NMR (125 MHz, DMSO-*d*_*6*_): δ 175.19, 163.07, 140.82, 132.73, 132.24, 127.96, 124.96, 124.75, 122.47, 121.72, 120.09, 11.46, 109.29, 35.44. HR-MS (ESI) *m/z*: 358.1195 [M + H]^+^. HR-MS (ESI) *m/z* calculated for C_18_H_17_FN_3_O_4_ [M + H]^+^ 358.1203. Found 358.1195.

#### (E)-N'-(3-Allyl-2-hydroxybenzylidene)-2-(5-fluoro-2-oxoindolin-1-yl)acetohydrazide (*4p*)

Yellow solid; Yield: 65%. mp: 194–195 °C. *R*_*f*_ = 0.57 (DCM:MeOH = 14:1). *IR (KBr, cm*^*−1*^*):* 3061 (CH aren); 2974, 2913 (CH, CH_2_); 1715, 1668 (C=O); 1607 (C=C). ^1^H-NMR (500 MHz, DMSO-*d*_*6*_): δ 12.10, 11.85 (s, 1H, 0.25H, CONH); 11.67, 10.15 (s, 1H, OH); 8.39, 8.27 (s, 1H, CH=N); 7.40, 7.34 (dd, *J* = 8.00 Hz, *J*′ = 1.50 Hz, 1H, H-6′); 7.23–7.19 (m, 2H, H-4′, H-5′); 7.11–6.88 (m, 3H, H-4, H-6, H-7); 6.00–5.94 (m, 1H, CH=CH_2_); 5.09–5.01 (m, 2H, CH=CH_2_); 4.87, 4.52 (s, 0.60H, 1.40H, CH_2_–CONH); 3.68 (s, 2H, H-3a, H-3b); 3.40, 3.36 (d, *J* = 6.50 Hz, 2H, CH_2_–CH). ^13^C-NMR (125 MHz, DMSO-*d*_*6*_): δ 159.59, 157.80, 155.91, 154.85, 150.24, 146.63, 141.37, 141.10, 136.97, 132.24, 129.76, 128.67, 127.80, 127.61, 126.98, 126.91, 126.78, 126.70, 120.22, 119.60, 119.07, 117.73, 116.30, 116.19, 114.14, 114.04, 113.96, 113.85, 112.83, 112.63, 112.45, 110.14, 110.78, 109.87, 109.81, 42.02, 41.27, 40.60, 35.89, 33.75. HR-MS (ESI) *m/z*: 368.1403 [M + H]^+^. HR-MS (ESI) *m/z* calculated for C_20_H_19_FN_3_O_3_ [M + H]^+^ 368.1410. Found 368.1403.

#### (Z)-2-(2-Oxoindolin-1-yl)-N'-(2-oxoindolin-3-ylidene)acetohydrazide (*5a*)

Yellow solid; Yield: 58%. mp: 191–192 °C. *R*_*f*_ = 0.61 (DCM:MeOH = 14:1). *IR (KBr, cm*^*−1*^*):* 3123 (NH); 3086 (CH aren); 2984, 2920, 2851, 2806 (CH, CH_2_); 1695, 1663 (C=O); 1612 (C=C). ^1^H-NMR (500 MHz, DMSO-*d*_*6*_): δ 12.67 (s, 1H, NH-1′); 11.29 (s, 1H, CONH); 7.60 (s, 1H, H-4′); 7.41 (t, *J* = 7.50 Hz, 1H, H-6′); 7.31 (d, *J* = 7.50 Hz, 1H, H-7); 7.24 (t, *J* = 7.75 Hz, 1H, H-5); 7.12 (t, *J* = 7.50 Hz, 1H, H-5′); 7.03 (t, *J* = 8.50 Hz, 1H, H-6); 7.02 (d, *J* = 8.50 Hz, 1H, H-7′); 6.79 (d, *J* = 7.50 Hz, 1H, H-4); 5.05, 4.73 (s, 1.60H, 0.40H, CH_2_–CONH); 3.67 (s, 2H, H-3a, H-3b). ^13^C-NMR (125 MHz, DMSO-*d*_*6*_): δ 175.19, 163.00, 143.11, 132.33, 127.97, 124.96, 124.76, 123.12, 122.50, 121.40, 120.10, 111.70, 109.29, 35.45. HR-MS (ESI) *m/z*: 335.1141 [M + H]^+^. HR-MS (ESI) *m/z* calculated for C_18_H_15_N_4_O_3_ [M + H]^+^ 335.1144. Found 335.1141.

#### (Z)-N'-(5-Chloro-2-oxoindolin-3-ylidene)-2-(2-oxoindolin-1-yl)acetohydrazide (*5b*)

Yellow solid; Yield: 64%. mp: 201–202 °C. *R*_*f*_ = 0.65 (DCM:MeOH = 14:1). *IR (KBr, cm*^*−1*^*):* 3063 (CH aren); 2976 (CH, CH_2_); 1701, 1668 (C=O); 1614 (C=C). ^1^H-NMR (500 MHz, DMSO-*d*_*6*_): δ 7.61 (s, 1H, H-4′); 7.44, 7.42 (dd,* J* = 8.25 Hz, *J*′ = 2.25 Hz, 1H, H-6′); 7.31 (d, *J* = 7.50 Hz, 1H, H-7), 7.25 (t, *J* = 7.75 Hz, 1H, H-5), 7.04 (t, *J* = 7.50 Hz, 1H, H-6); 7.02 (d, *J* = 8.00 Hz, 1H, H-7′); 6.97 (d, *J* = 8.00 Hz, 1H, H-4); 5.00 (s, 2H, CH_2_–CONH); 3.67 (s, 2H, H-3a, H-3b). ^13^C-NMR (125 MHz, DMSO-*d*_*6*_): δ 175.20, 162.99, 142.23, 131.60, 127.97, 127.13, 124.98, 124.78, 122.52, 122.02, 120.99, 113.27, 109.24, 35.45. HR-MS (ESI) *m/z*: 369.0751 {[M + H]^+^, ^35^Cl}; 371.0715 {[M + H]^+^, ^37^Cl}. HR-MS (ESI) *m/z* calculated for C_18_H_14_ClN_4_O_3_ [M + H]^+^ 369.0754. Found 369.0751.

#### (Z)-N'-(5-Methyl-2-oxoindolin-3-ylidene)-2-(2-oxoindolin-1-yl)acetohydrazide (*5c*)

Yellow solid; Yield: 67%. mp: 198–199 °C. *R*_*f*_ = 0.63 (DCM:MeOH = 14:1). *IR (KBr, cm*^*−1*^*):* 3146 (NH); 2984, 2938 (CH, CH_2_); 1690, 1665 (C=O); 1612 (C=C). ^1^H-NMR (500 MHz, DMSO-*d*_*6*_): δ 13.21, 12.68 (s, 0.30H, 0.70H, NH-1′); 11.81 (s, 1H, CONH); 7.41 (s, 1H, H-4′); 7.31 (d, *J* = 7.00 Hz, 1H, H-6′); 7.24 (t, *J* = 7.75 Hz, 1H, H-5); 7.21 (d, *J* = 8.00 Hz, 1H, H-7); 7.04 (t, *J* = 7.50 Hz, 1H, H-6); 7.02 (d, *J* = 8.00 Hz, 1H, H-7′); 6.85 (d, *J* = 7.00 Hz, 1H, H-4); 5.04, 4.71 (s, 1.40H, 0.60H, CH_2_–CONH); 3.67 (s, 2H, H-3a, H-3b); 2.31 (s, 3H, CH_3_). ^13^C-NMR (125 MHz, DMSO-*d*_*6*_): δ 175.19, 169.37, 163.01, 144.84, 140.82, 135.71, 132.73, 132.24, 127.96, 124.97, 124.76, 122.47, 121.73, 120.09, 111.46, 109.28, 35.44, 20.98. HR-MS (ESI) *m/z*: 349.1304 [M + H]^+^. HR-MS (ESI) *m/z* calculated for C_19_H_17_N_4_O_3_ [M + H]^+^ 349.1301. Found 349.1304.

#### (Z)-2-(5-Fluoro-2-oxoindolin-1-yl)-N'-(2-oxoindolin-3-ylidene)acetohydrazide (*5d*)

Yellow solid; Yield: 55%. mp: 215–216 °C. *R*_*f*_ = 0.63 (DCM:MeOH = 14:1). *IR (KBr, cm*^*−1*^*):* 3246 (NH); 2982, 2886 (CH, CH_2_); 1732, 1694 (C=O); 1620 (C=C). ^1^H-NMR (500 MHz, DMSO-*d*_*6*_): δ 13.18, 12.68 (s, 0.30H, 0.70H, NH-1′); 11.29 (s, 1H, CONH); 7.61, 7.51 (d, *J* = 8.00 Hz, 0.70H, 0.30H, H-4′); 7.41 (t, *J* = 7.50 Hz, 1H, H-6′); 7.25, 7.24 (dd, *J* = 8.00 Hz, *J*′ = 1.50 Hz, 1H, H-7); 7.19, 7.17 (d, *J* = 8.50 Hz, 1H, H-6); 7.13, 7.12 (d, *J* = 8.25 Hz, 1H, H-4), 7.05 (t, *J* = 10.25 Hz, 1H, H-5′); 6.97 (d, *J* = 7.50 Hz, 1H, H-7′); 5.18, 5.15 (s, 1.40H, 0.60H, CH_2_–CONH); 4.86 (s, 2H, H-3a, H-3b). ^13^C-NMR (125 MHz, DMSO-*d*_*6*_): δ 162.97, 161.49, 143.11, 132.35, 123.13, 121.42, 120.07, 113.56, 113.36, 111.70, 110.70. HR-MS (ESI) *m/z*: 353.1043 [M + H]^+^. HR-MS (ESI) *m/z* calculated for C_18_H_14_FN_4_O_3_ [M + H]^+^ 353.1050. Found 353.1043.

#### (Z)-N'-(5-Chloro-2-oxoindolin-3-ylidene)-2-(5-fluoro-2-oxoindolin-1-yl)acetohydrazide (*5e*)

Yellow solid; Yield: 64%. mp: 221–222 °C. *R*_*f*_ = 0.65 (DCM:MeOH = 14:1). *IR (KBr, cm*^*−1*^*):* 3065 (CH aren); 2978 (CH, CH_2_); 1701, 1668 (C=O); 1622 (C=C). ^1^H-NMR (500 MHz, DMSO-*d*_*6*_): δ 12.57 (s, 1H, NH-1′); 11.40 (s, 1H, CONH); 7.62 (s, 1H, H-4′); 7.45, 7.43 (dd, *J* = 8.50 Hz, *J*′ = 2.00 Hz, 1H, H-6′); 7.22 (d, *J* = 7.50 Hz, 1H, H-7); 7.09 (td, *J* = 9.00 Hz, *J*′ = 2.00 Hz, 1H, H-6); 7.04, 7.03 (d, *J* = 8.50 Hz, 1H, H-4); 6.98 (d, *J* = 8.00 Hz, 1H, H-7′); 5.05 (s, 2H, CH_2_–CONH); 3.71 (s, 2H, H-3a, H-3b). ^13^C-NMR (125 MHz, DMSO-*d*_*6*_): δ 174.98, 169.50, 162.74, 159.71, 157.83, 141.75, 131.64, 127.28, 126.91, 126.84, 121.87, 121.03, 114.06, 113.98, 113.21, 112.84, 112.65, 110.00, 109.93, 35.83. HR-MS (ESI) *m/z*: 387.0657 {[M + H]^+^, ^35^Cl}; 389.0627 {[M + H]^+^, ^37^Cl}. HR-MS (ESI) *m/z* calculated for C_18_H_13_ClFN_4_O_3_ [M + H]^+^ 387.0660. Found 387.0657.

#### (Z)-2-(5-Fluoro-2-oxoindolin-1-yl)-N'-(5-methyl-2-oxoindolin-3-ylidene)acetohydrazide (*5f*)

Yellow solid; Yield: 68%. mp: 217–218 °C. *R*_*f*_ = 0.63 (DCM:MeOH = 14:1). *IR (KBr, cm*^*−1*^*):* 3177 (NH); 3065 (CH aren); 2922 (CH, CH_2_); 1678, 1667 (C=O); 1628, 1609 (C=C). ^1^H-NMR (500 MHz, DMSO-*d*_*6*_): δ 13.20, 12.67 (s, 1H, NH-1′); 11.18 (s, 1H, CONH); 7.40 (s, 1H, H-4′); 7.21 (d, *J* = 7.50 Hz, 2H, H-6′, H-7); 7.09 (td, *J* = 9.13 Hz, *J*′ = 2.25 Hz, 1H, H-6); 7.05, 7.03 (d, *J* = 8.00 Hz, 0.70H, 0.30H, H-4); 6.85 (d, *J* = 7.50 Hz, 1H, H-7′); 5.04, 4.70 (s, 1.40H, 0.60H, CH_2_–CONH), 3.71(s, 2H, H-3a, H-3b), 2.31 (s, 3H, CH_3_). ^13^C-NMR (125 MHz, DMSO-*d*_*6*_): δ 174.97, 163.07, 157.83, 140.83, 132.74, 132.24, 126.89, 121.72, 120.08, 114.16, 113.98, 112.83, 111.47, 110.05, 109.99, 35.83, 20.97. HR-MS (ESI) *m/z*: 367.1203 [M + H]^+^. HR-MS (ESI) *m/z* calculated for C_19_H_16_FN_4_O_3_ [M + H]^+^ 367.1206. Found 367.1203.

### Bioactivity

Two series of compounds synthesized (**4a–p** and **5a–f**) were evaluated for their cytotoxicity against three human cancer cell lines, including SW620 (colon cancer), PC3 (prostate cancer), NCI-H23 (lung cancer), using SRB method as described previously^22^ with slight modifications^23–26^. 5-Fluorouracil (5-FU) and PAC-1 were included in the assay as positive controls. The results expressed as IC_50_ values are summarized in Table 1.

**Table 1:** Cytotoxicity of the selected compounds against some human cancer cell lines.

Cpd code	R	R′	MW	LogP^a^	Cytotoxicity (IC_50_,^b^ μM)/Cell^c^			
					SW620	PC3	NCI-H23	MSCs
**4a**	–H	–H	293.12	1.62	10.12 ± 0.91	12.22 ± 0.99	10.44 ± 0.87	> 30
**4b**	–H	2-Cl	328.08	2.26	9.91 ± 0.76	8.72 ± 0.71	8.34 ± 0.52	> 30
**4c**	–H	3-Cl	328.08	2.26	15.32 ± 1.01	14.23 ± 1.00	13.54 ± 1.11	> 30
**4d**	–H	4-Cl	328.08	2.26	16.33 ± 1.21	17.45 ± 1.34	15.64 ± 1.43	> 30
**4e**	–H	4-OCH_3_	323.13	1.70	9.01 ± 0.74	7.43 ± 0.45	8.72 ± 0.33	> 30
**4f**	–H	2-OH	309.11	1.86	4.77 ± 0.53	7.34 ± 0.04	4.30 ± 0.41	23.21 ± 0.26
**4g**	–H	2-OH-4-OCH_3_	339.12	1.94	3.34 ± 0.31	4.32 ± 0.34	2.59 ± 0.21	15.50 ± 0.64
**4h**	–H	2-OH-3-Allyl	349.14	3.25	4.21 ± 0.17	5.65 ± 0.19	3.05 ± 0.22	15.10 ± 0.93
**4i**	–F	-H	311.11	1.82	9.54 ± 0.74	8.56 ± 0.52	8.44 ± 0.67	> 30
**4j**	–F	2-Cl	346.07	2.46	7.56 ± 0.57	8.43 ± 0.59	8.00 ± 0.98	> 30
**4k**	–F	3-Cl	346.07	2.46	16.45 ± 1.19	16.43 ± 1.56	17.43 ± 1.54	> 30
**4l**	–F	4-Cl	346.07	2.46	14.32 ± 1.21	17.78 ± 1.58	16.44 ± 1.77	> 30
**4m**	–F	4-OCH_3_	341.12	1.90	7.52 ± 0.63	7.33 ± 0.42	7.43 ± 0.56	> 30
**4n**	–F	2-OH	327.10	2.06	4.72 ± 0.75	5.08 ± 0.26	3.70 ± 0.40	21.01 ± 0.51
**4o**	–F	2-OH-4-OCH_3_	357.11	2.14	**1.88 ± 0.02**	**1.83 ± 0.07**	**1.00 ± 0.01**	**12.84** ± 0.71
**4p**	–F	2-OH-3-Allyl	367.13	3.45	3.65 ± 0.23	5.11 ± 0.08	2.70 ± 0.15	15.72 ± 0.46
**5a**	–H	-H	334.11	0.79	16.44 ± 1.05	14.32 ± 1.21	15.33 ± 1.32	> 30
**5b**	–H	5-Cl	369.07	1.43	17.45 ± 1.43	18.45 ± 1.53	16.54 ± 1.33	> 30
**5c**	–H	5-CH_3_	348.12	1.34	17.55 ± 1.32	19.56 ± 2.01	18.44 ± 1.87	> 30
**5d**	–F	-H	352.10	0.99	12.44 ± 1.21	14.55 ± 1.27	13.59 ± 1.31	> 30
**5e**	–F	5-Cl	387.06	1.63	15.32 ± 1.50	14.68 ± 1.71	15.49 ± 1.36	> 30
**5f**	–F	5-CH_3_	366.11	1.54	12.71 ± 1.22	15.33 ± 1.70	14.88 ± 1.83	> 30
**5-FU**^d^			130.08	− 0.81	8.84 ± 1.92	13.61 ± 0.46	13.45 ± 3.92	
**PAC-1**^e^			392.49	3.43	5.43 ± 0.18	4.11 ± 0.39	5.11 ± 0.22	9.22 ± 0.12

Significant values are in bold.

^a^Calculated by EPI 320 software; ^b^The concentration (M) of compounds that produces a 50% reduction in enzyme activity or cell growth. Data represent the mean ± SEM of 3 independent experiments, each performed in triplicate.; ^c^Cells: SW620, colon cancer; PC3, prostate cancer; NCI-H23, lung cancer; MSC, human bone marrow-derived mesenchymal stem cells; ^d^5-FU: 5-Fluorouracil, a positive control. Data referred from ref. 20 for relative comparison purpose; ^e^PAC-1: the first procaspase activating compound, a positive control. Please see the Fig. 2 for the R and R′.

From the results in Table 1, all compounds exhibited strong cytotoxicity against three cancer cell lines. Overally, 5-fluorinated compounds (**4i–p** and **5d–f**) were slightly more cytotoxic than non-fluorinated ones (**4a–h**, **5a–c**). For compounds in series **4a–p**, it was observed that, electron-releasing substituents (–OCH_3_, OH) were generally better than electron-withdrawing groups (–Cl) for cytotoxicity. Especially, compounds with 2-OH substituents produced the best cytotoxicity. The addition of either 4-OCH_3_ or 3-allyl groups further enhanced cytotoxicity of the related compounds (**4g, 4h** and **4o, 4p**). Very interestingly, a 2-hydroxy-4-methoxy substituted pattern was shown to be more favorable for cytotoxicity than 2-hydroxy-3-allyl substituent, which was present in PAC-1. Compound **4o**, which was 5-fluorinated on the 2-oxoindoline part and bearing 2-hydroxy-4-methoxy substituent on the phenyl ring, was the most potent one in term of cytotoxicity. Its IC_50_ values were 1.88 ± 0.02, 1.83 ± 0.07, and 1.00 ± 0.01 μM in SW620, PC-3, and NCI-H23 cell lines, respectively. These values were approximately three- to five-fold lower than that of PAC-1. However, our compounds showed much higher IC_50_ values on normal mesenchymal stem cells than on cancer cells (Table 1).

**Figure 3:**
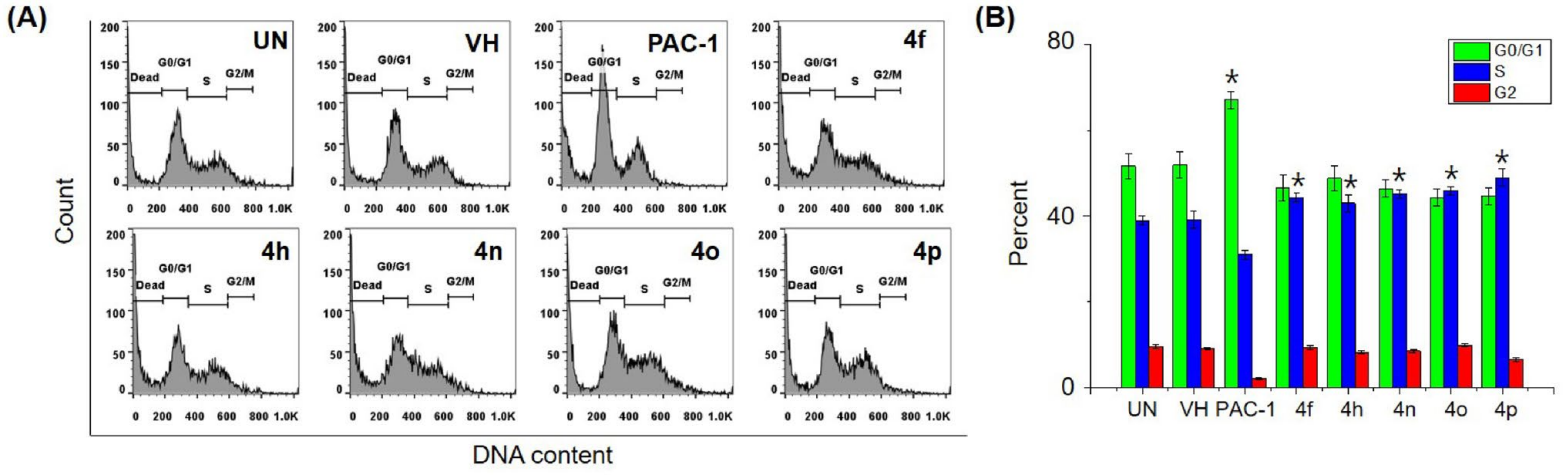
Cell cycle analysis after treatment with some compounds. U937 human lymphoma cells were treated with compounds (50 µM) for 24 h. The harvested cells were stained with propidium iodide (PI) in the presence of RNase and then were analyzed for DNA content by using flow cytometry. Ten thousand cells were acquired (n = 3). *UN* untreated, *VH* vehicle (DMSO. 0.1%). Representative histograms (**A**) and bar graphs (**B**) are shown. *p* values were calculated using one-way ANOVA in GraphPad Software (San Diego, CA, USA). *p < 0.01 vs VH control.

**Figure 4:**
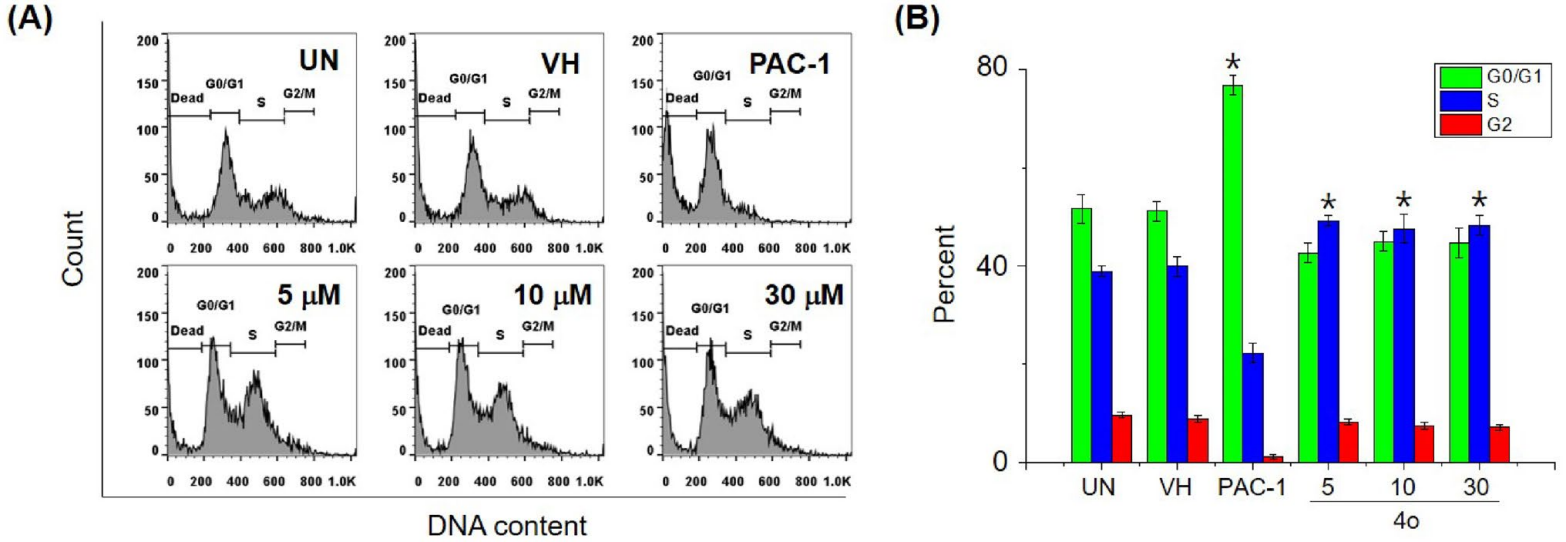
Cell cycle analysis under treatment with different concentrations of compound **4o**. U937 human lymphoma cells were treated with compound **4o** at 5, 10 and 30 µM or PAC-1 (30 µM) for 24 h. The harvested cells were stained with propidium iodide (PI) in the presence of RNase and then were analyzed for DNA content by using flow cytometry. Ten thousand cells were acquired (n = 3). *UN* untreated, *VH* vehicle (DMSO. 0.1%). Representative histograms (**A**) and bar graphs (**B**) are shown. *p* values were calculated using one-way ANOVA in GraphPad Software (San Diego, CA, USA). *p < 0.01 vs VH control.

**Figure 5:**
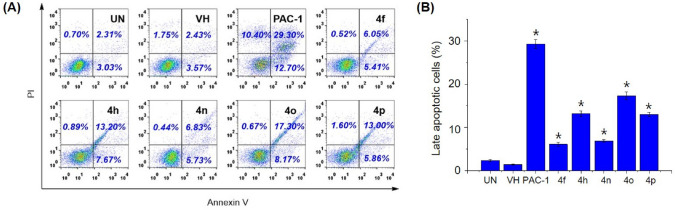
Apoptosis **(Annexin V/PI)** analysis of cells after treatment with some compounds. U937 cells were treated with compounds (50 µM) for 24 h. The harvested cells were incubated with Annexin V-FITC and PI and analyzed by using flow cytometry. Ten thousand cells were acquired (n = 3). *UN* untreated, *VH* vehicle (DMSO. 0.1%). Representative dot plots (**A**). Live cells, left down in dot plot. Early apoptotic cells, right down. Necrotic cells, left up, Late apoptotic cells, right up. Bar graph of late apoptotic cells (**B**). *p* values were calculated using one-way ANOVA in GraphPad Software (San Diego, CA, USA). *p < 0.01 vs VH control.

**Figure 6:**
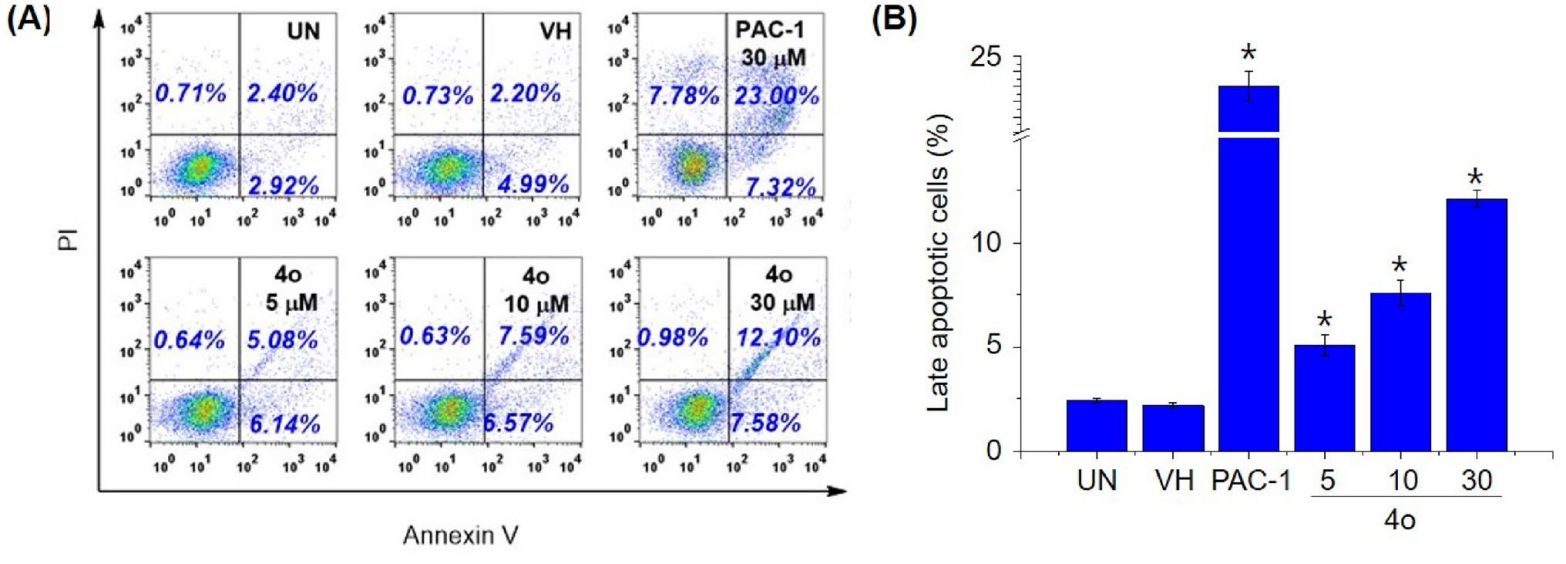
Apoptosis **(Annexin V/PI)** analysis of cells after treatment with different concentrations of compound **4o**. U937 cells were treated with compound **4o** at 5, 10 and 30 µM or PAC-1 (30 µM) for 24 h. The harvested cells were incubated with Annexin V-FITC and PI and analyzed by using flow cytometry. Ten thousand cells were acquired (n = 3). *UN* untreated, *VH* vehicle (DMSO. 0.1%). Representative dot plots (**A**). Live cells, left down in dot plot. Early apoptotic cells, right down. Necrotic cells, left up, Late apoptotic cells, right up. Bar graph of late apoptotic cells (**B**). *p* values were calculated using one-way ANOVA in GraphPad Software (San Diego, CA, USA). *p < 0.01 vs VH control.

**Figure 7:**
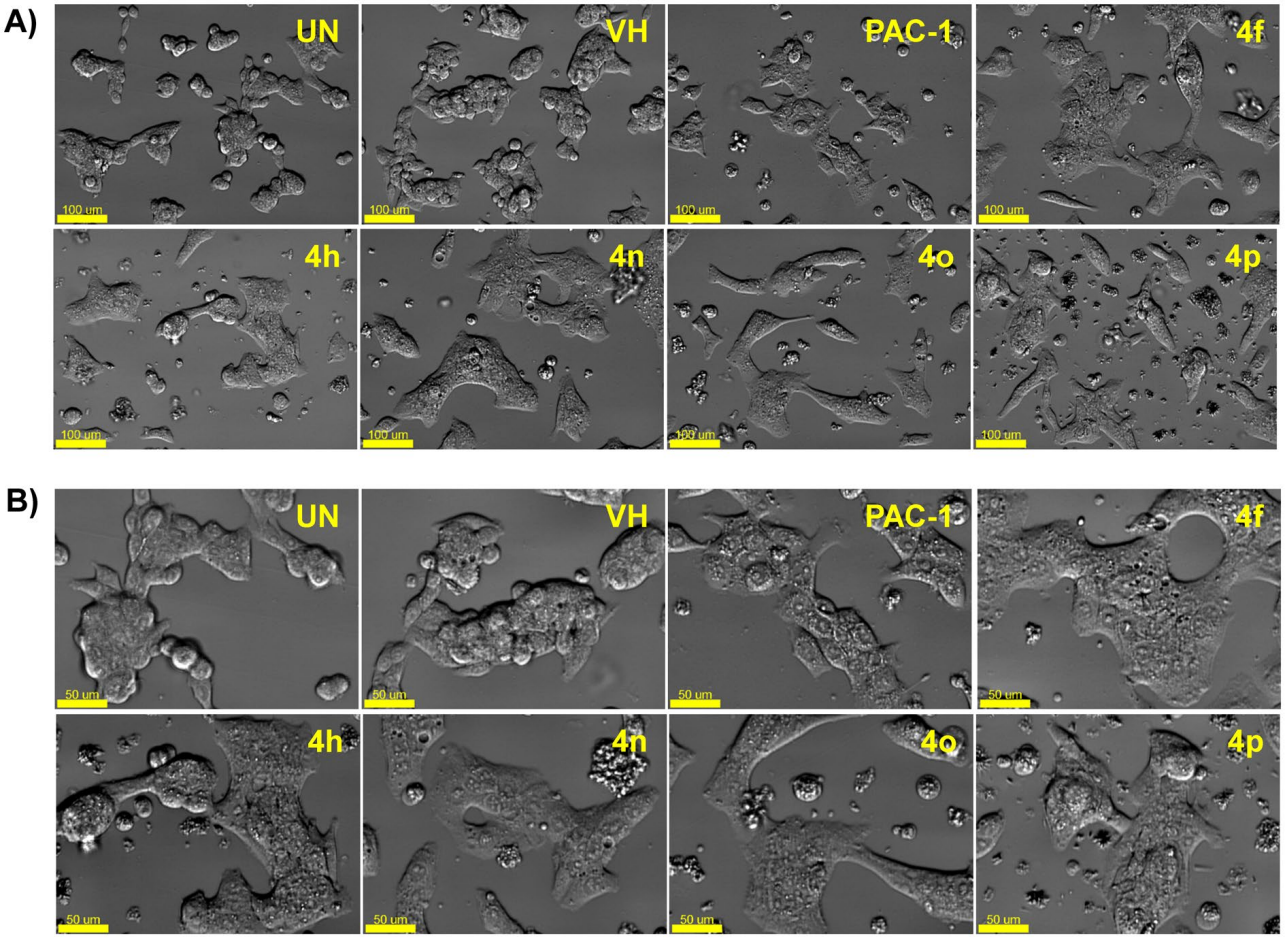
Morphology changes of cells treated with compound **4f, 4h**, **4n, 4o**, **4p** or PAC-1. SW620 at 2.5 × 10^5^ cells/mL (500 µL in 24 well) were incubated for 24 h and then further treated with compounds or PAC-1 (50 μM) for 24 h. Cells were photographed using an Imaging Device (Celldiscoverer 7) with ×20 (**A**) and ×40 (**B**) lens. Scale bar: 50 mm. *UN* untreated, *VH* vehicle (DMSO. 0.1%). Representative images are shown.

**Figure 8:**
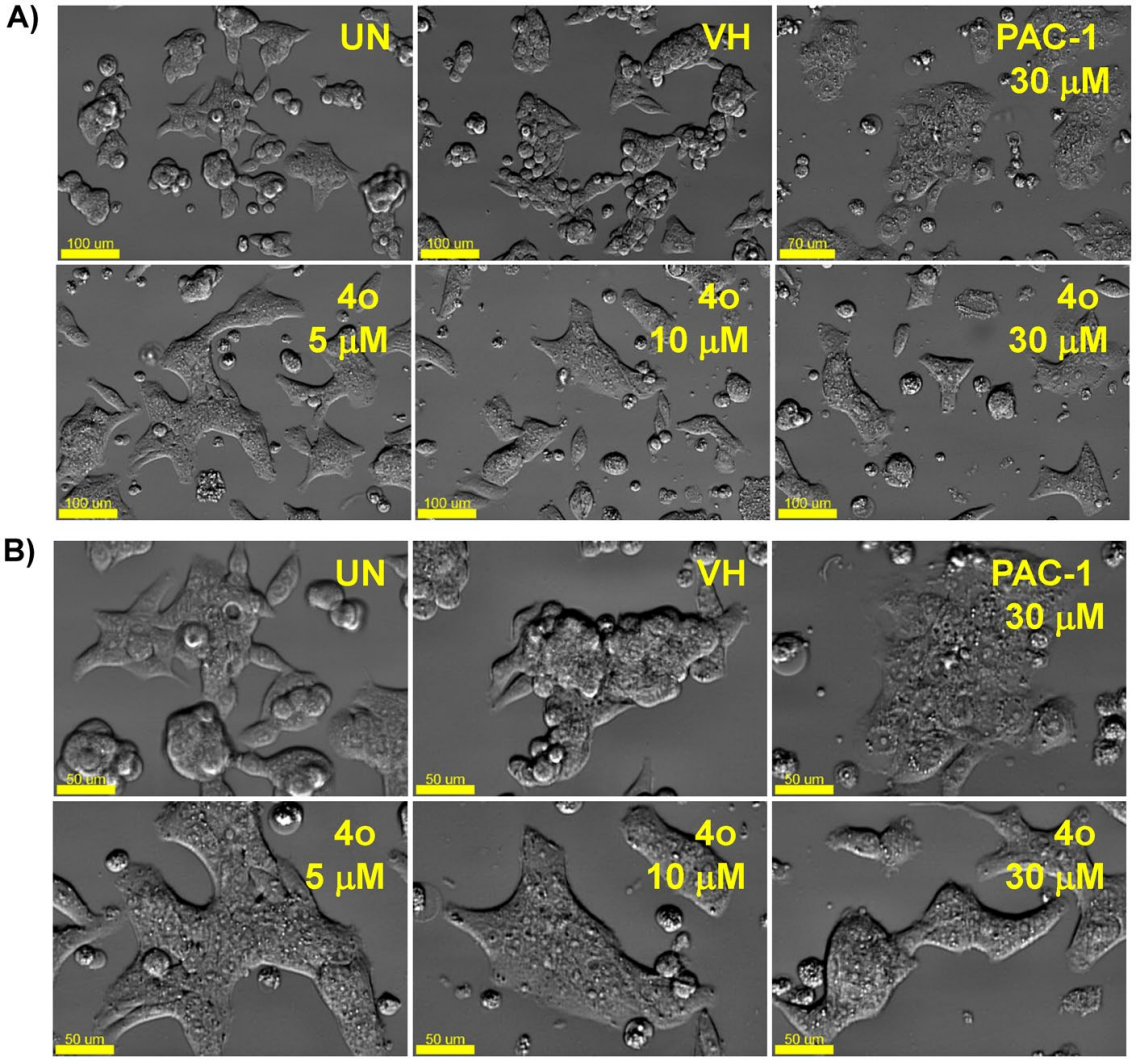
Morphology changes of cells treated with compound **4o** or PAC-1. SW620 at 2.5 × 10^5^ cells/mL (500 µL in 24 well) were incubated for 24 h and then further treated with **4o** (5, 10, and 30 μM) or PAC-1 (30 μM) for 24 h. Cells were photographed using an Imaging Device (Celldiscoverer 7) with ×20 (**A**) and ×40 (**B**) lens. Scale bar: 50 mm. *UN* untreated, *VH* vehicle (DMSO. 0.1%). Representative images are shown.

Next, we selected 5 representative compounds, including **4f, 4h, 4n, 4o** and **4p**, to investigate their effects on the caspase activity, cell cycle, and apoptosis. At first, we tried to use the extract of SW620, PC-3, and NCI-H23 in caspase activation assay, but these extracts did not show caspase activity. Thus, we used the extract of U937 cells in caspase-3 activation assay, referring to our previous study^31^. As reported, PAC-1 activated caspase-3 in 24-h-caspase-3 assay in U937 cells at 50 µM higher concentration than an IC_50_ value of 8.47 µM in 48-h-cytotoxicity assay. However, our compounds unexpectedly were not recorded to activate caspase-3 activity. It was likely that the compounds might activate other caspases and eventually caused effects on the cell cycle and apoptosis. Next, we investigated the effect of PAC-1 and our chemicals on cell cycle and apoptosis by using U937 cells^31^. In the cell cycle analysis, U937 cells were treated with 50 µM of compounds for 24 h, stained with propidium iodide (PI) in the presence of RNase, and then analyzed for DNA content by using flow cytometry. PAC-1 was used in parallel as a positive control. The results illustrated in Fig. 3 indicate that the compounds tended to cause accumulation of cells in S phase, although PAC-1 caused the accumulation of cells in G0/G1 phase. Compound **4o** inhibited cell cycles at 5 µM, which was close to IC_50_ value (Fig. 4). In the Annexin V-FITC/PI apoptotic analysis, compounds **4f, 4h, 4n, 4o** and **4p** also induced early, and more substantially, late apoptosis (Fig. 5). The effects were more prominent with compounds **4h, 4o** and **4p** (Fig. 5). Compound **4o** increased late apoptotic cell population at 5 µM, which was close to IC_50_ value (Fig. 6). Regarding the effects of the compounds on cellular morphology, SW620 cells treated with PAC-1 and our compounds showed morphology of apoptotic cells (Figs. 7 and 8).

## Conclusions

In conclusion, two series of novel (*E*)-*N'*-arylidene-2-(2-oxoindolin-1-yl)acetohydrazides (**4a–p)** and *(Z)-*2-(5-substituted-2-oxoindolin-1-yl)-*N'*-(2-oxoindolin-3-ylidene)acetohydrazides (**5a–f)** were designed and synthesized. Biological results revealed that the significant cytotoxicity against three human cancer cell lines SW620, PC-3, and NCI-H23 of these compounds were obtained. Under our conditions, compounds **4f–h** and **4n–p**, exhibited cytotoxicity equal to superior to positive control PAC-1. In particular, compound **4o** was the most potent with cytotoxicity up to three- to five-fold stronger than PAC-1 in three cancer cell lines tested. Cell cycle and apoptosis analysis showed that representative compounds **4f, 4h, 4n, 4o** and **4p** (especially **4o**) accumulated U937 cells in the S phase and substantially induced late cell apoptosis. Collectively, the results show that compound **4o** would serve as a template for further design and development of novel anticancer agents.

## Supplementary Information

Supplementary Figures.
