# Supplementary material for: Design, synthesis and evaluation of novel 2-oxoindoline-based acetohydrazides as antitumor agents
Source: Sci Rep. 2022 Feb 21;12:2886. doi: 10.1038/s41598-022-06887-0 (PMC8861050; doi:10.1038/s41598-022-06887-0)

SUPPLEMENTAL MATERIALS
for
Design, Synthesis and Evaluation of Novel 2-Oxoindoline-based Acetohydrazides as Antitumor Agents

Do T. M. Dung,[#, a] Eun J. Park,[#, b] Duong T. Anh,[a] Dung T. P. Phan,[a] Ik H. Na,[b] Joo H. Kwon,[c] Jong S. Kang,[c] Truong T. Tung,[d,e] Sang-Bae Han,[*,b] Nguyen-Hai Nam[*,a]

[a]Hanoi University of Pharmacy, 13-15 Le Thanh Tong, Hanoi, Vietnam

[b]College of Pharmacy, Chungbuk National University, 194-31, Osongsaengmyung-1, Heungdeok, Cheongju, Chungbuk, 28160, Republic of Korea

[c]Korea Research Institute of Bioscience and Biotechnology, Cheongju, Chungbuk, Republic of Korea

[d]Faculty of Pharmacy, PHENIKAA University, Hanoi, 12116, Vietnam;

[e]PHENIKAA Institute for Advanced Study (PIAS), PHENIKAA University, Hanoi, 12116, Vietnam

[#]These authors contributed equally to this work.
*Corresponding authors: SB Han (shan@chungbuk.ac.kr), NH Nam (doctornam@gmail.com)

Copy of HRMS, ^{1}H & ^{13}C NMR SPECTRA OF THE COMPOUNDS

Figure S1. HRMS of compound 4a

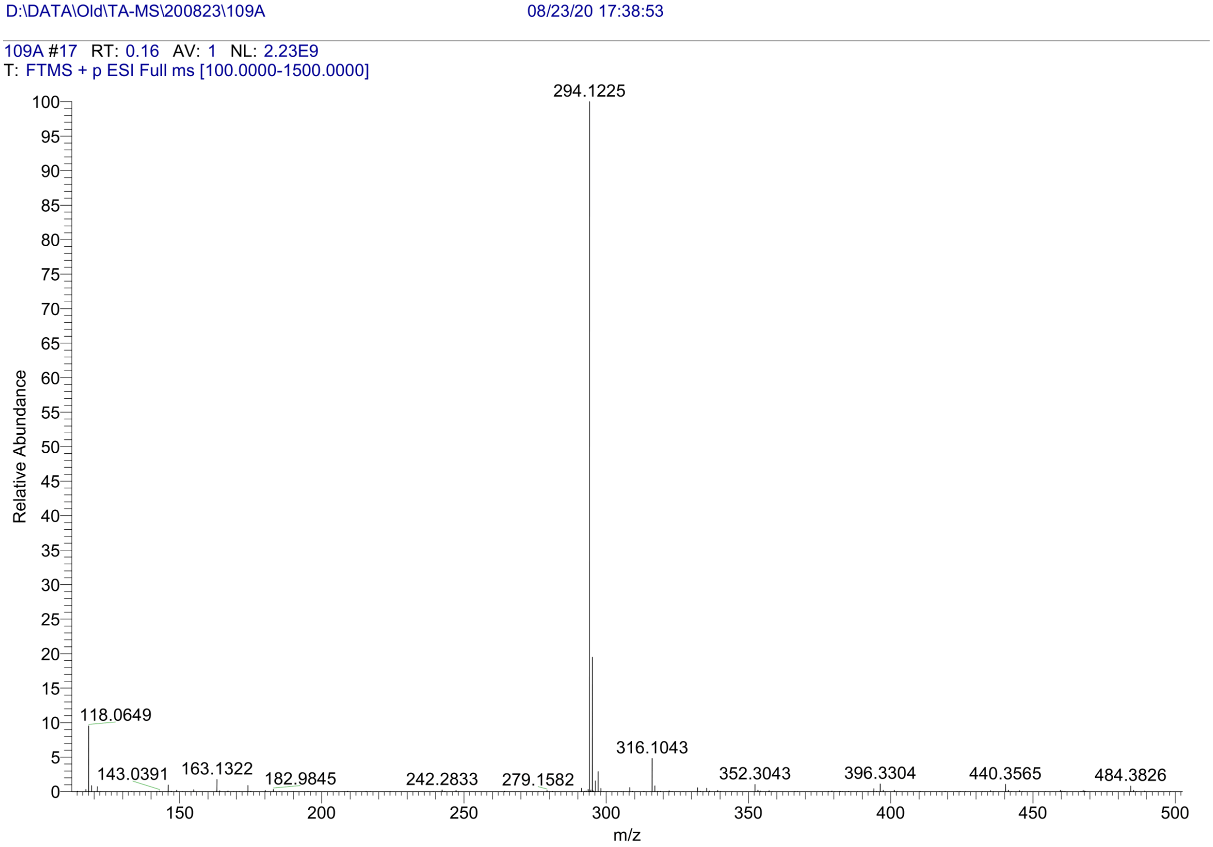

Figure S2. ^{1}H NMR of compound 4a

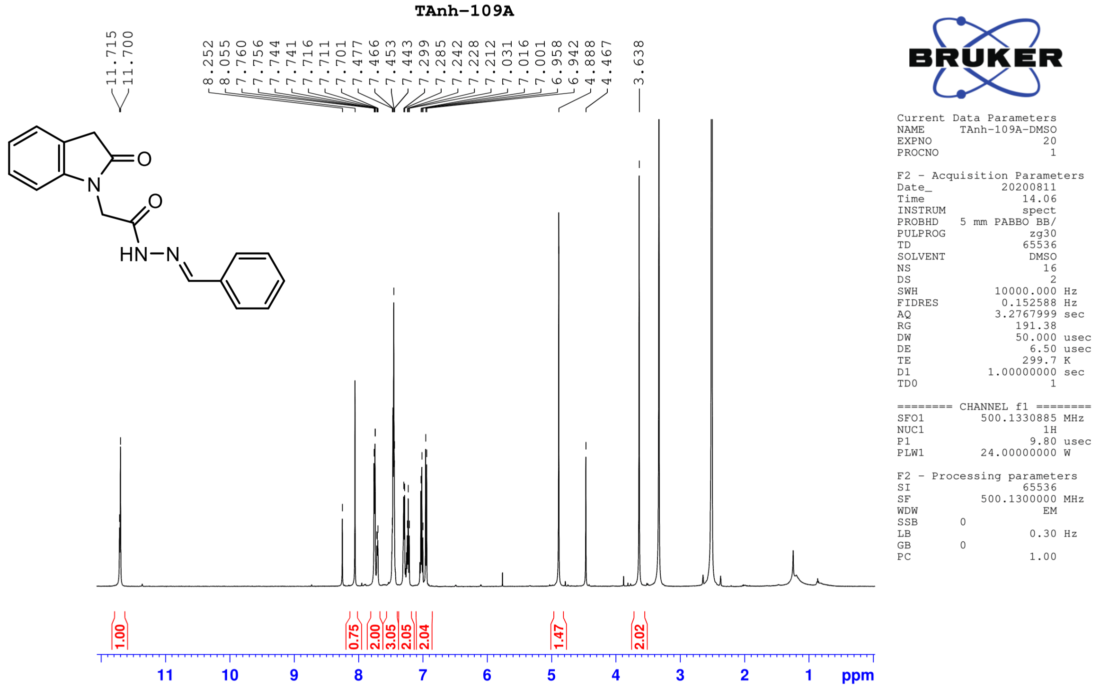

Copy of HRMS, ^{1}H & ^{13}C NMR SPECTRA OF THE COMPOUNDS

Figure S1. HRMS of compound 4a

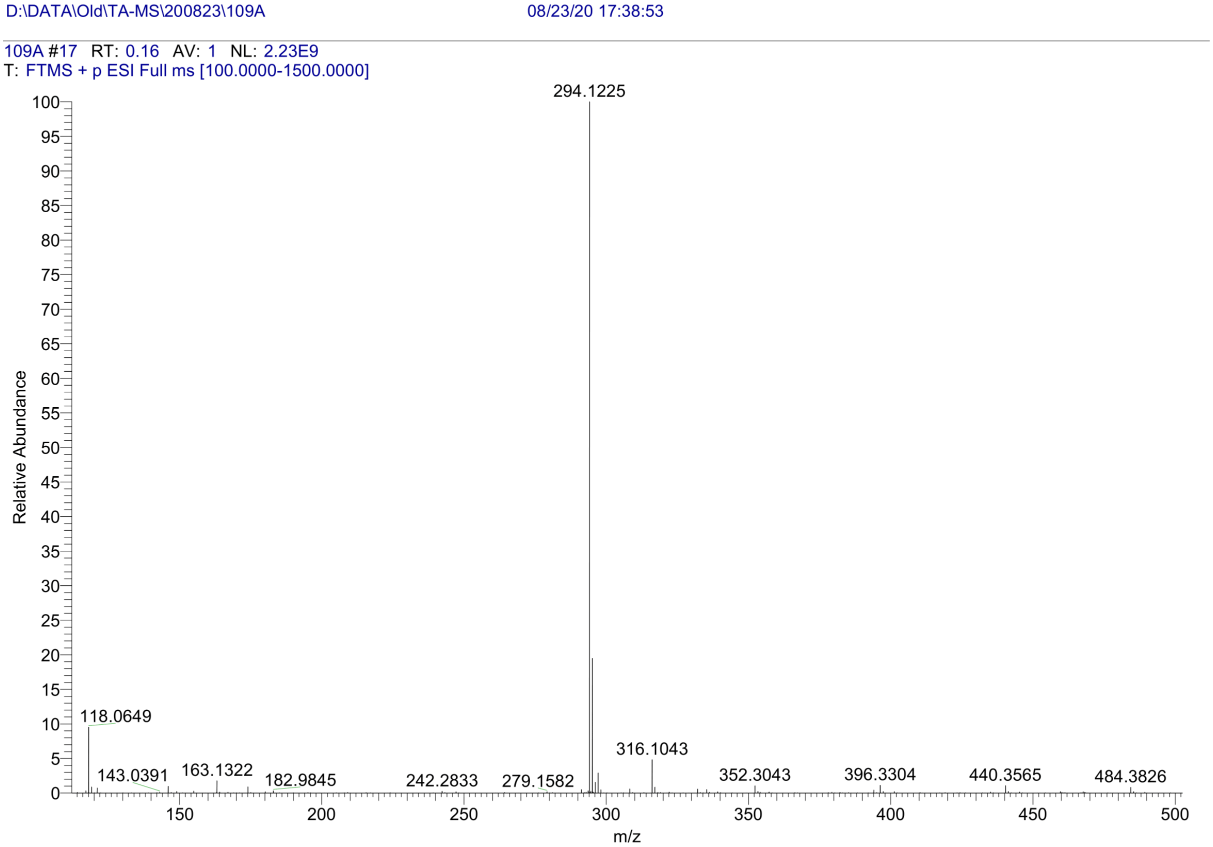

Figure S2. ^{1}H NMR of compound 4a

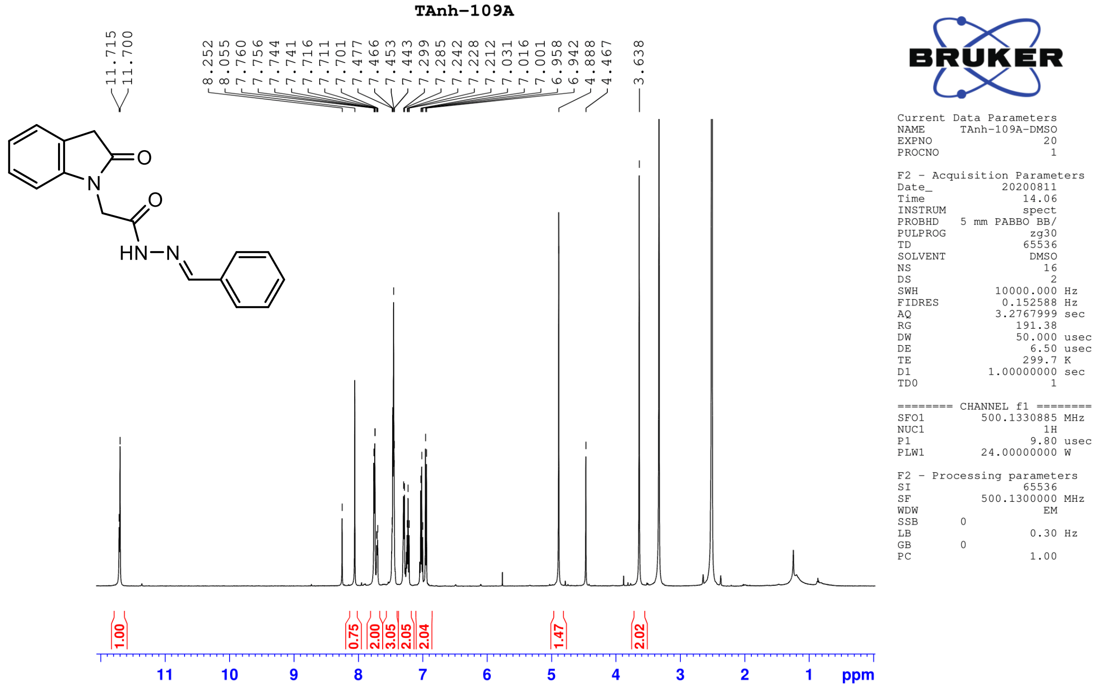

Figure S3. ^{13}C NMR of compound 4a

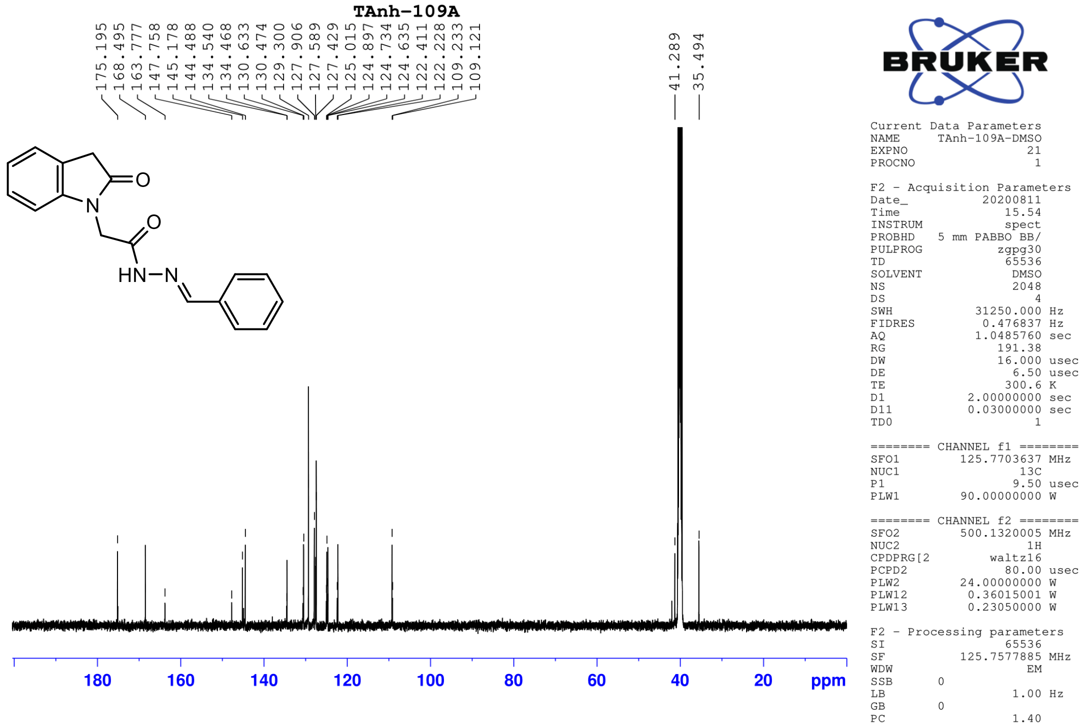

Figure S4. HRMS of compound 4b

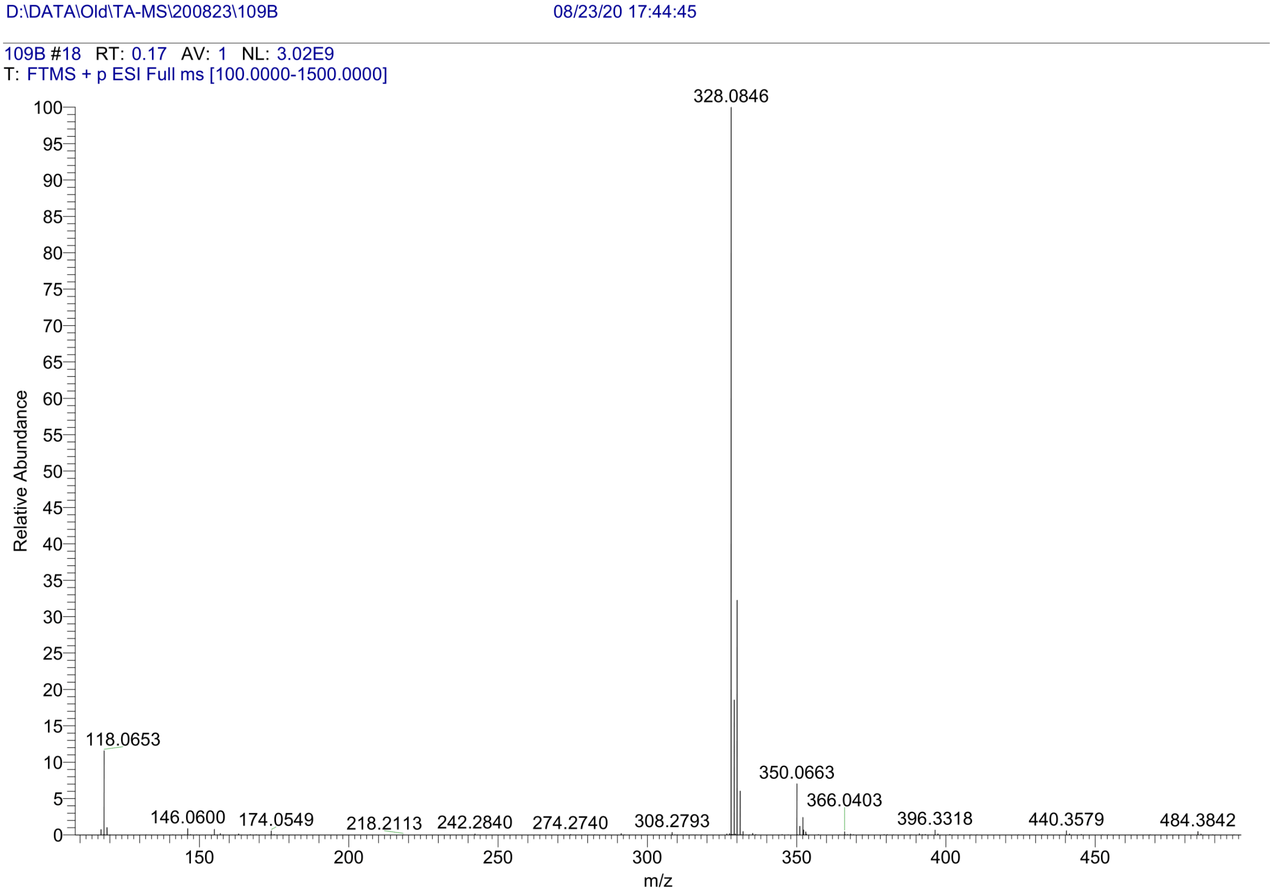

Figure S5. ^{1}H NMR of compound 4b

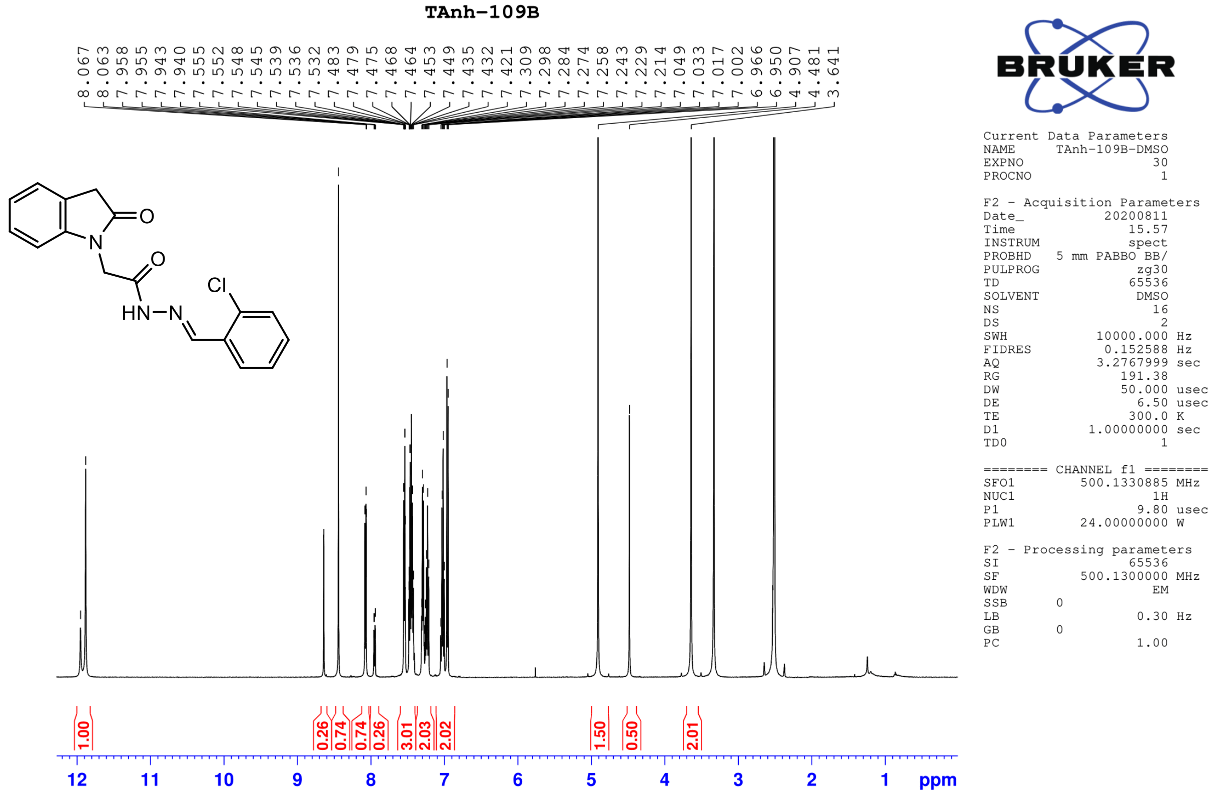

Figure S6. ^{13}C NMR of compound 4b

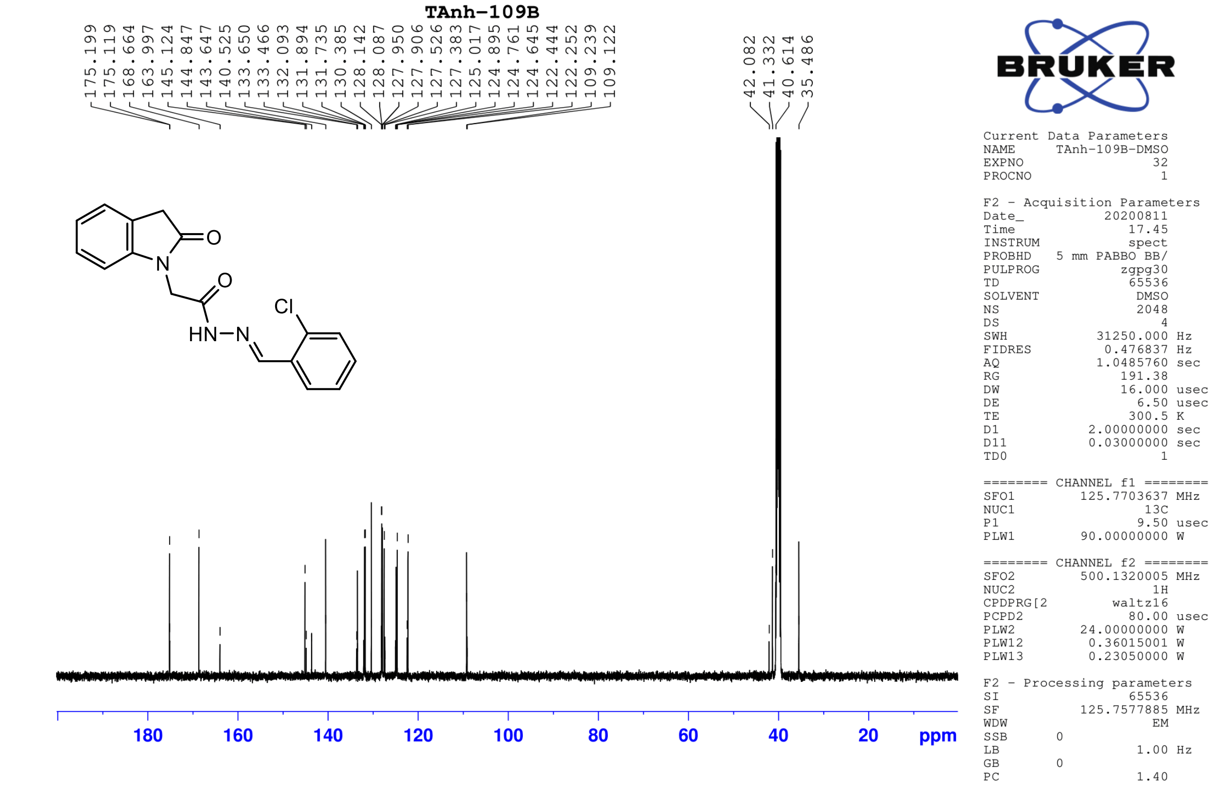

Figure S7. HRMS of compound 4c

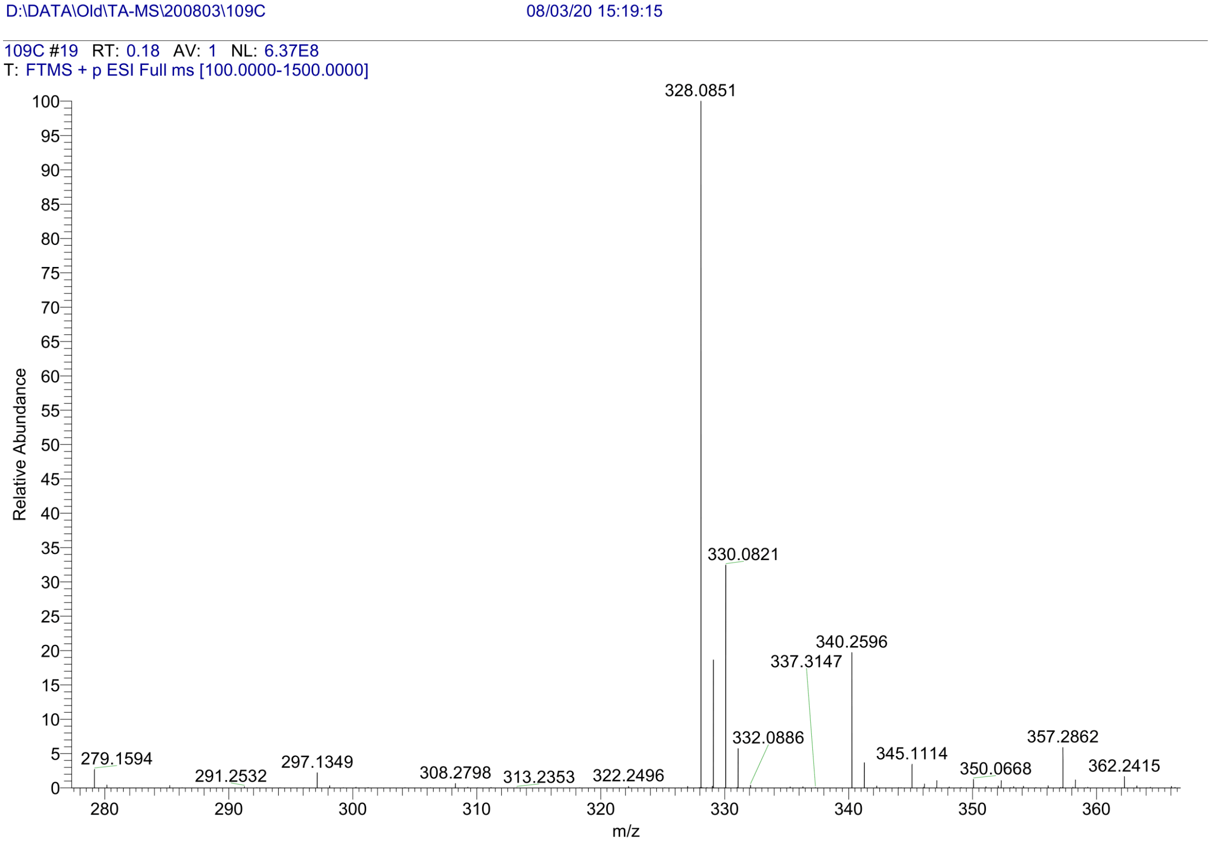

Figure S8. ^{1}H NMR of compound 4c

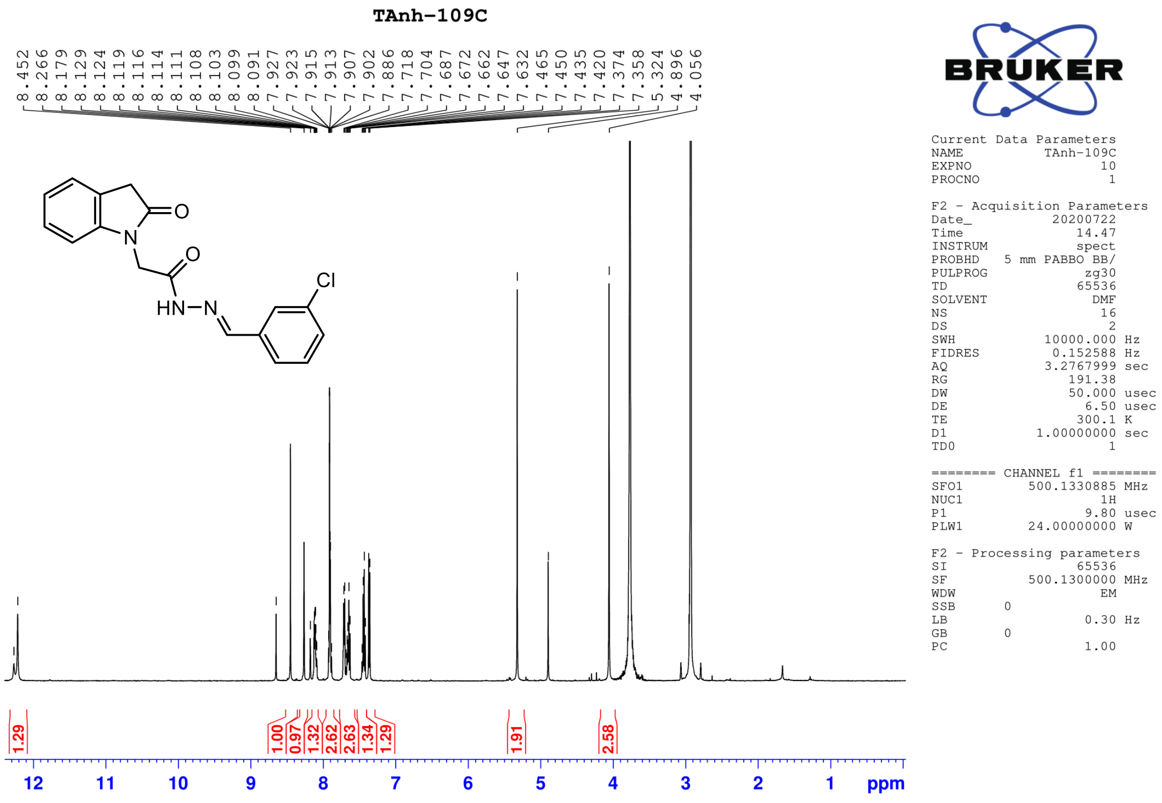

Figure S9. ^{13}C NMR of compound 4c

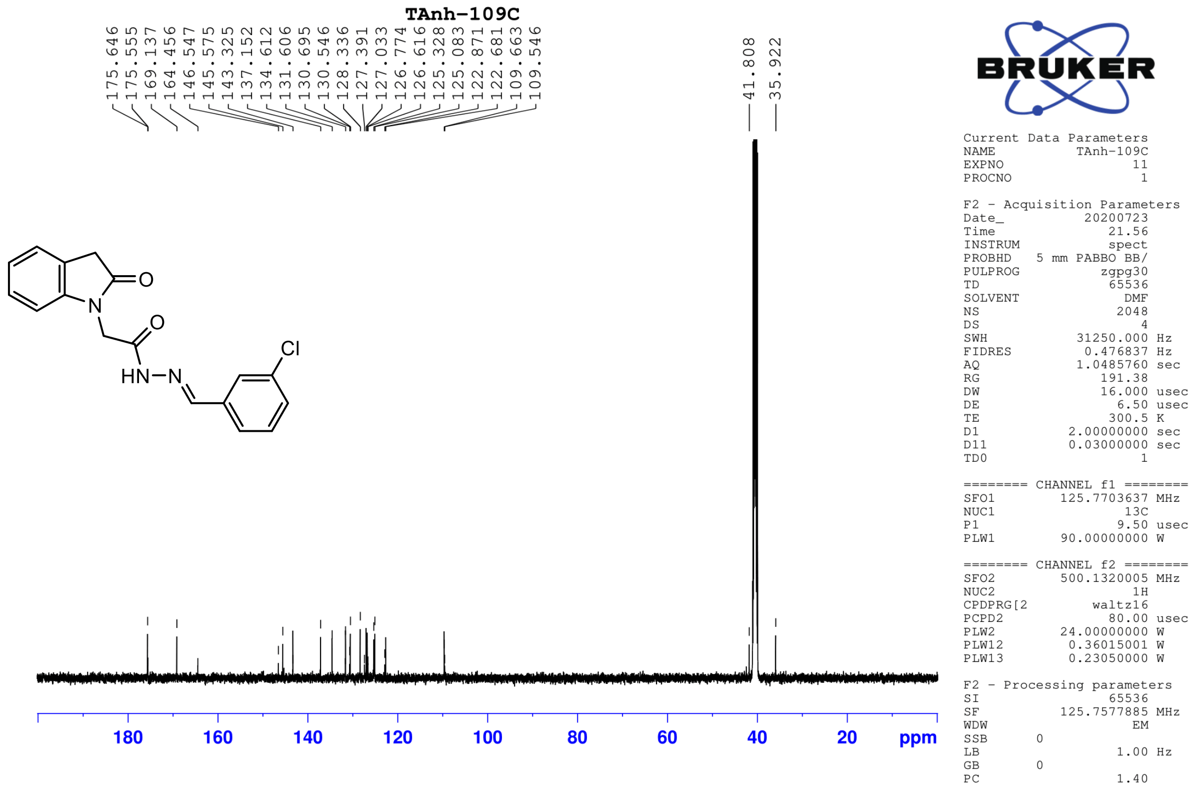

Figure S10. HRMS of compound 4d

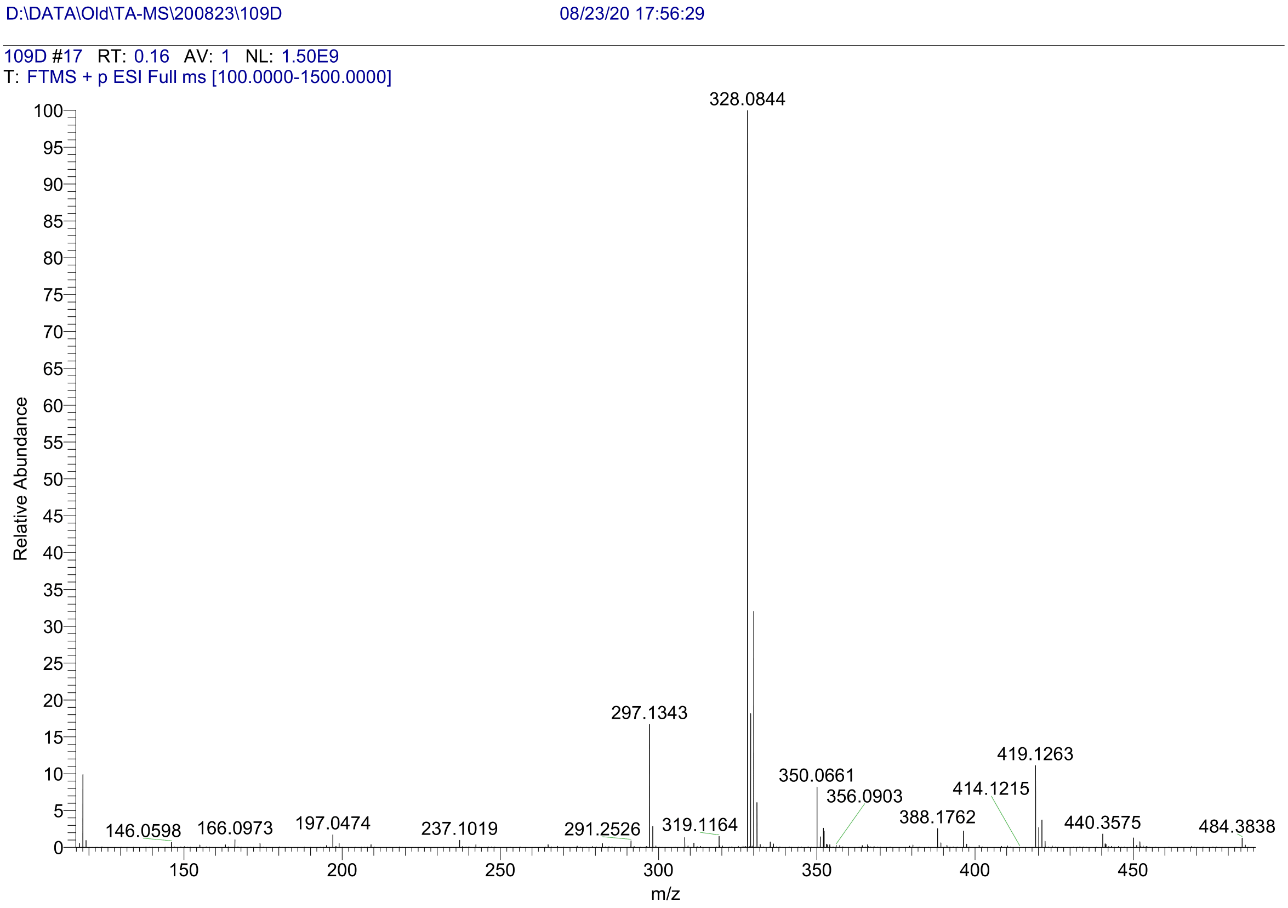

Figure S11. ^{1}H **NMR of compound 4d**

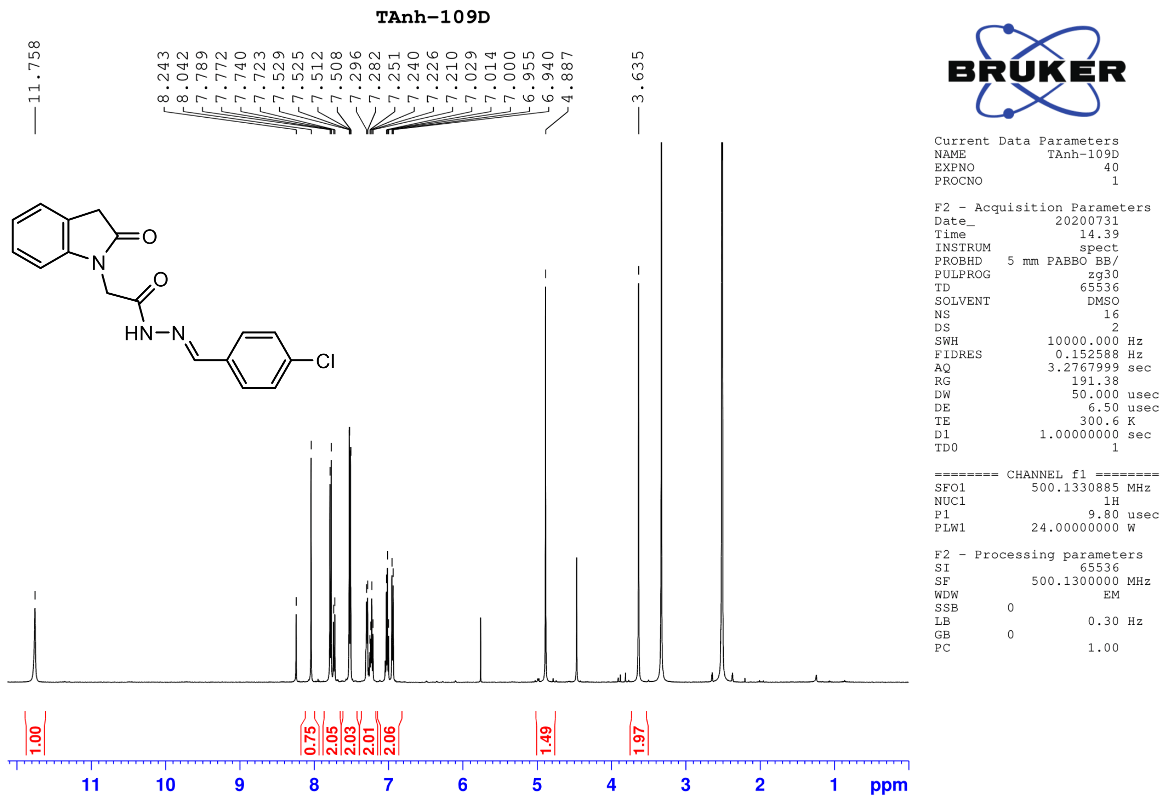

Figure S12. ^{13}C **NMR of compound 4d**

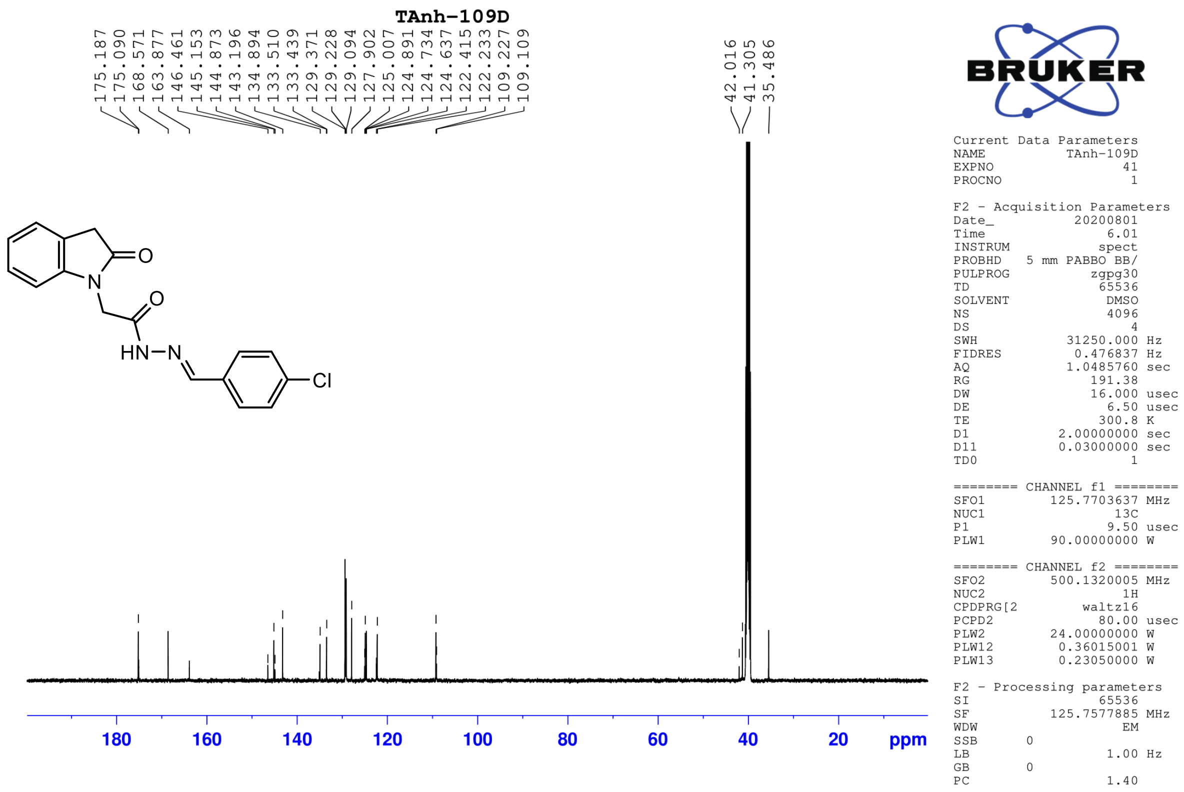

Figure S13. HRMS of compound 4e

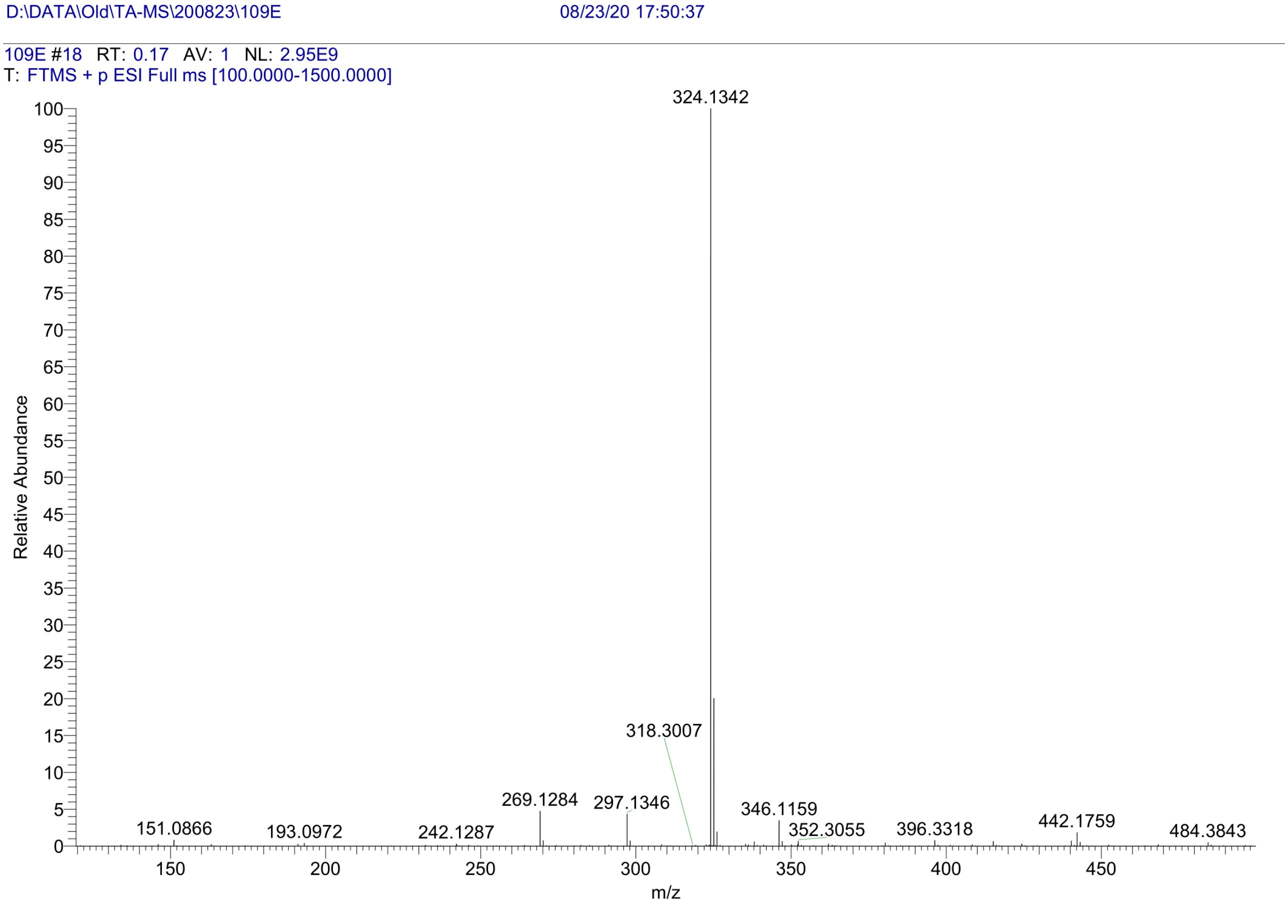

Figure S14. ^{1}H NMR of compound 4e

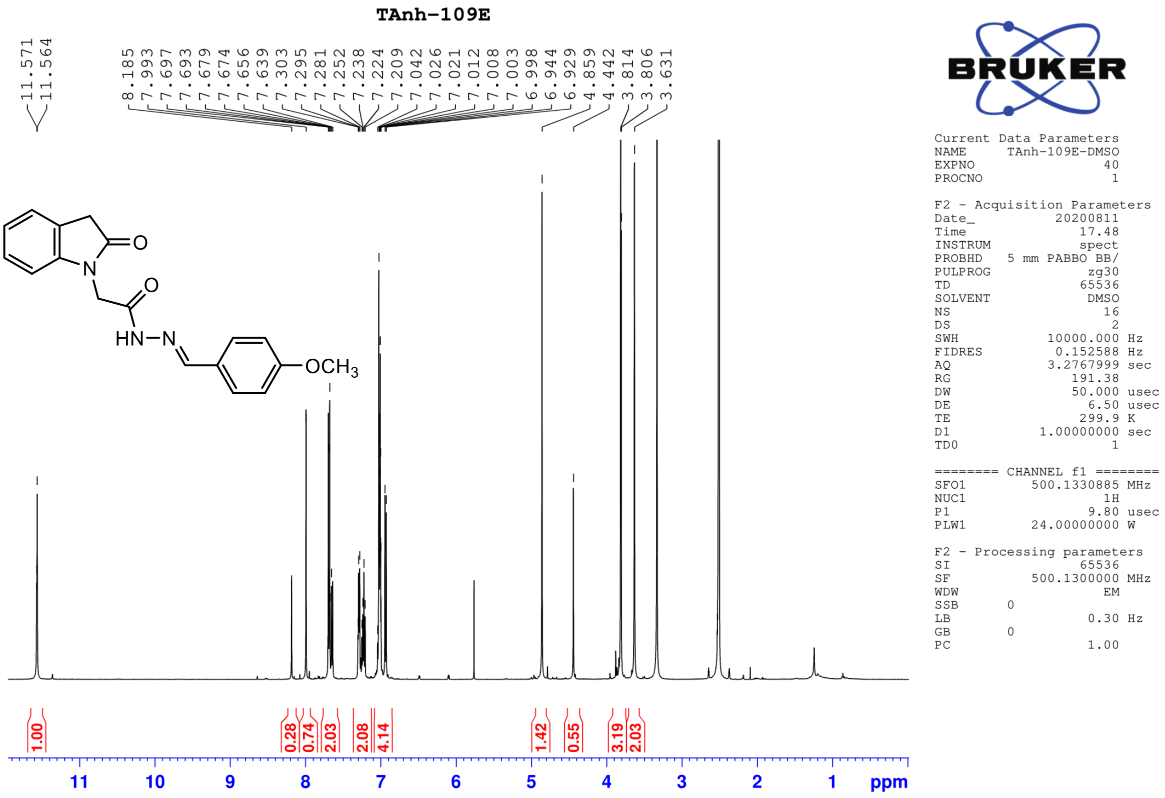

Figure S15. ^{13}C NMR of compound 4e

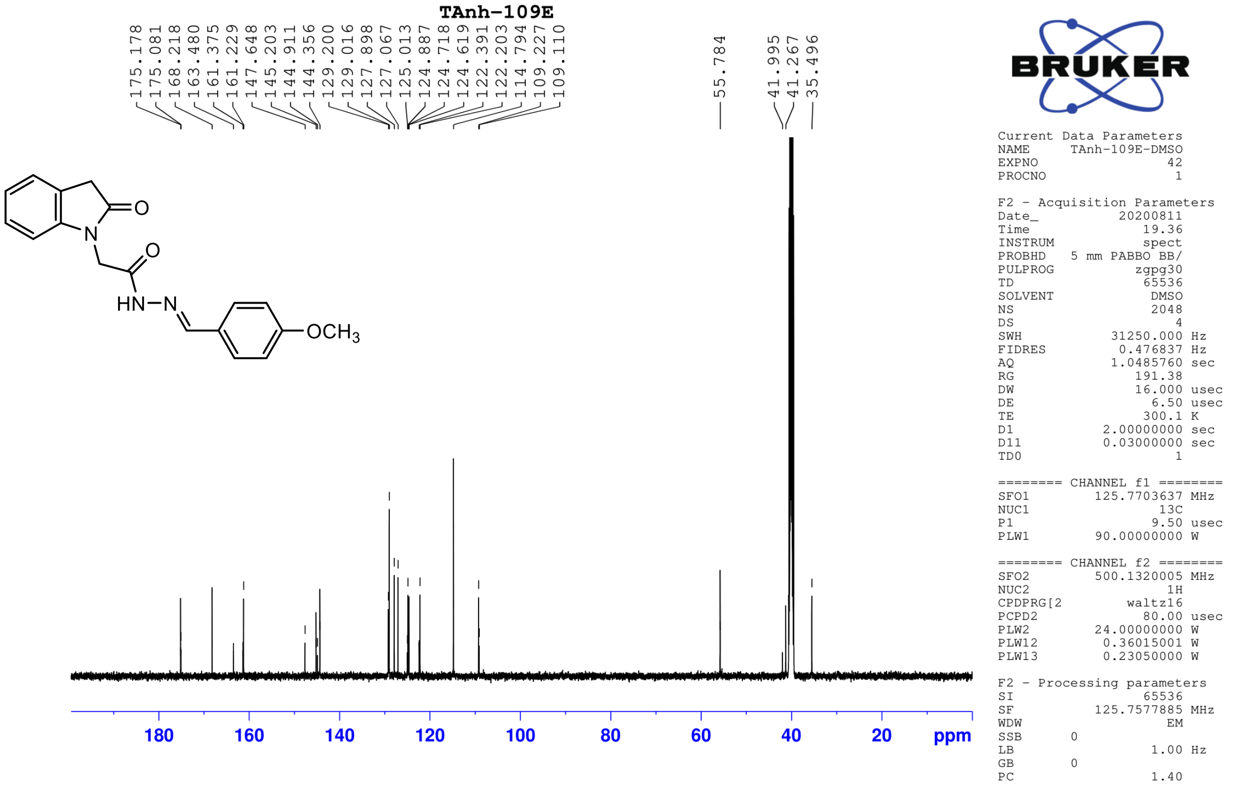

Figure S16. HRMS of compound 4f

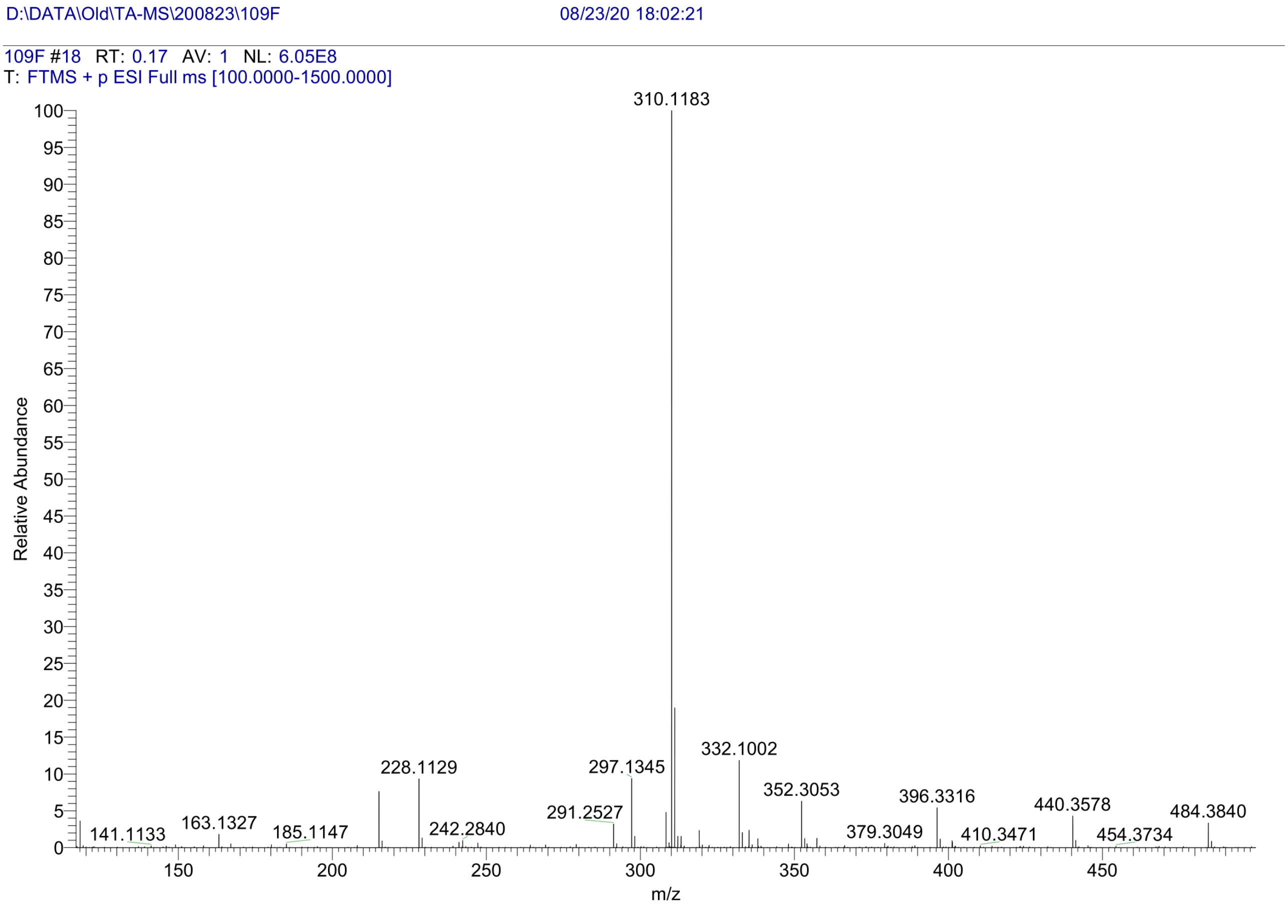

Figure S17. ^{1}**H NMR of compound 4f**

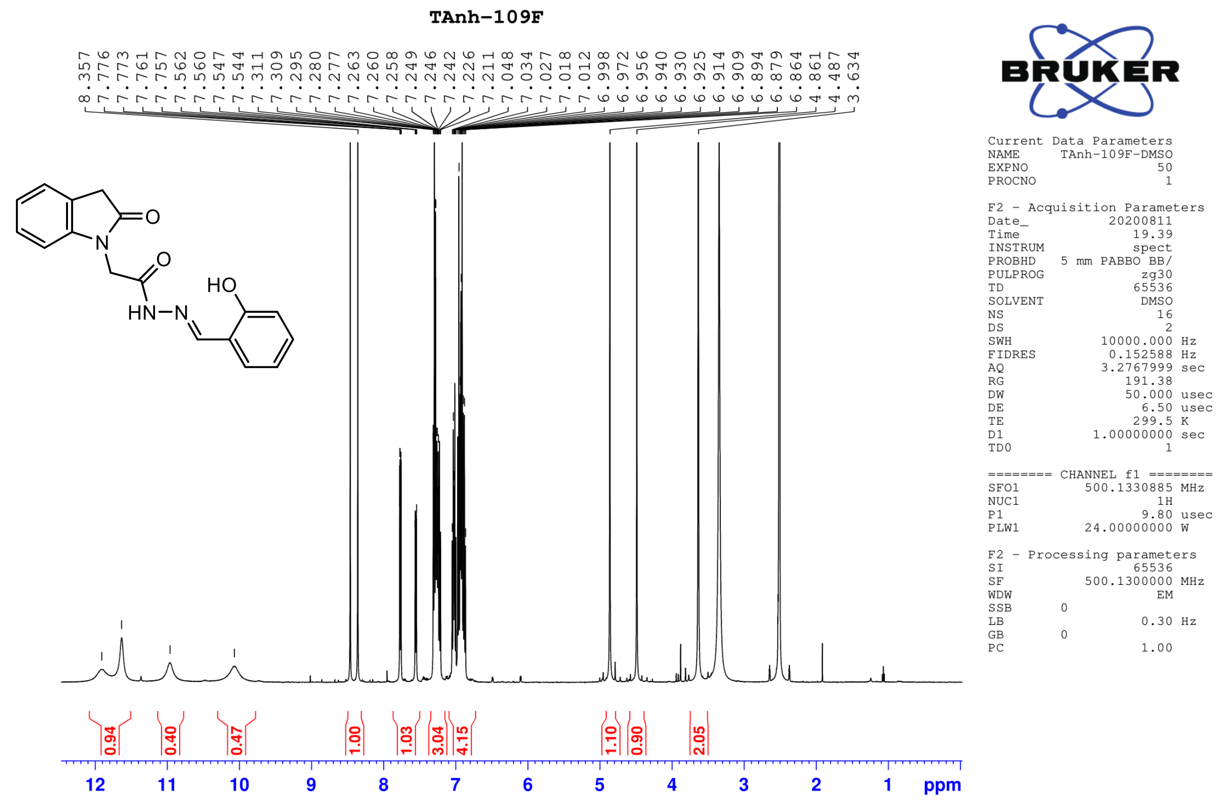

Figure S18. ^{13}**C NMR of compound 4f**

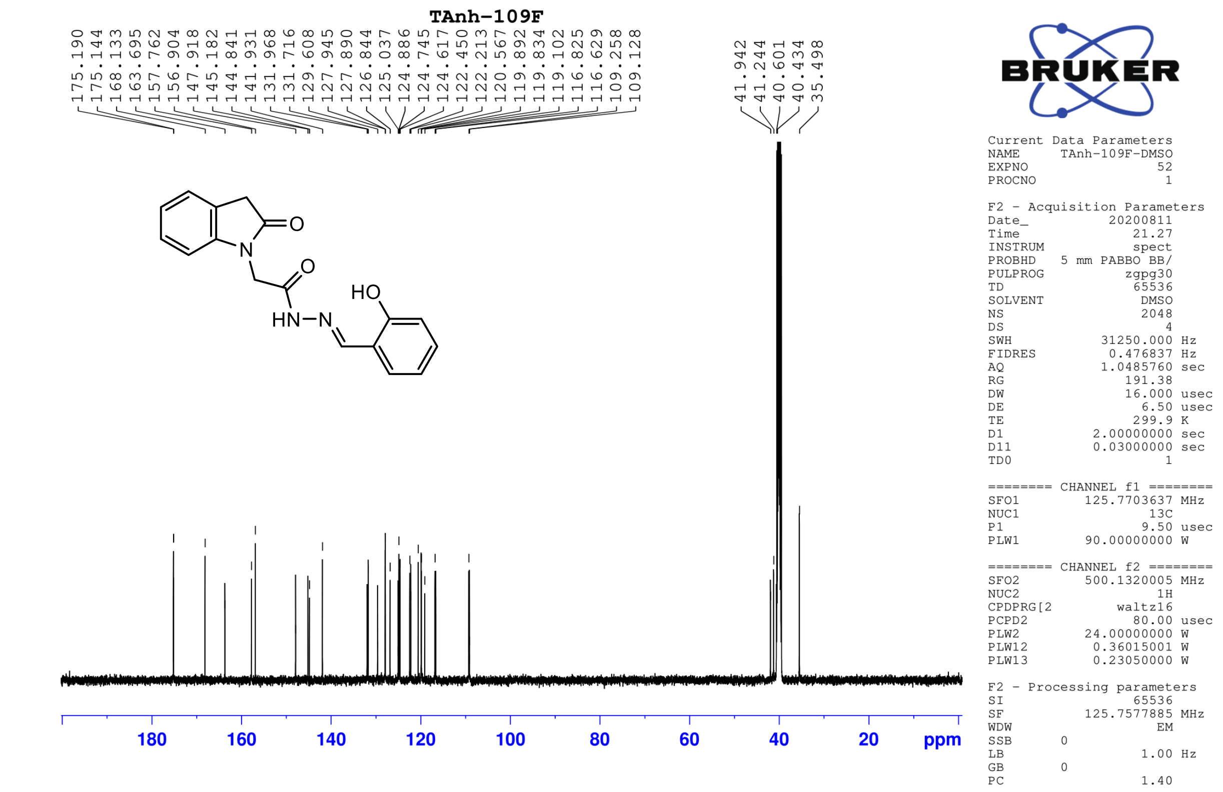

Figure S19. HRMS of compound 4g

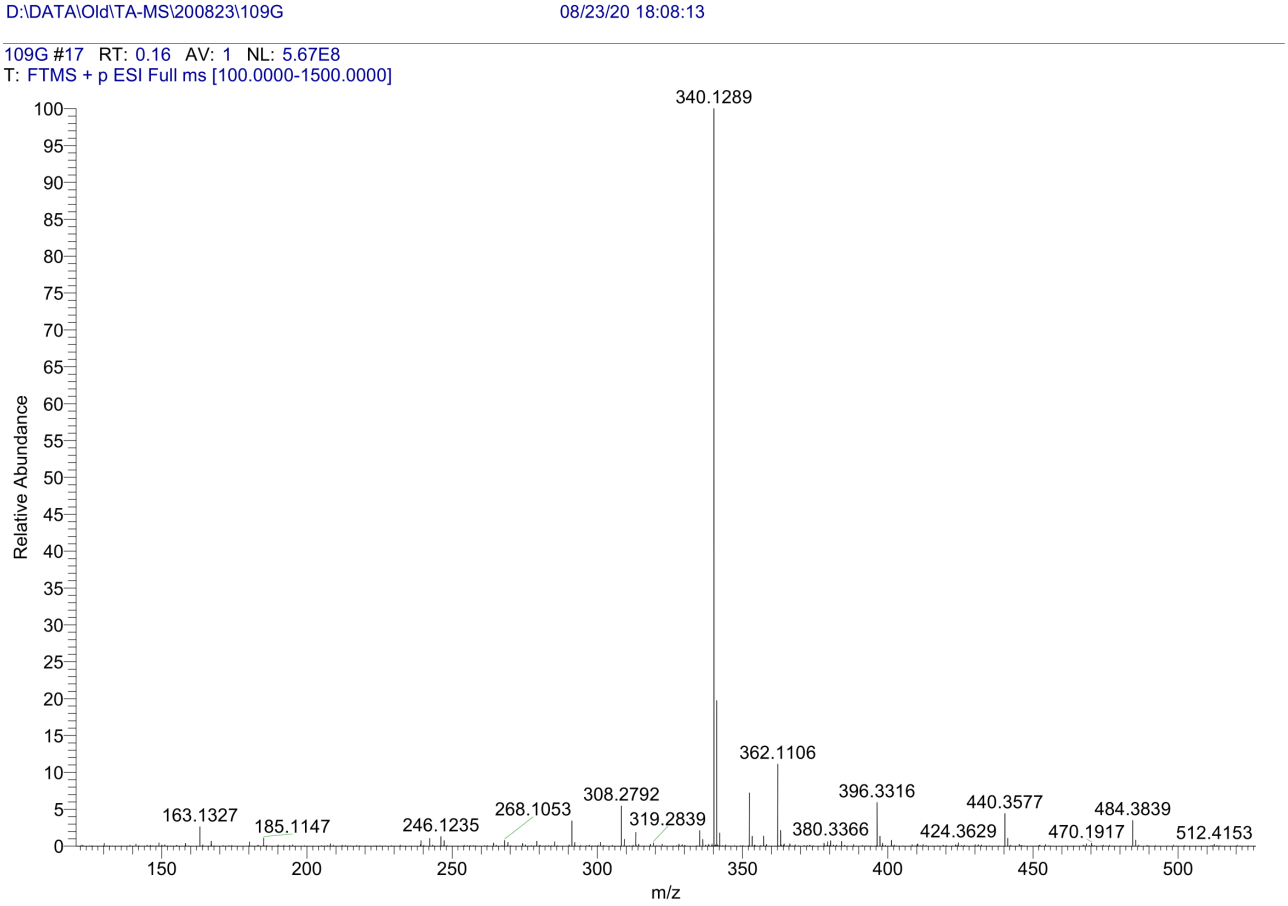

Figure S20. ^{1}H NMR of compound 4g

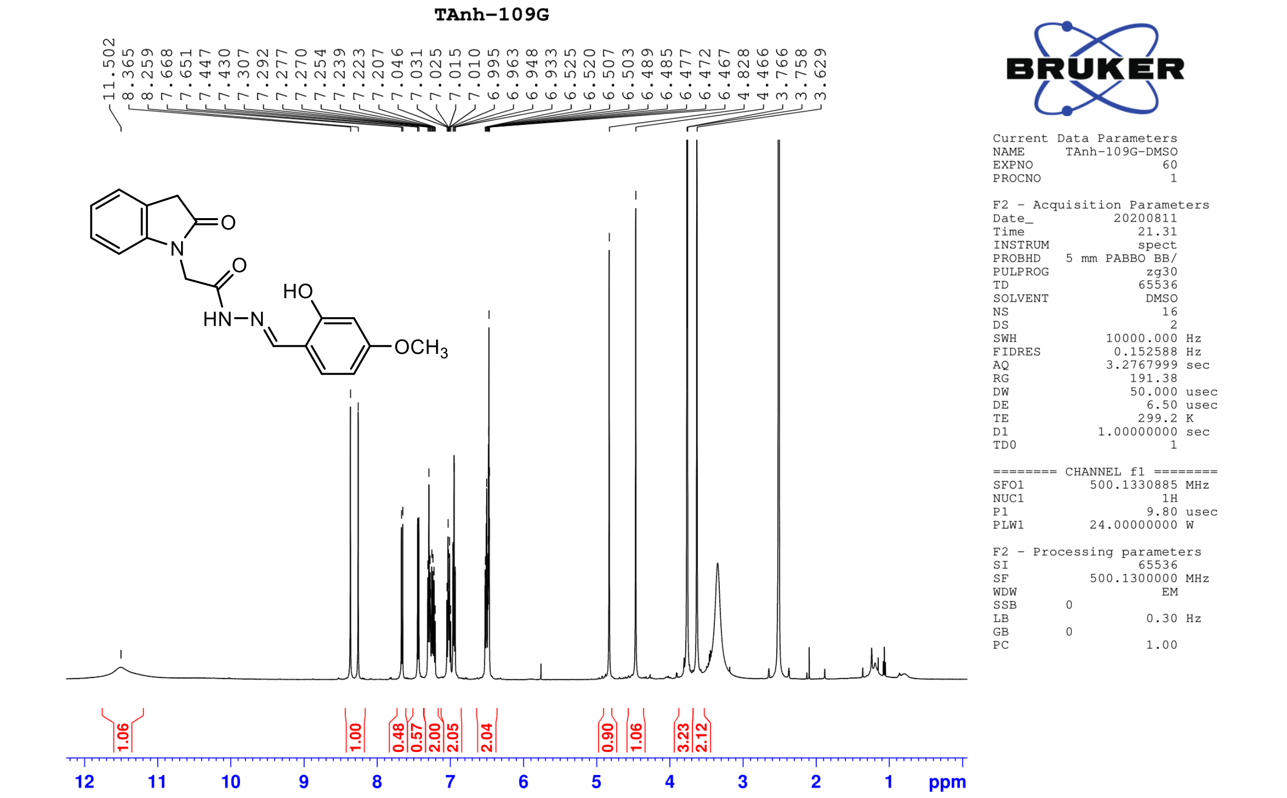

Figure S21. ^{13}C NMR of compound 4g

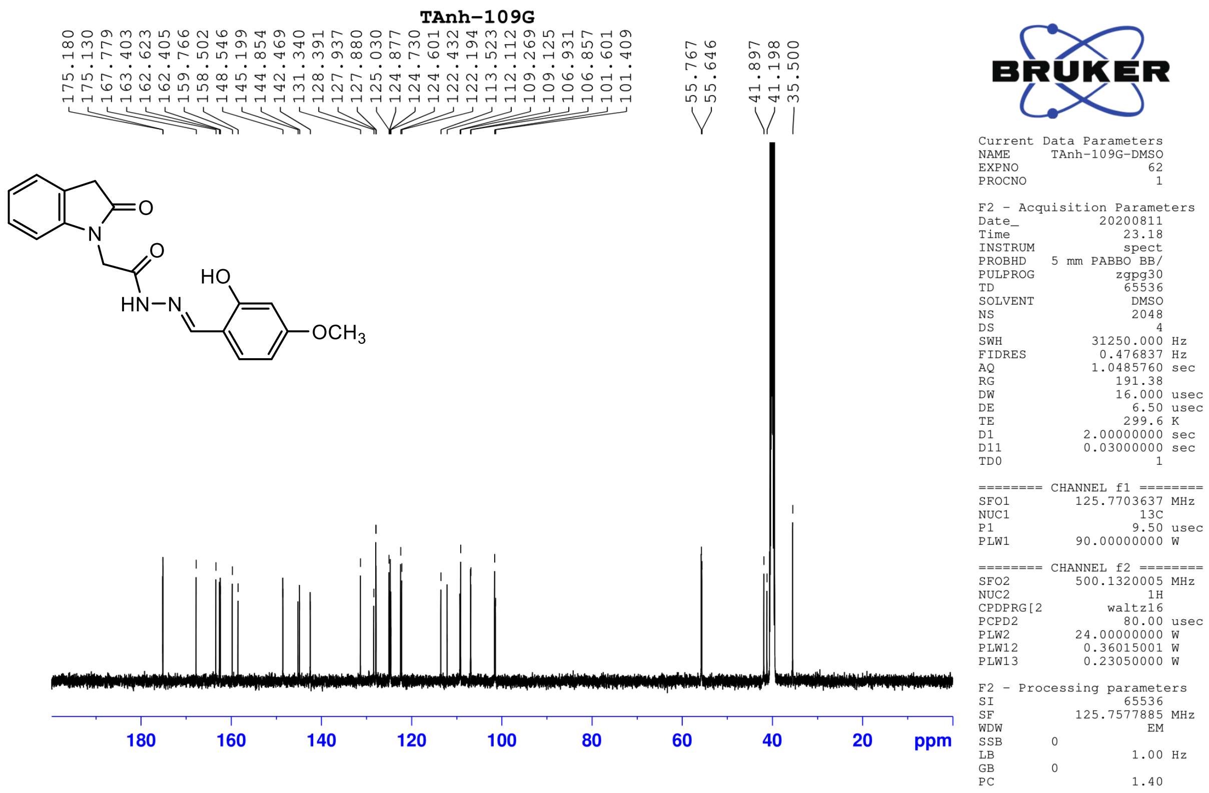

Figure S22. HRMS of compound 4h

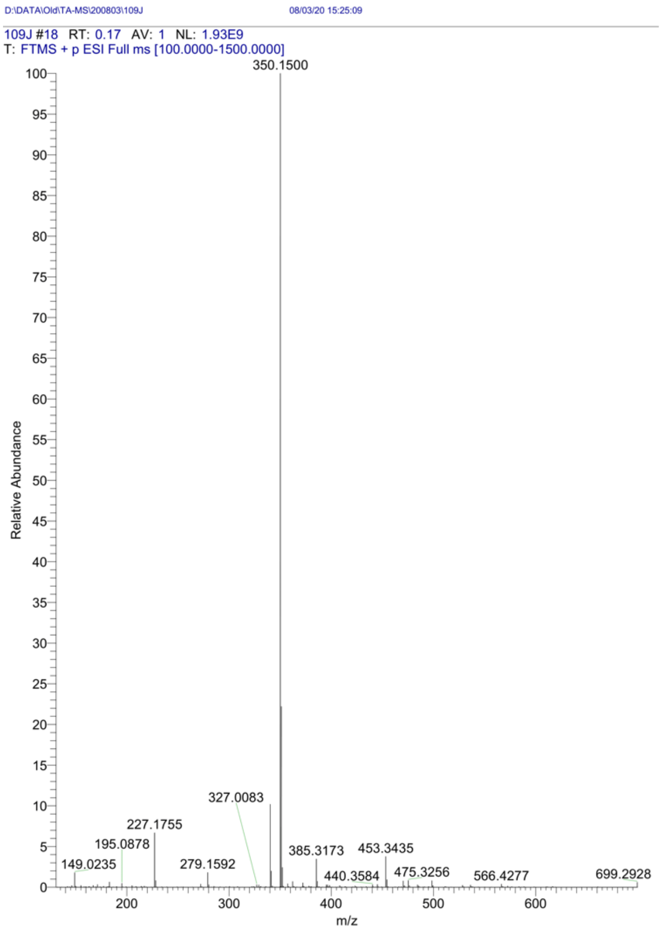

Figure S23. ^{1}**H NMR of compound 4h**

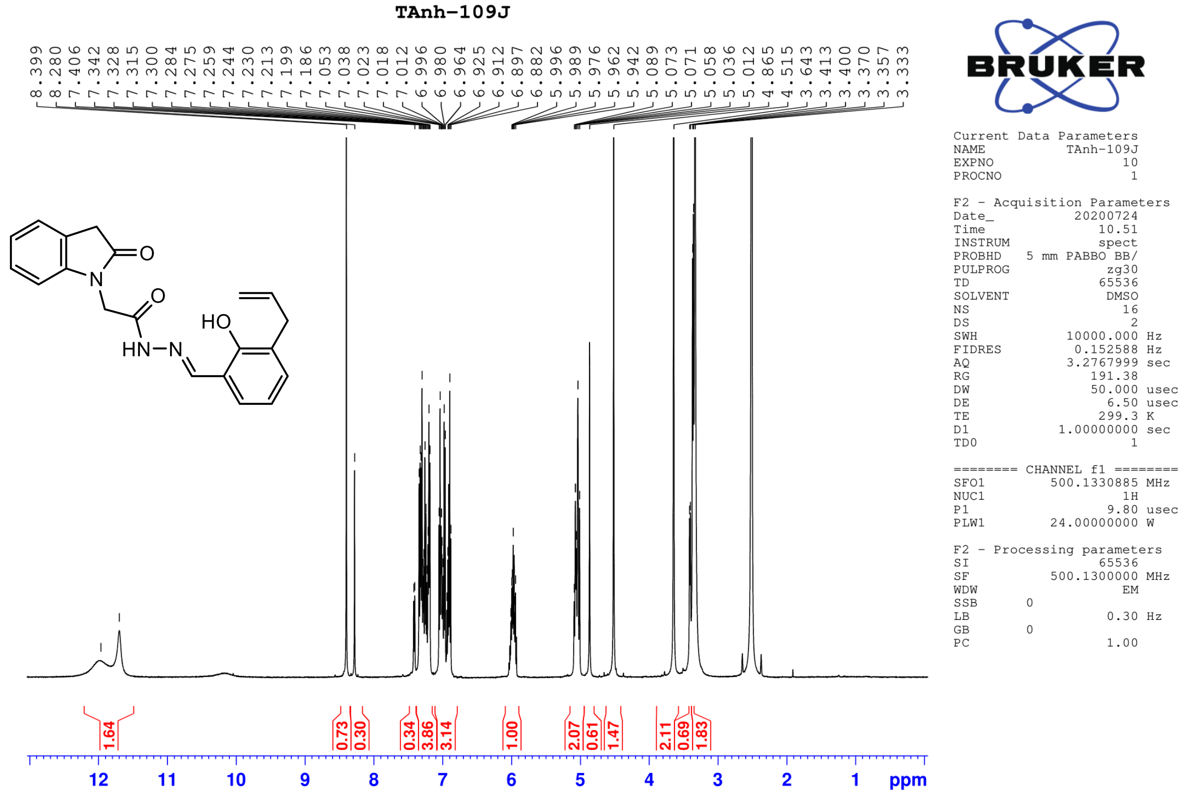

Figure S24. ^{13}**C NMR of compound 4h**

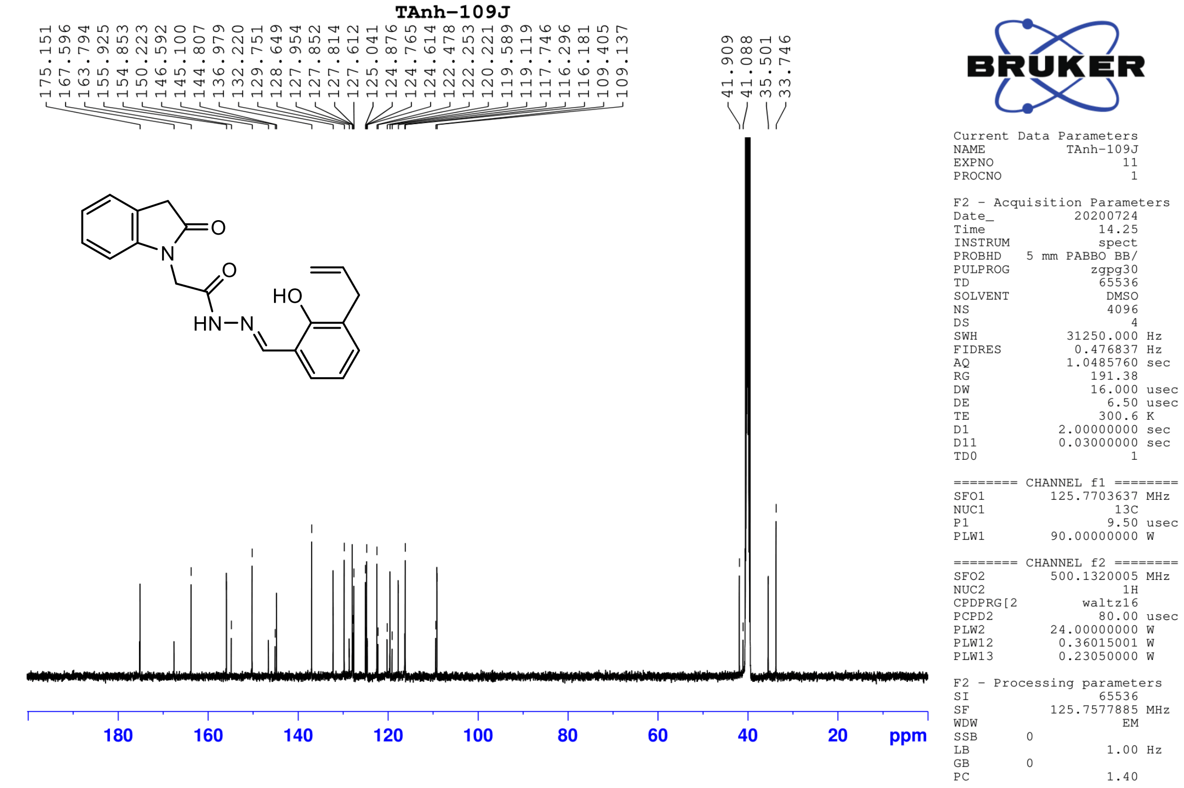

Figure S25. HRMS of compound 4i

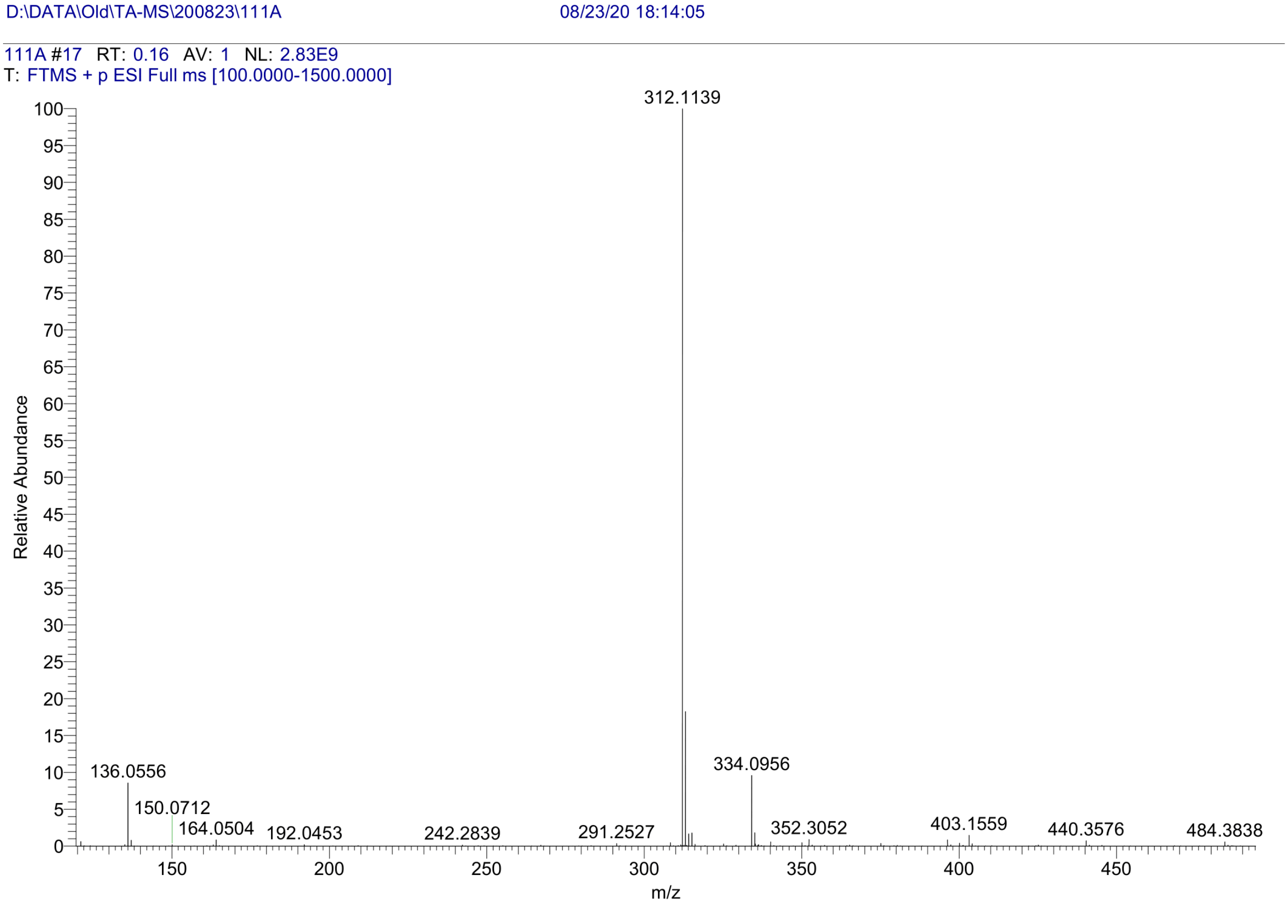

Figure S26. ^{1}H NMR of compound 4i

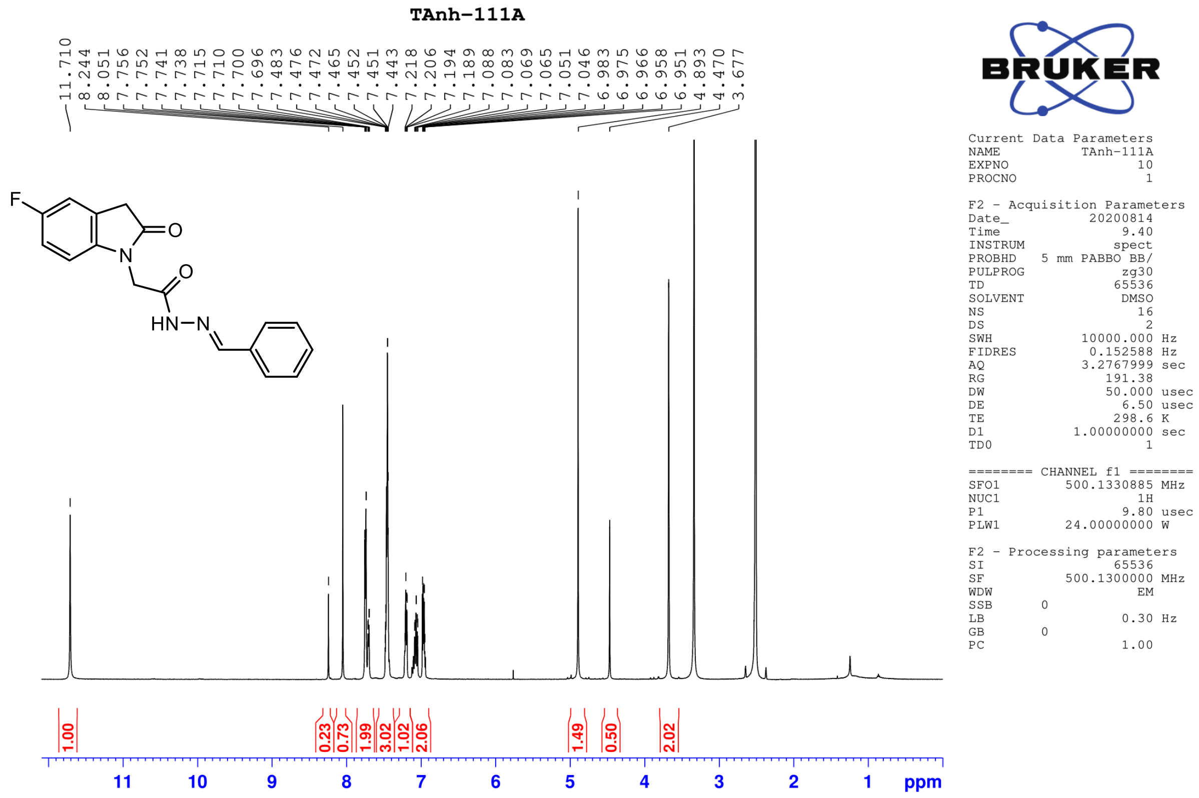

Figure S27. ^{13}C NMR of compound 4i

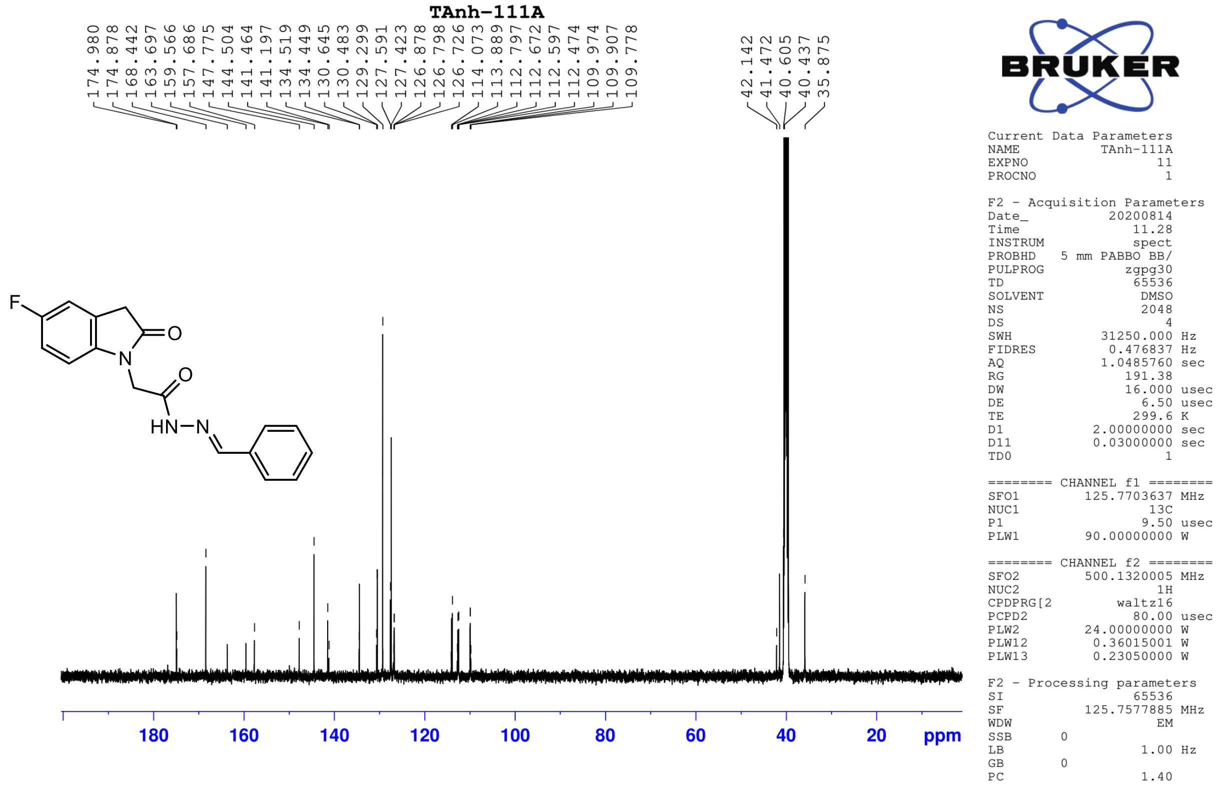

Figure S28. HRMS of compound 4j

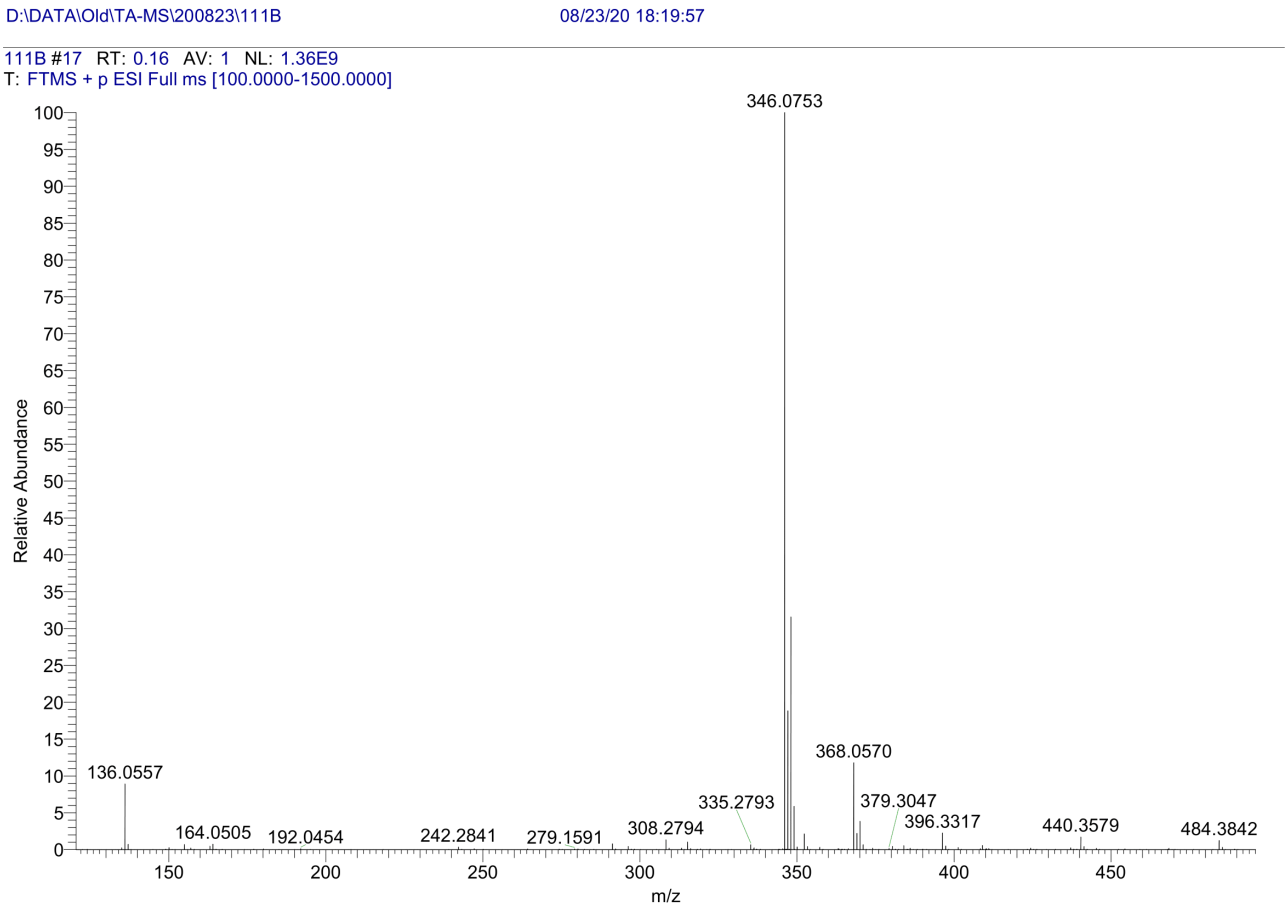

Figure S29. ^{1}H NMR of compound 4j

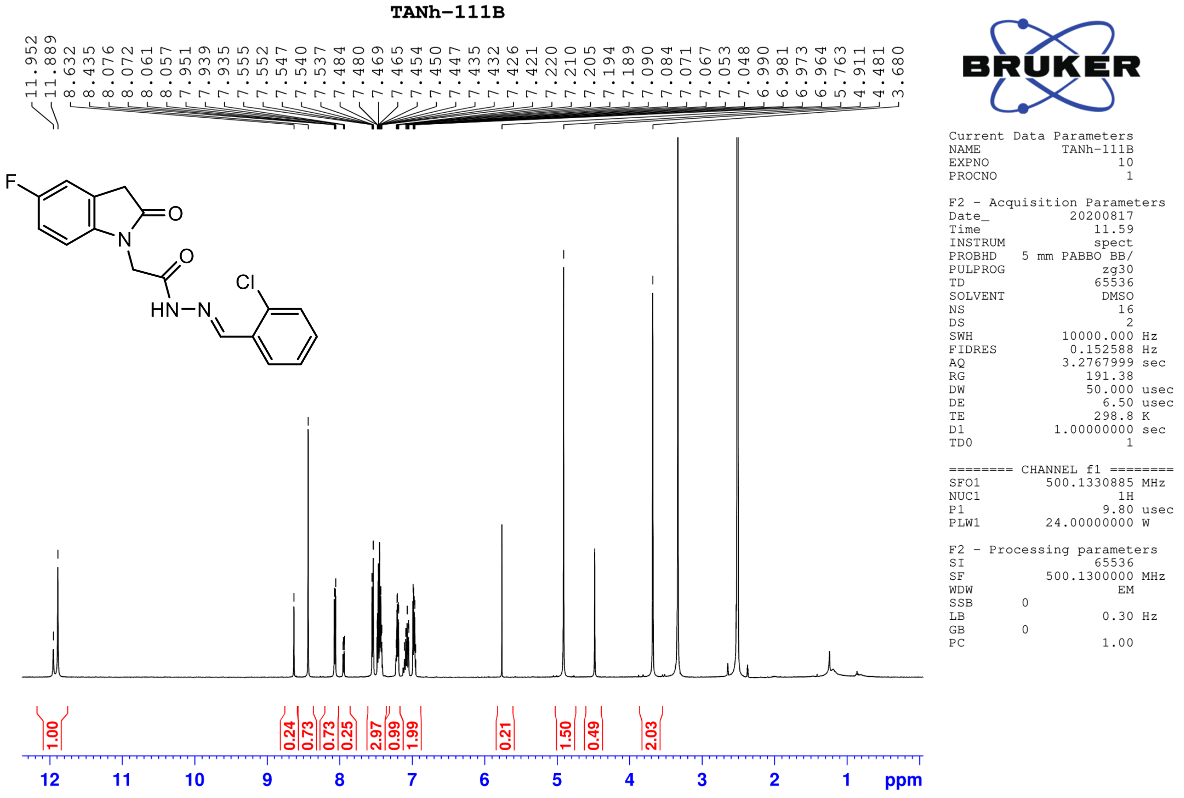

Figure S30. ^{13}C NMR of compound 4j

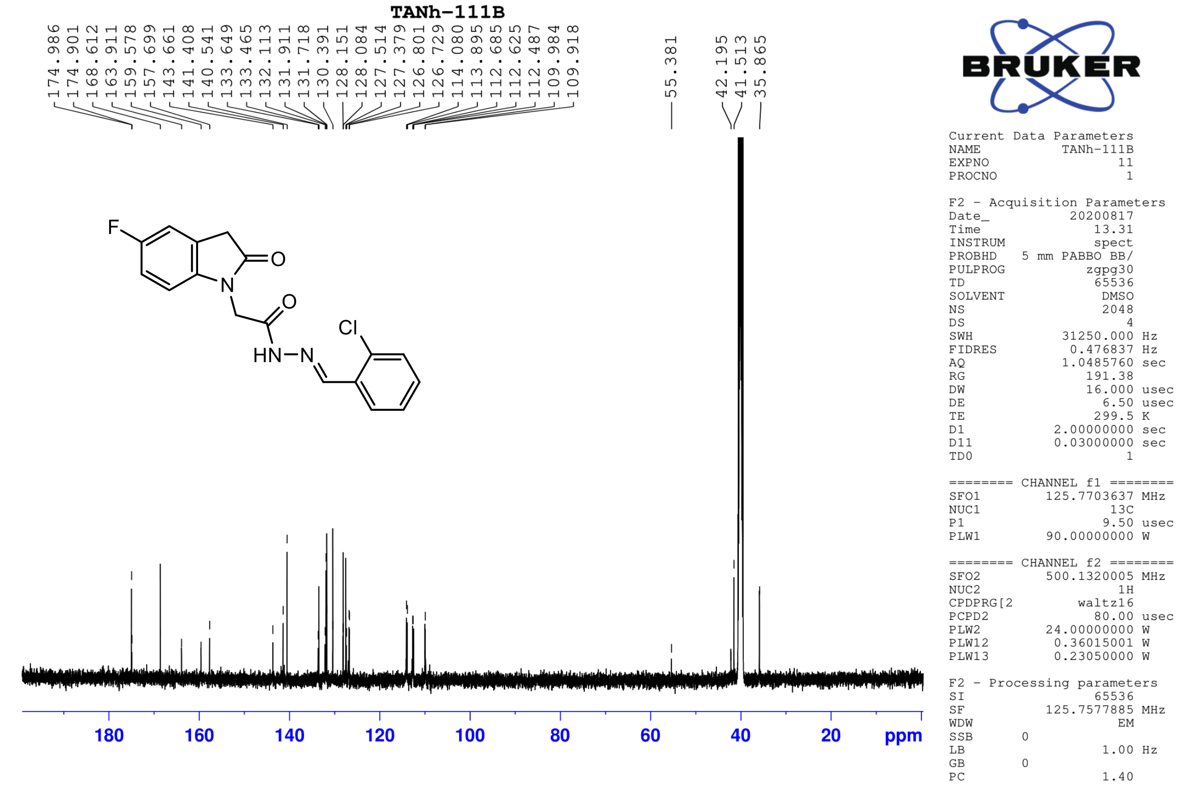

Figure S31. HRMS of compound 4k

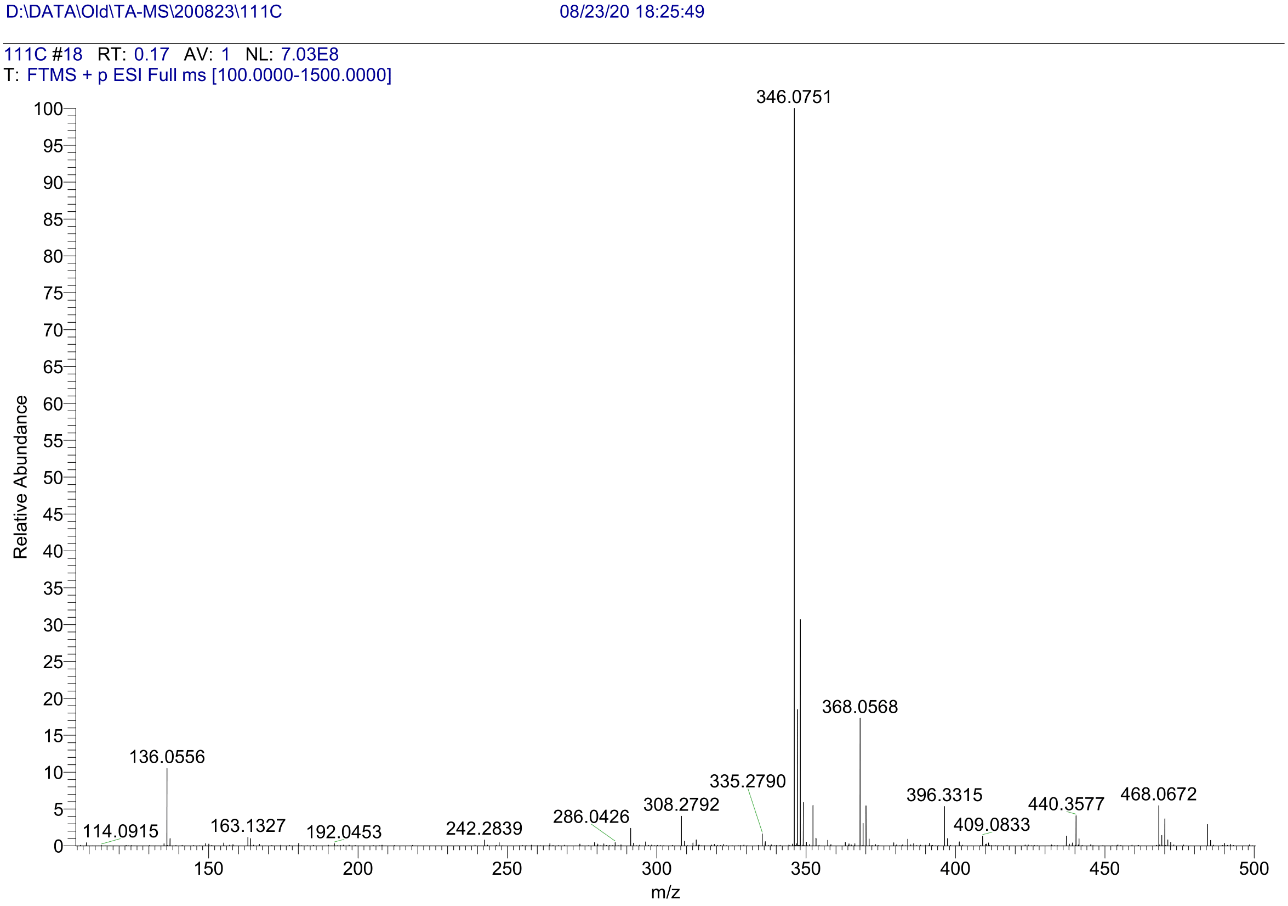

Figure S32. ^{1}H NMR of compound 4k

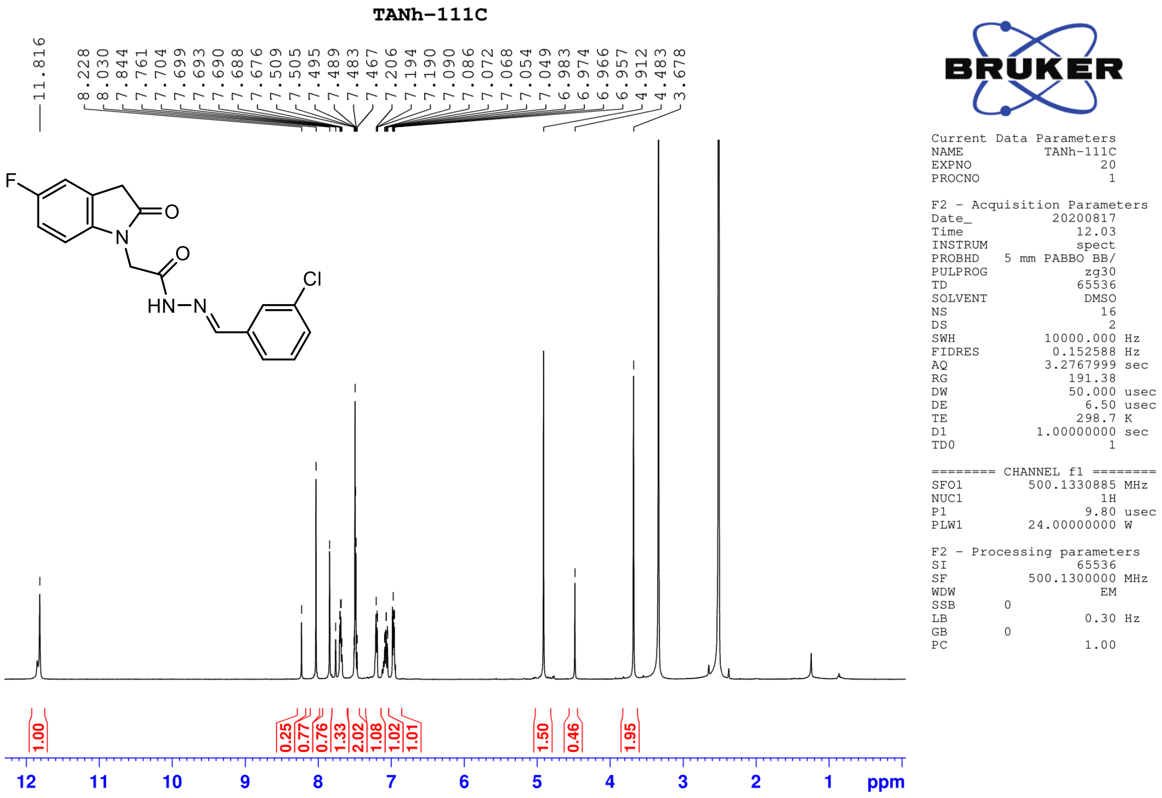

Figure S33. ^{13}C NMR of compound 4k

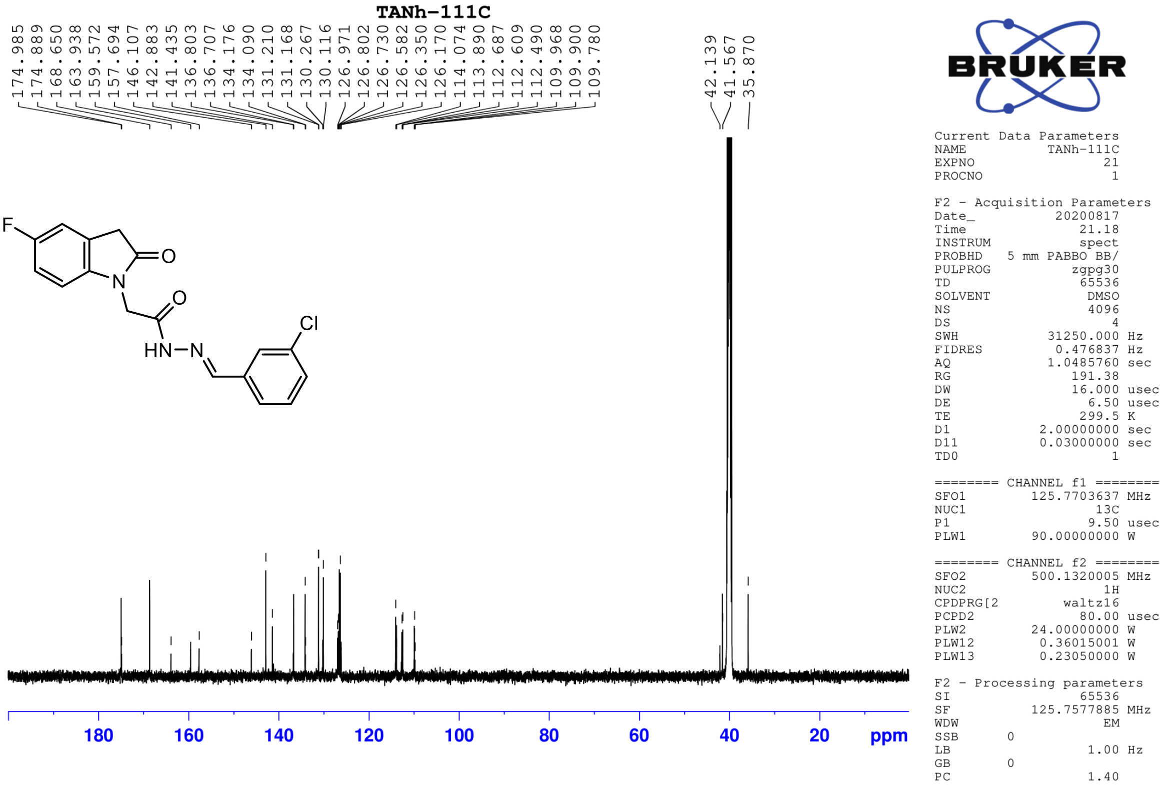

Figure S34. HRMS of compound 4l

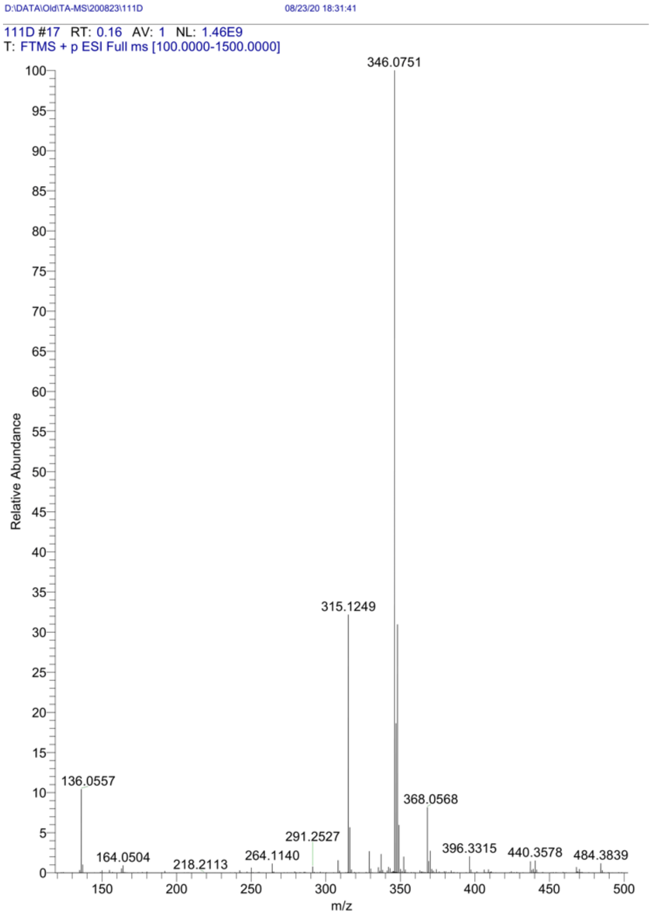

Figure S35. ^{1}**H NMR of compound 4l**

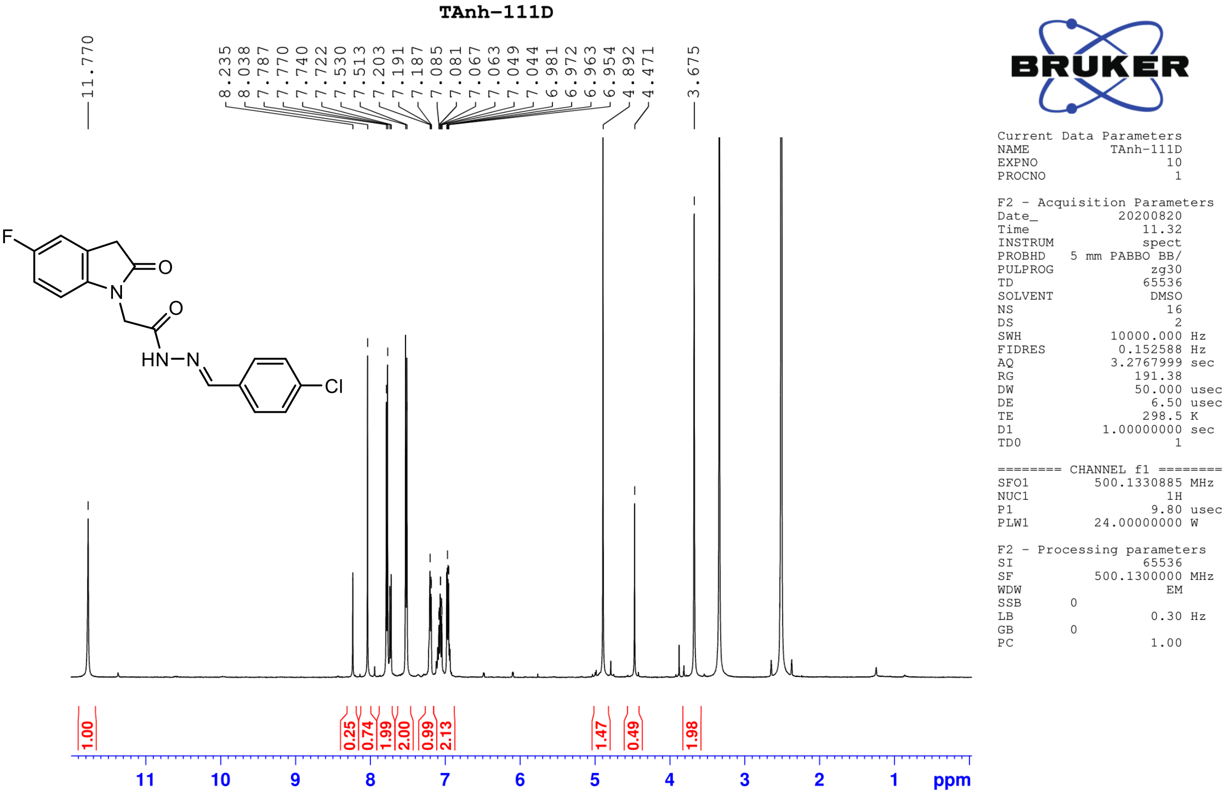

Figure S36. ^{13}**C NMR of compound 4l**

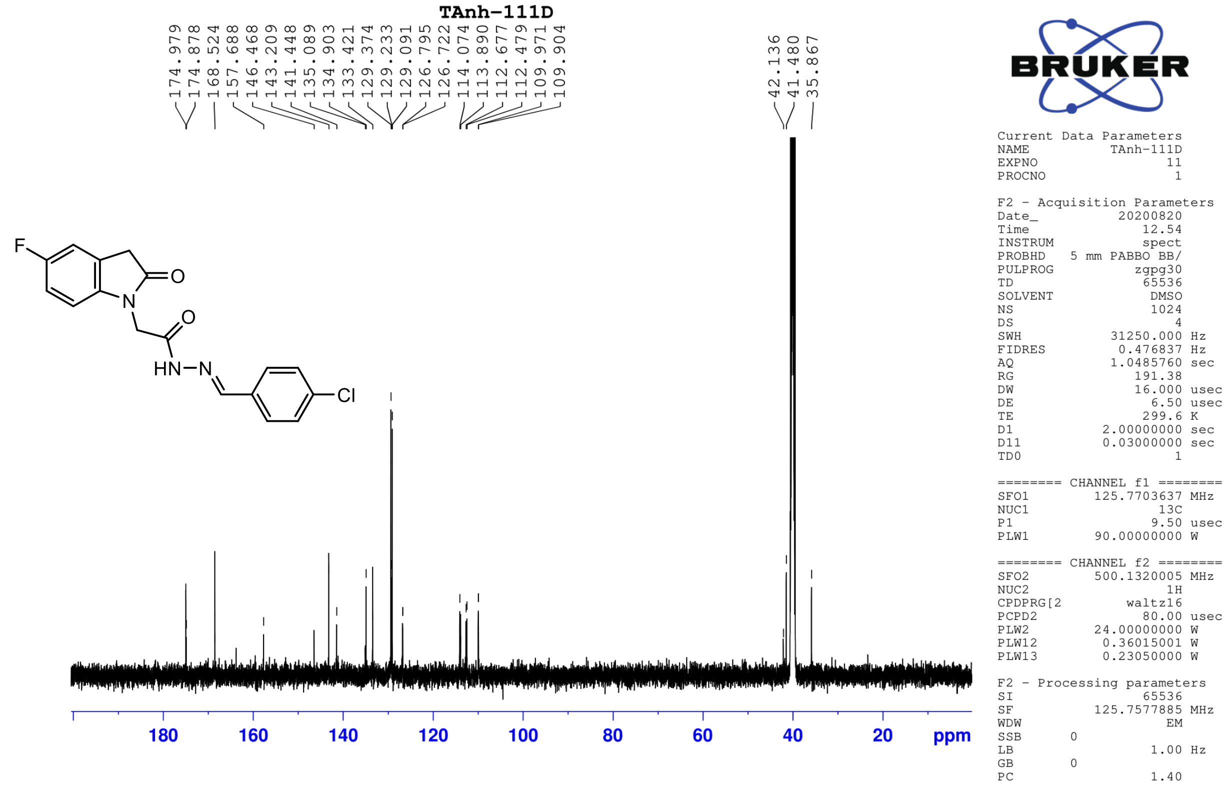

Figure S37. HRMS of compound 4m

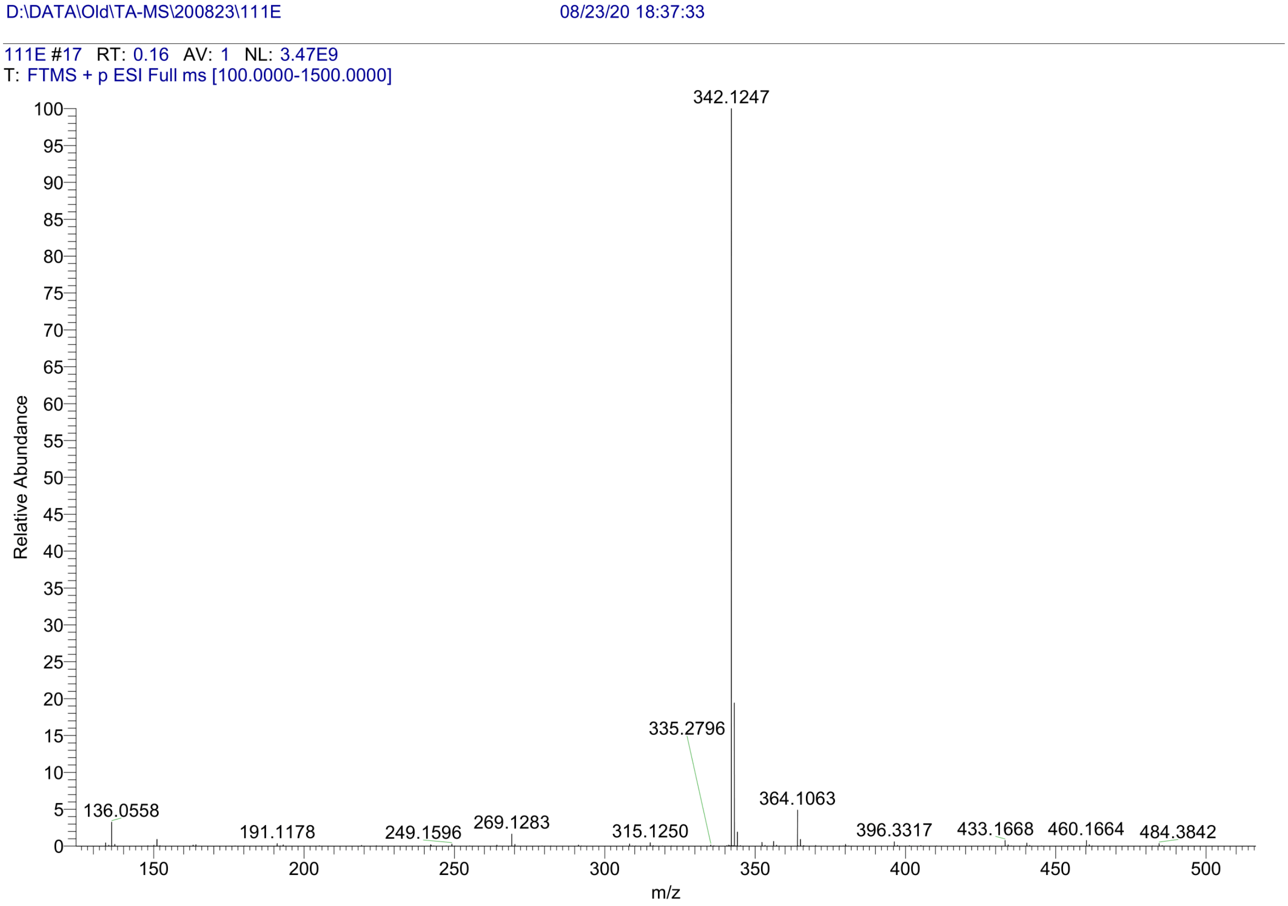

Figure S38. ^{1}H NMR of compound 4m

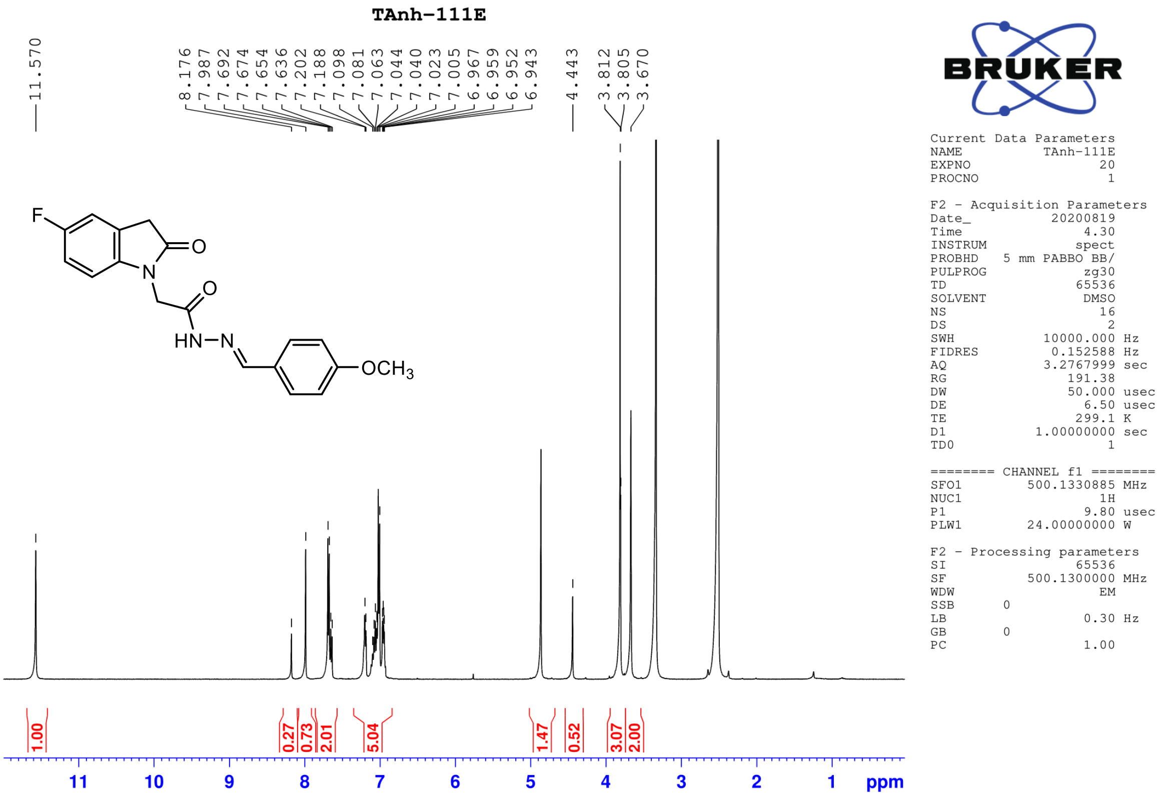

Figure S39. ^{13}C NMR of compound 4m

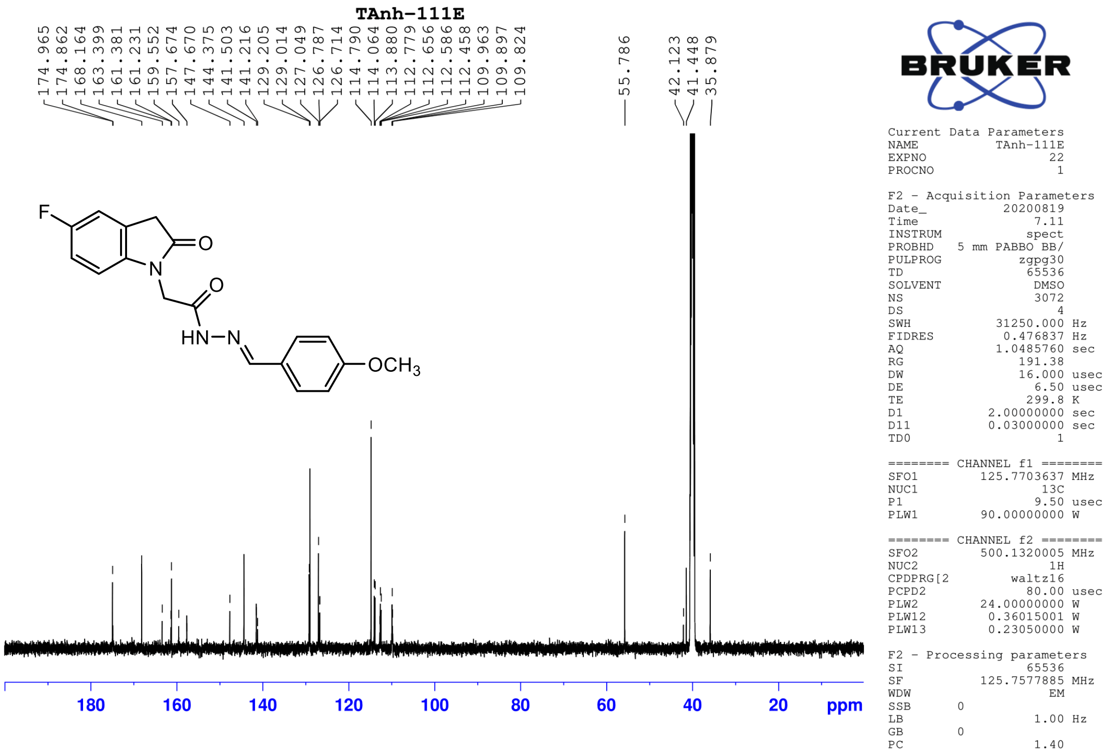

Figure S40. HRMS of compound 4n

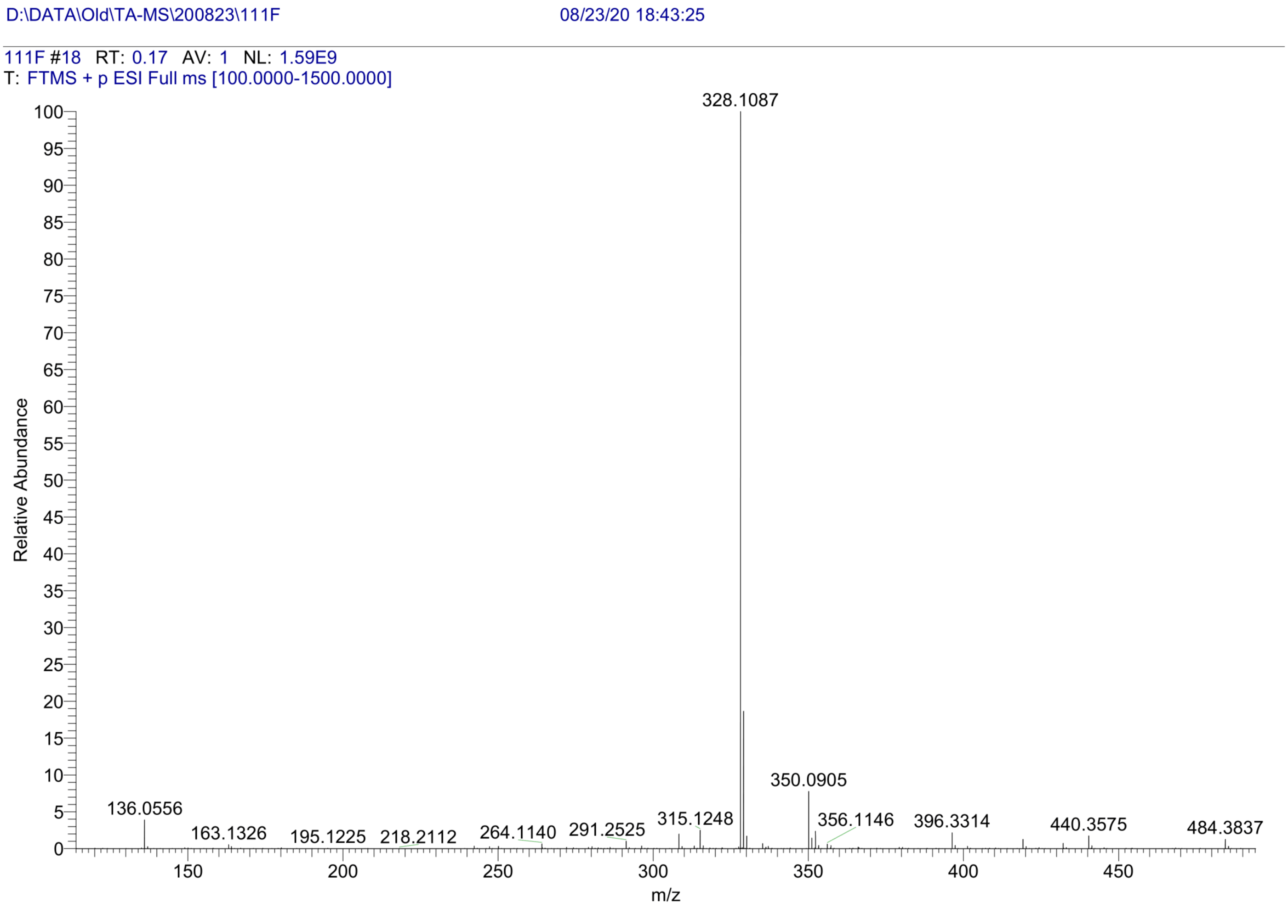

Figure S41. ^{1}H NMR of compound 4n

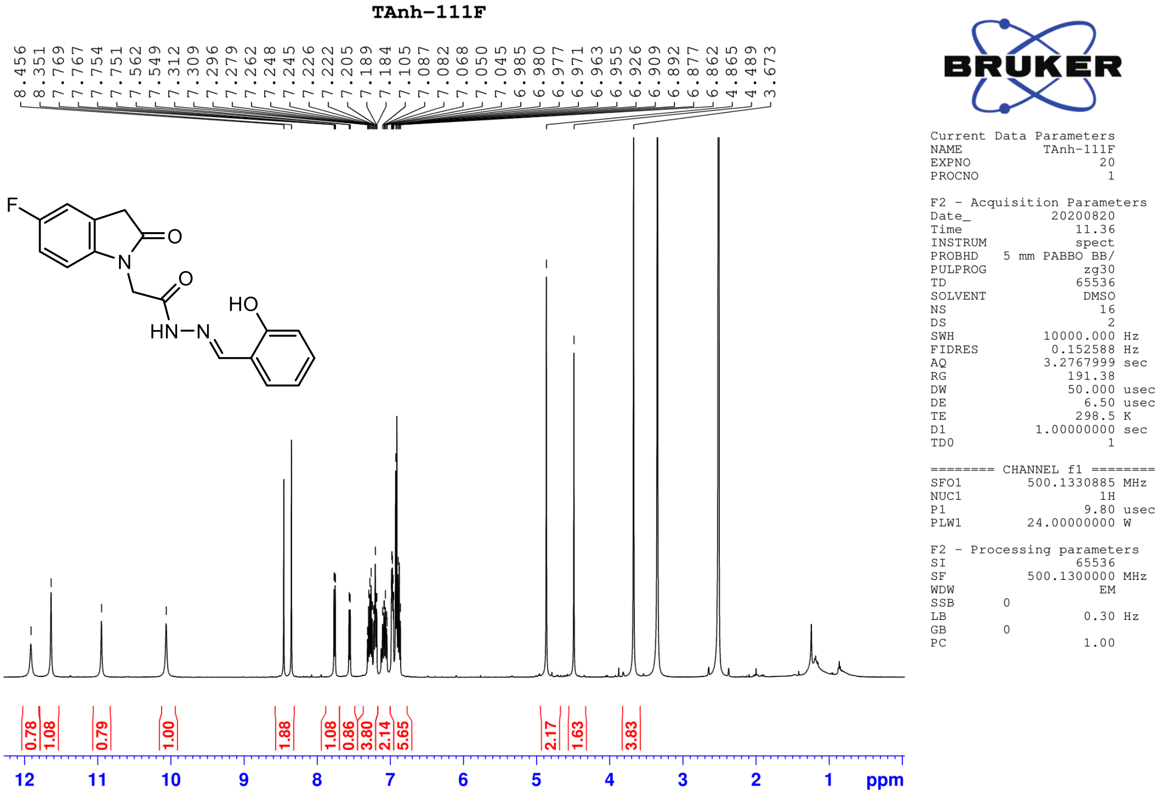

Figure S42. ^{13}C NMR of compound 4n

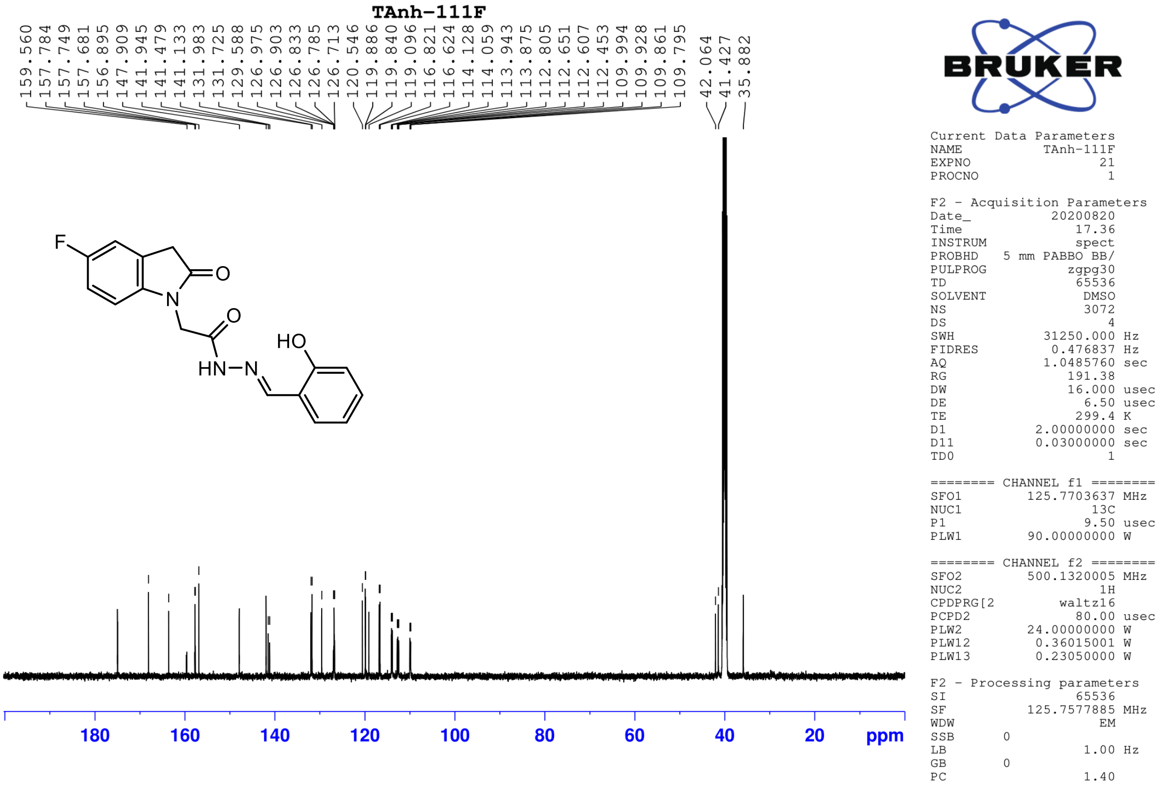

Figure S43. HRMS of compound 4o

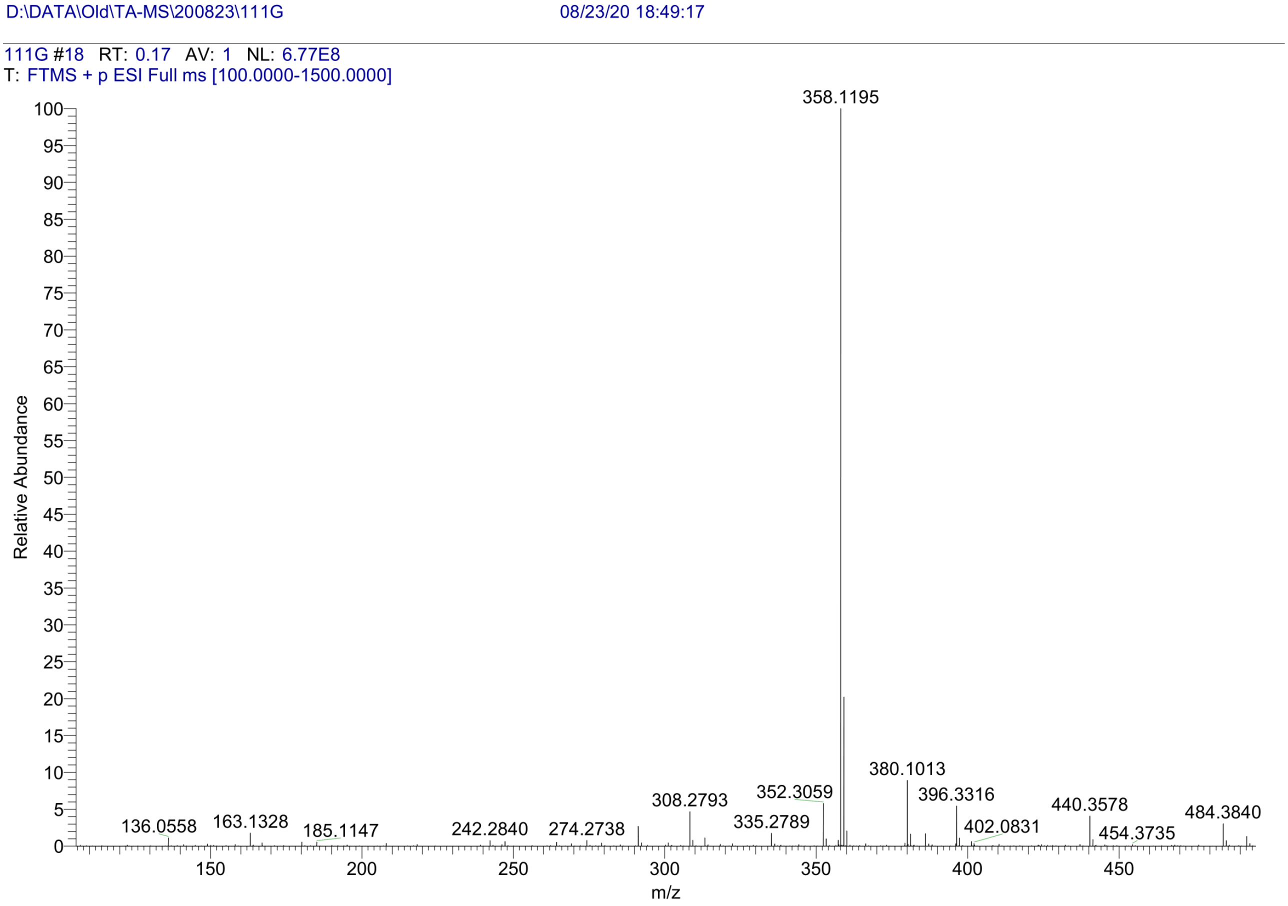

Figure S44. ^{1}H NMR of compound 4o

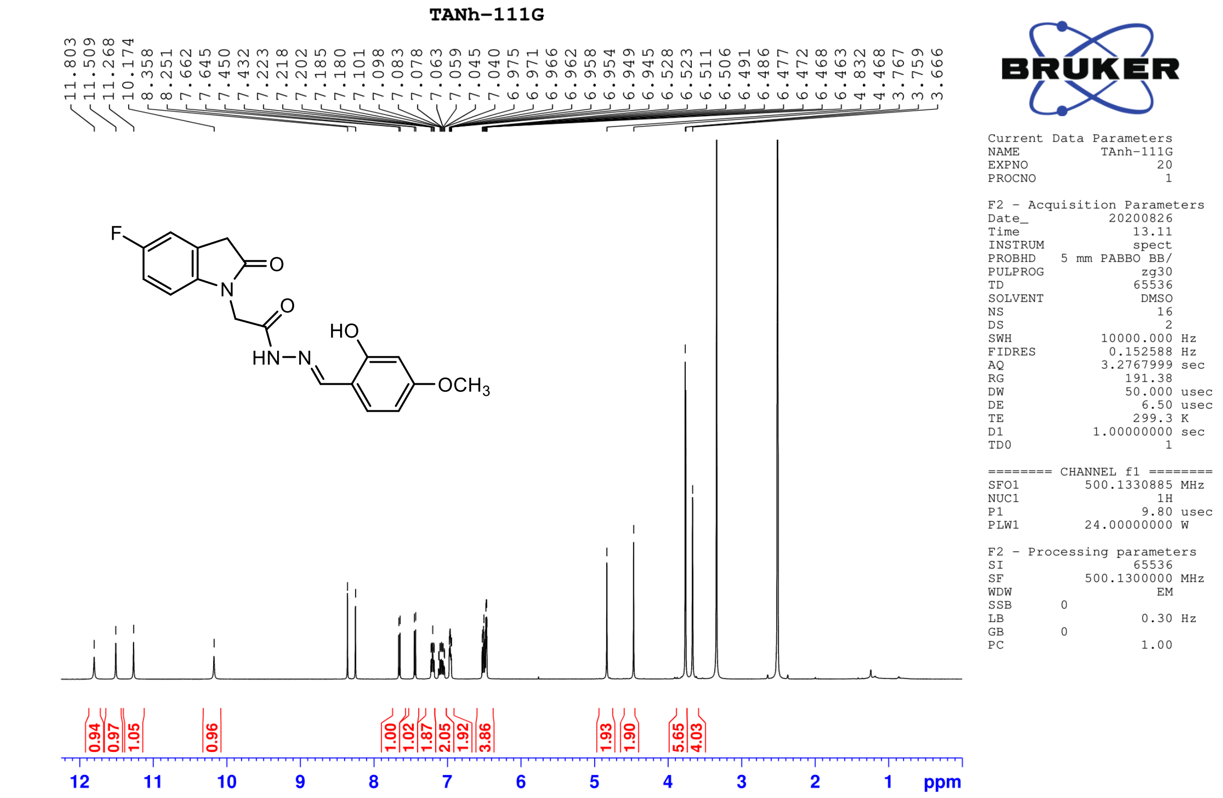

Figure S45. ^{13}C NMR of compound 4o

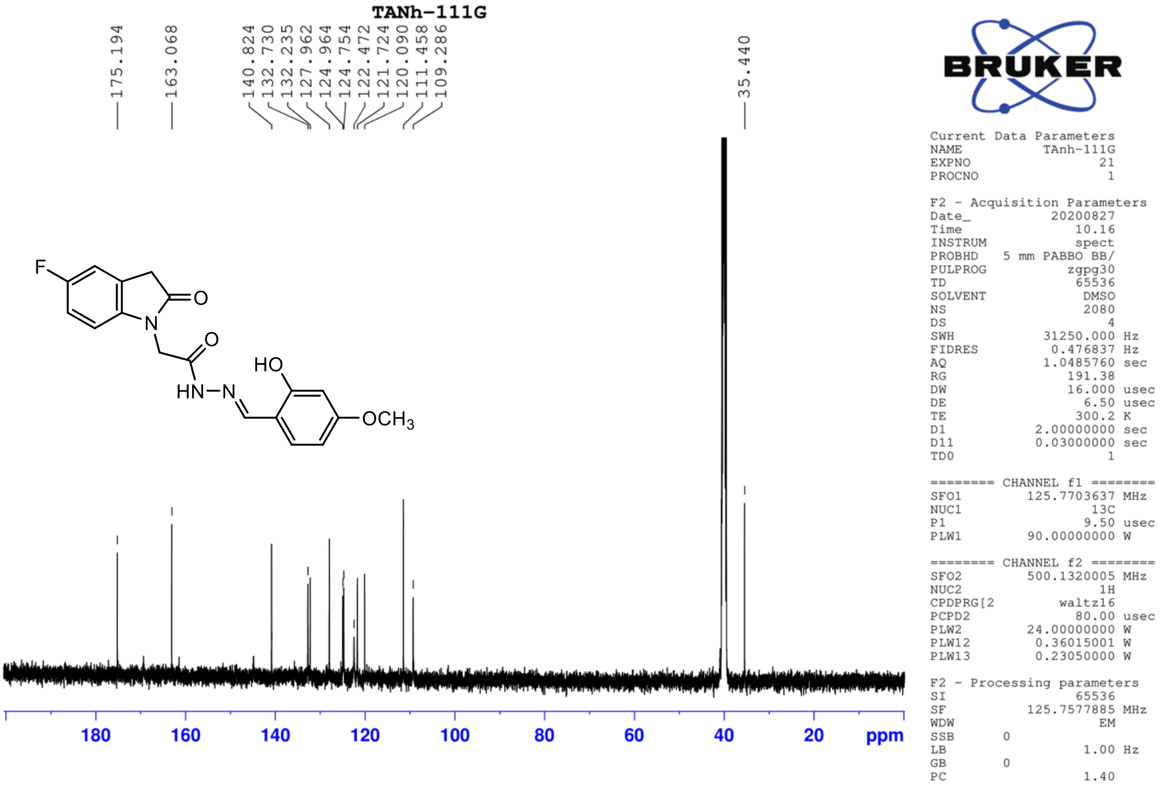

Figure S46. HRMS of compound 4p

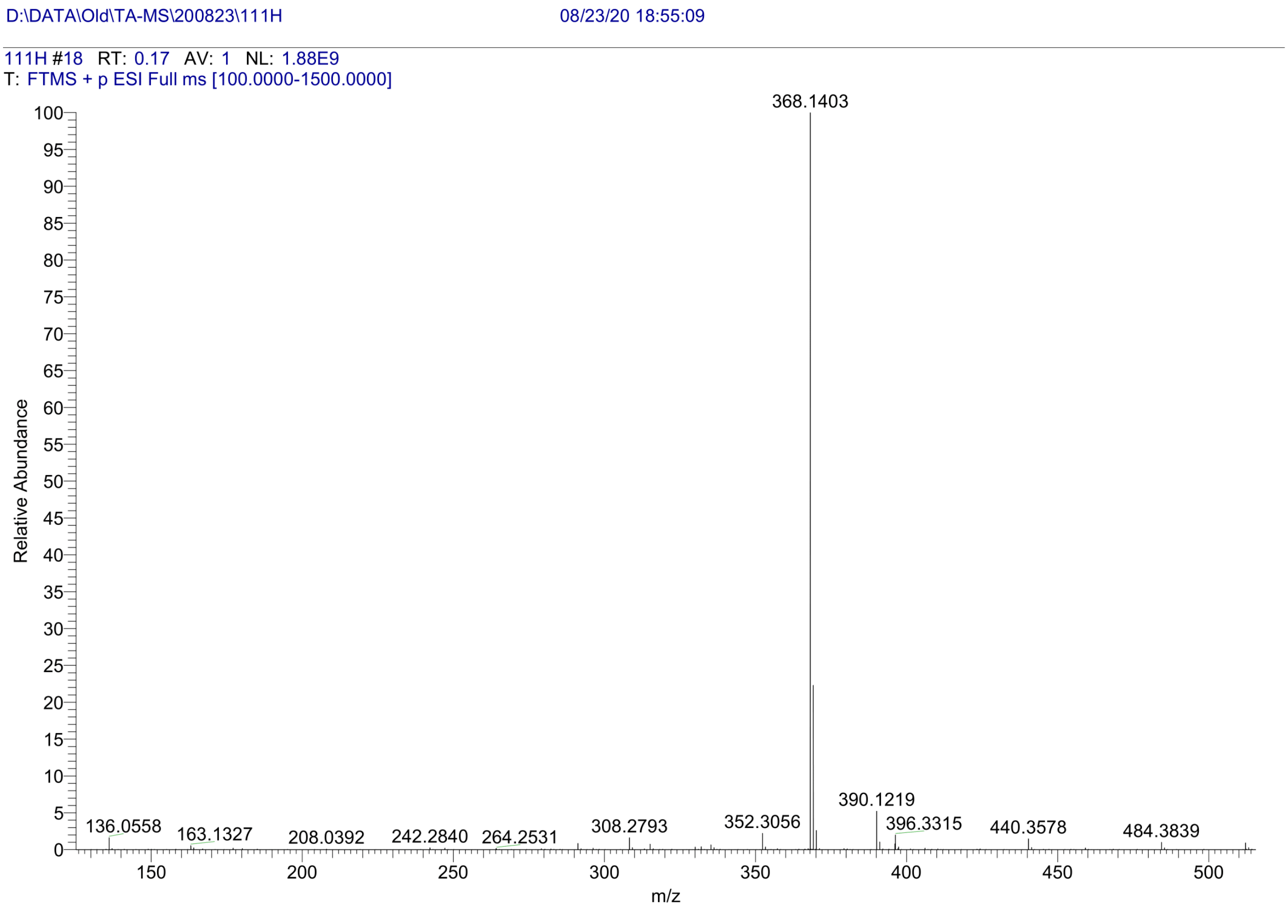

Figure S47. [1]**H NMR of compound 4p**

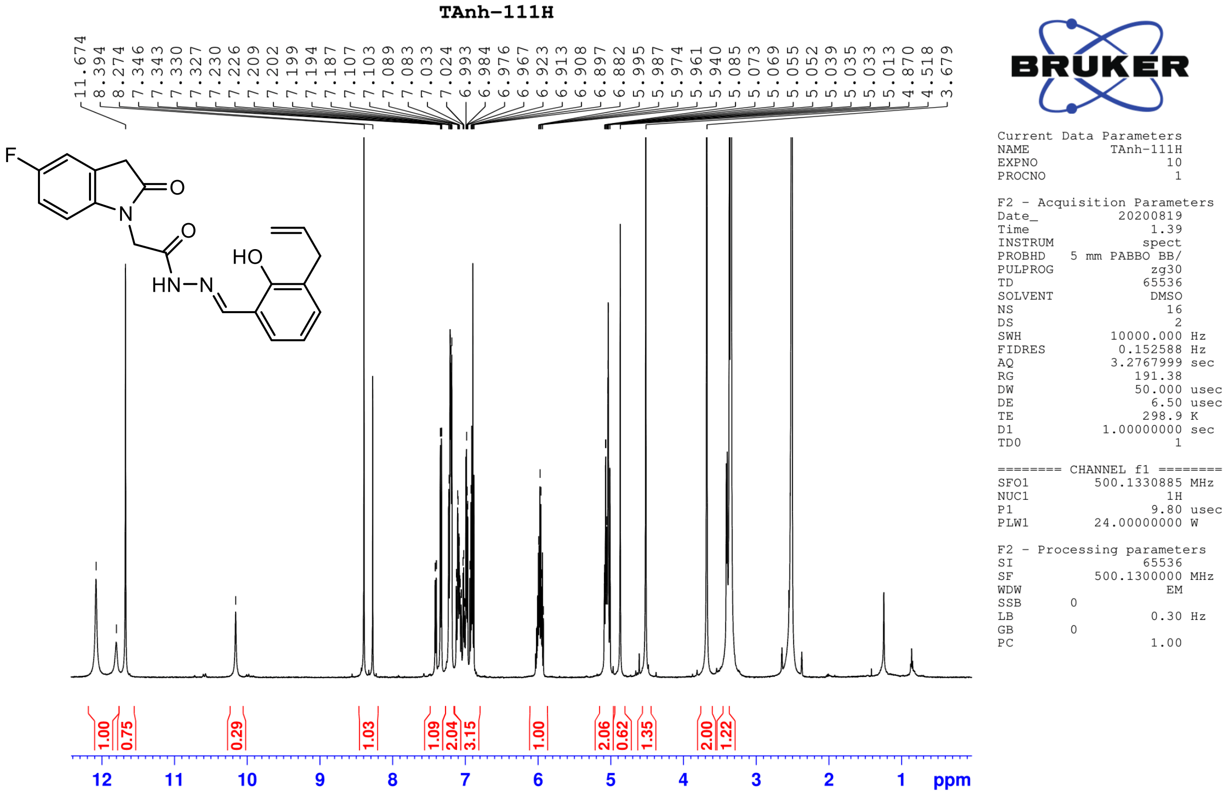

Figure S48. [13]**C NMR of compound 4p**

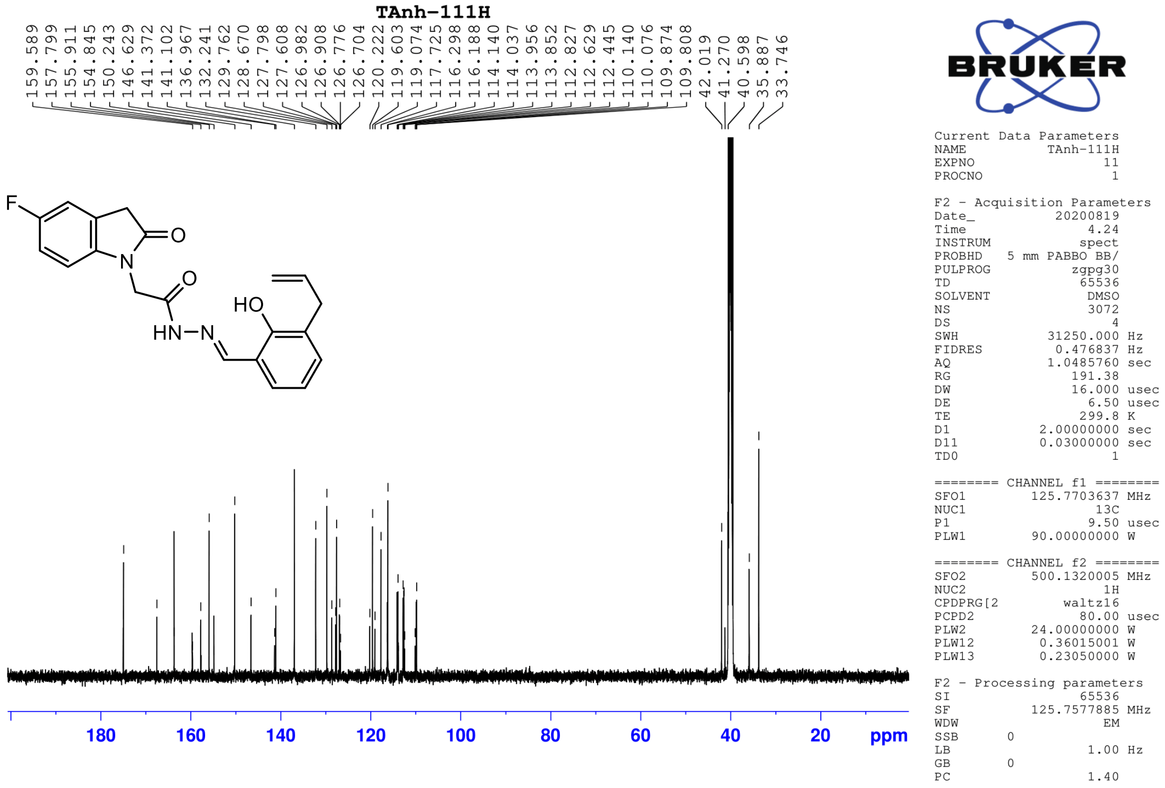

Figure S49. HRMS of compound 5a

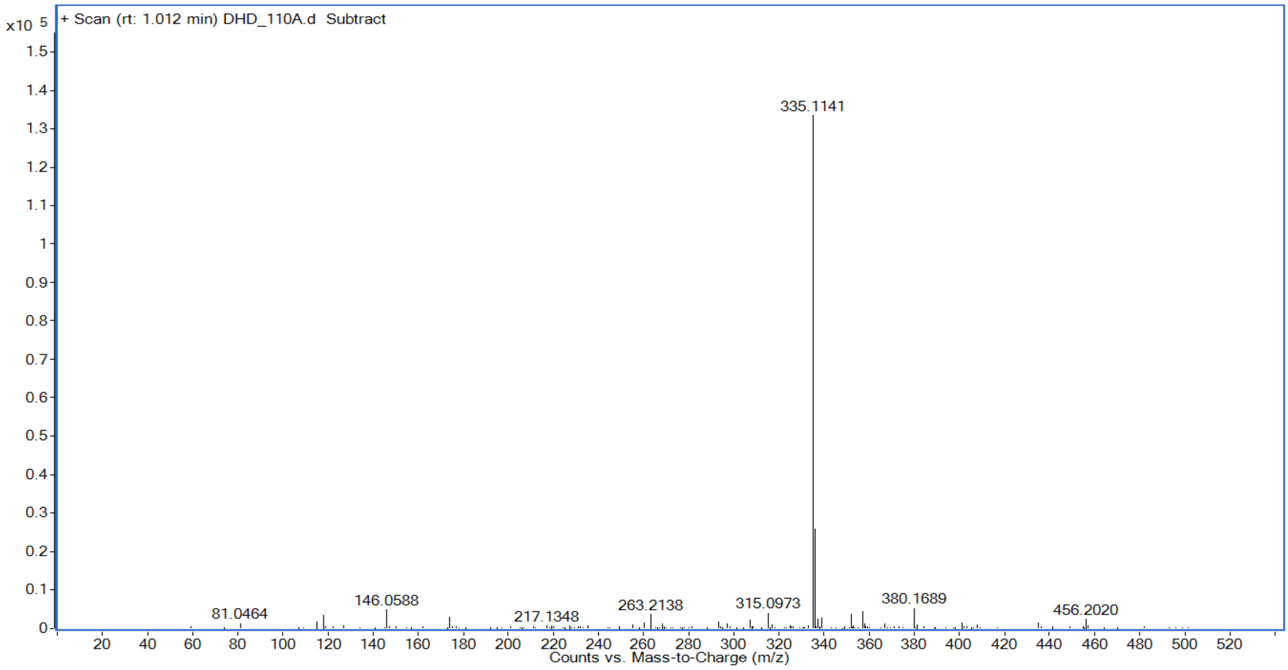

Figure S50. ^{1}H NMR of compound 5a

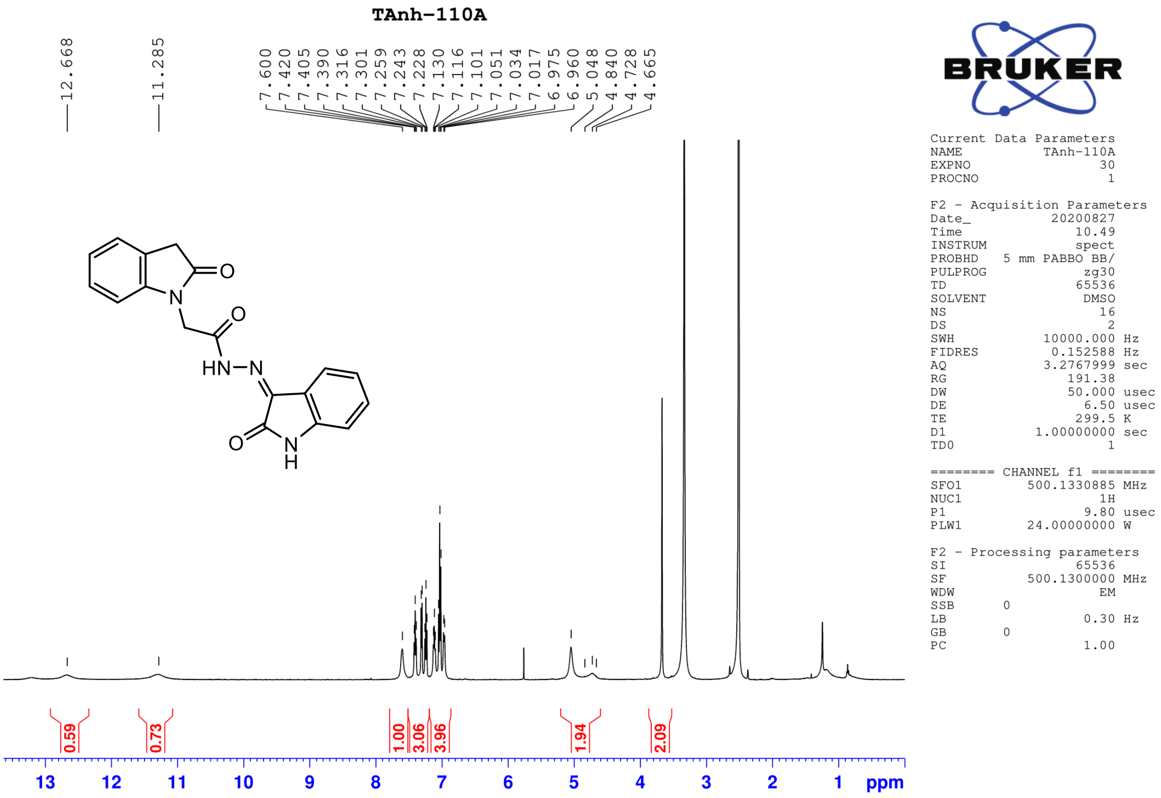

Figure S51. ^{13}C NMR of compound 5a

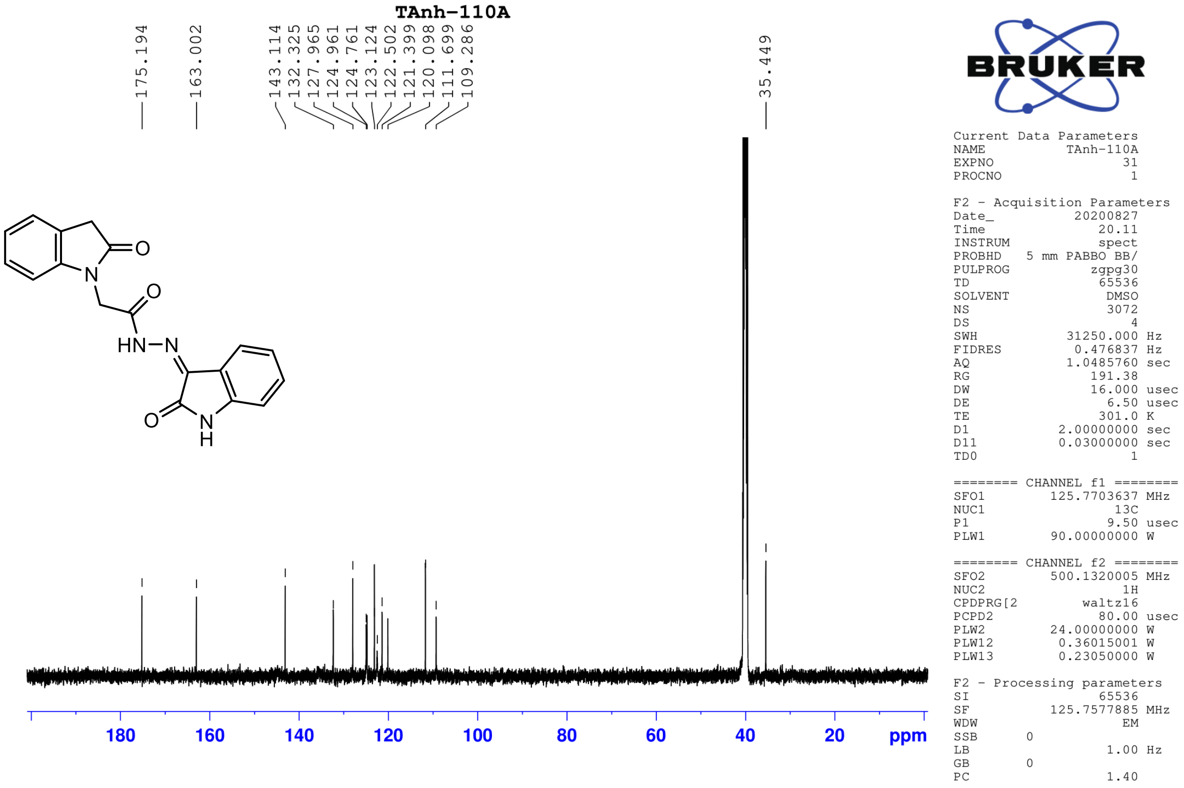

Figure S52. HRMS of compound 5b

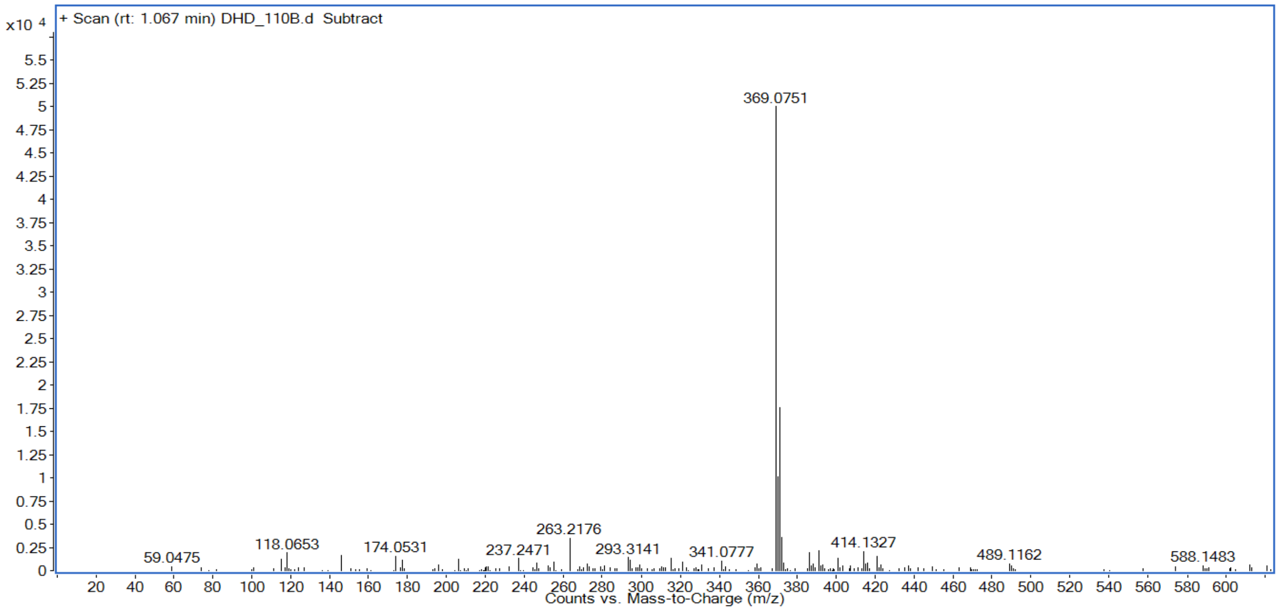

Figure S53. ^{1}H NMR of compound 5b

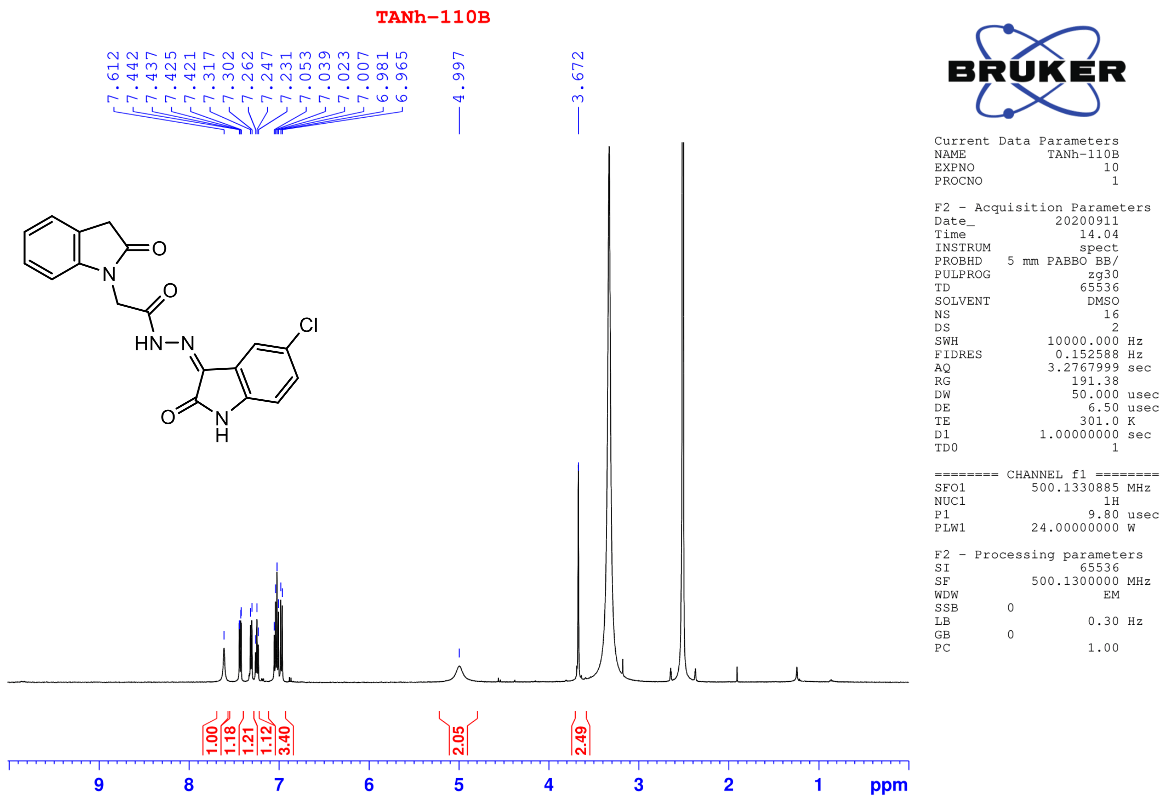

Figure S54. ^{13}C NMR of compound 5b

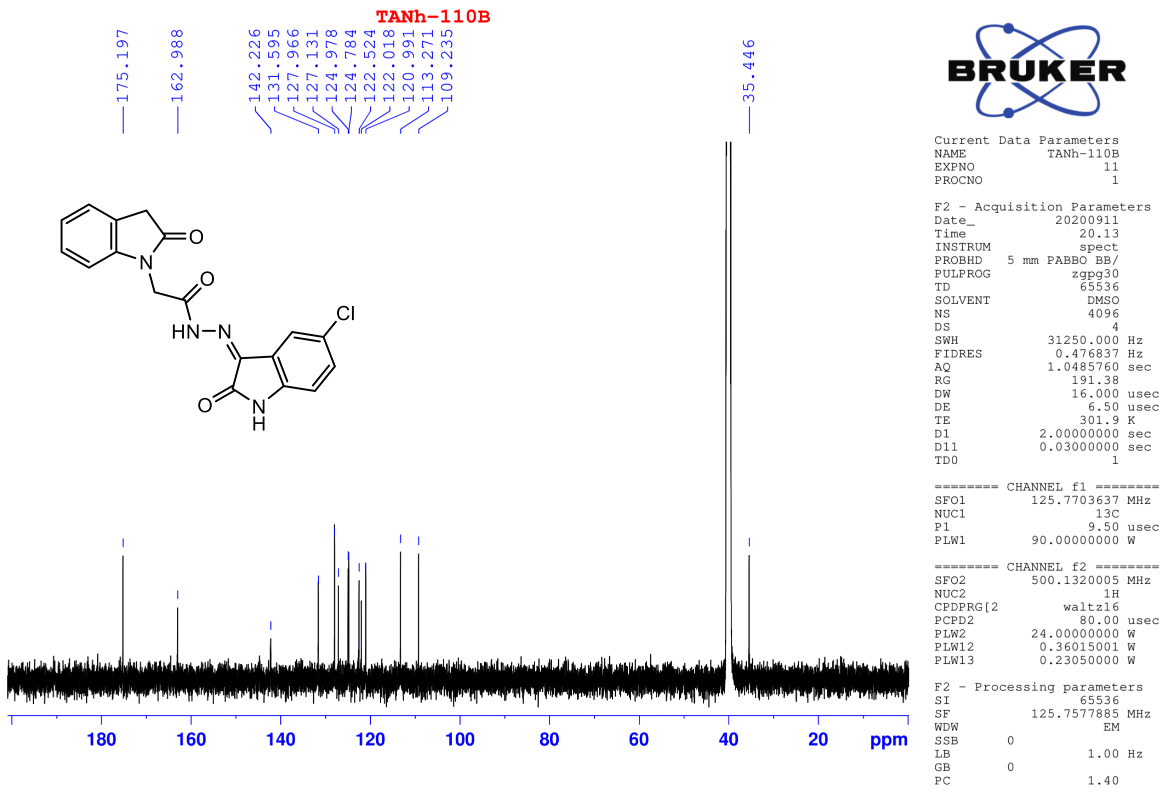

Figure S55. HRMS of compound 5c

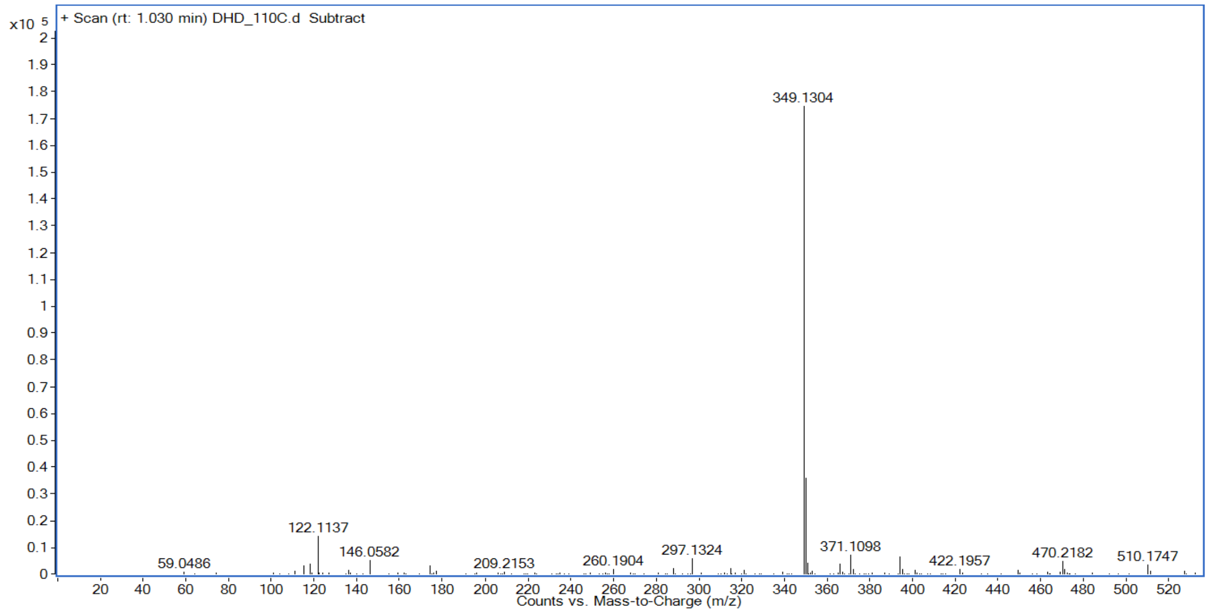

Figure S56. ^{1}H NMR of compound 5c

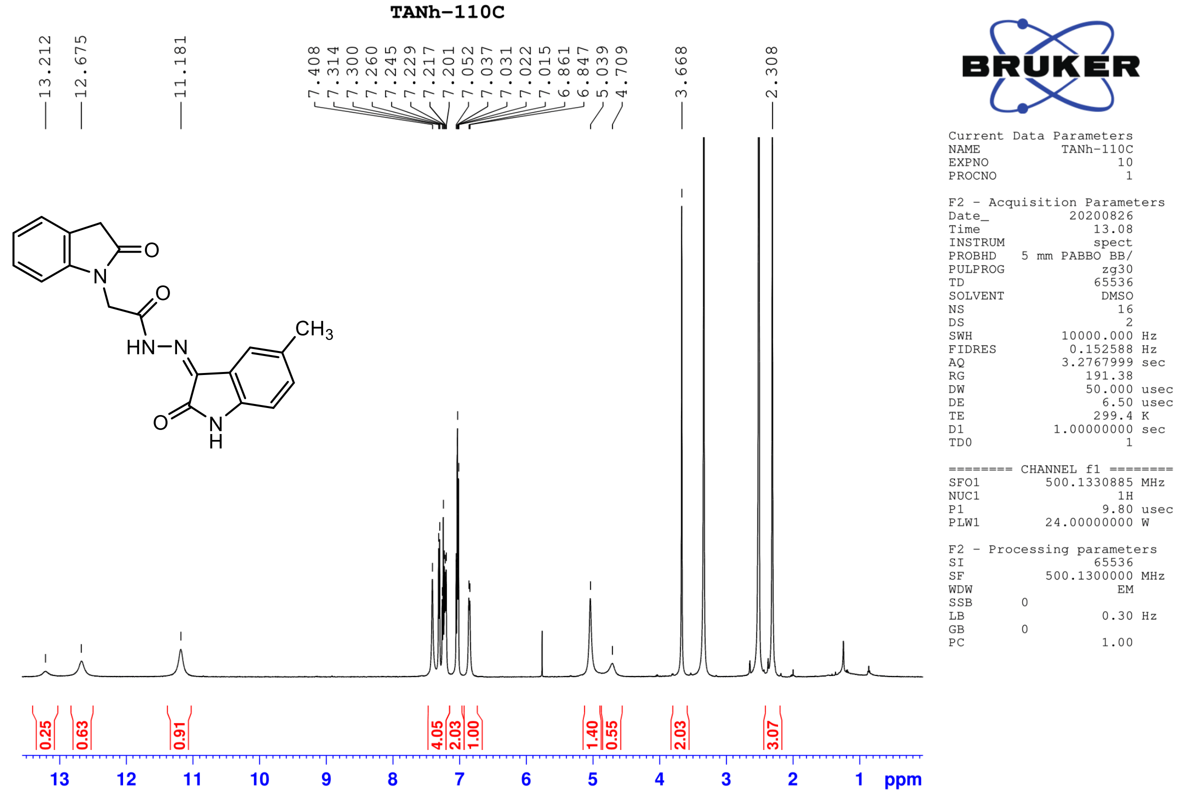

Figure S57. ^{13}C NMR of compound 5c

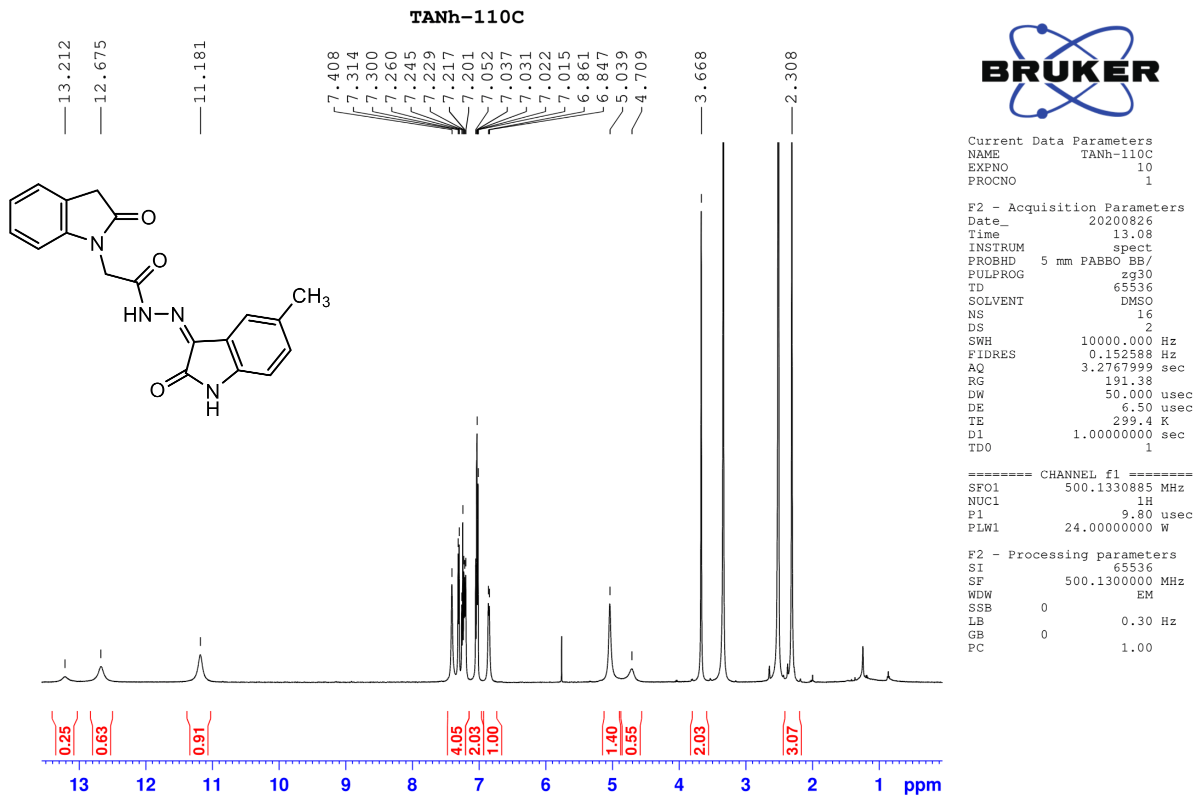

Figure S58. HRMS of compound 5d

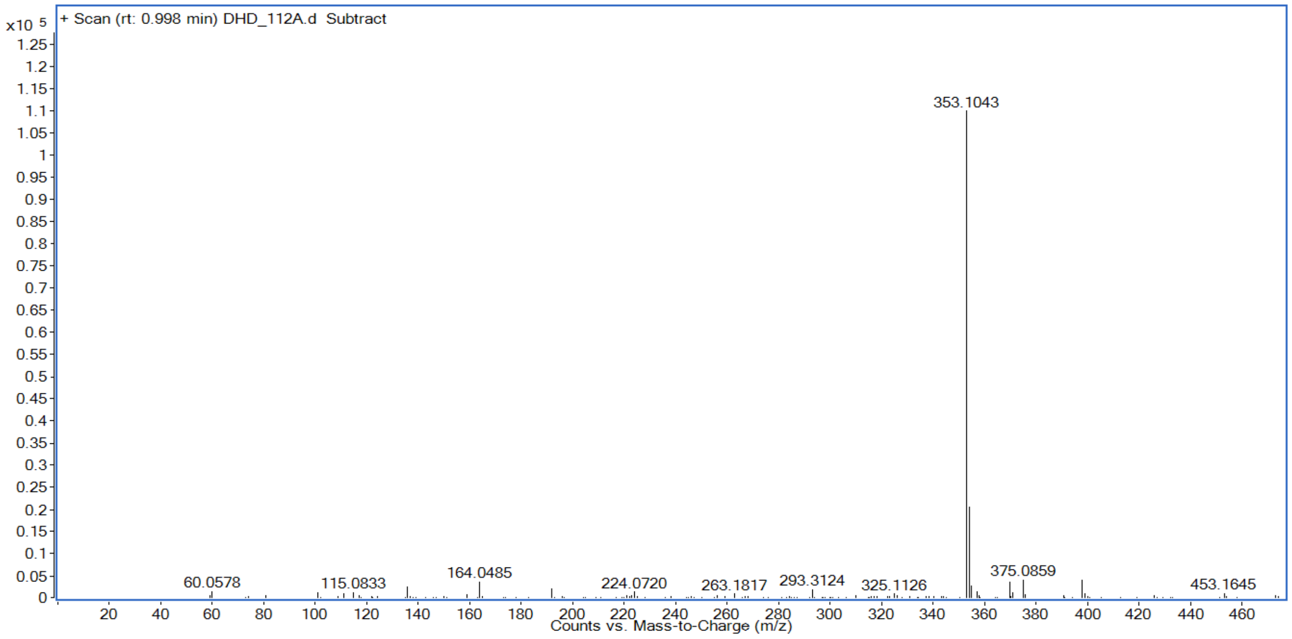

Figure S59. ^{1}H NMR of compound 5d

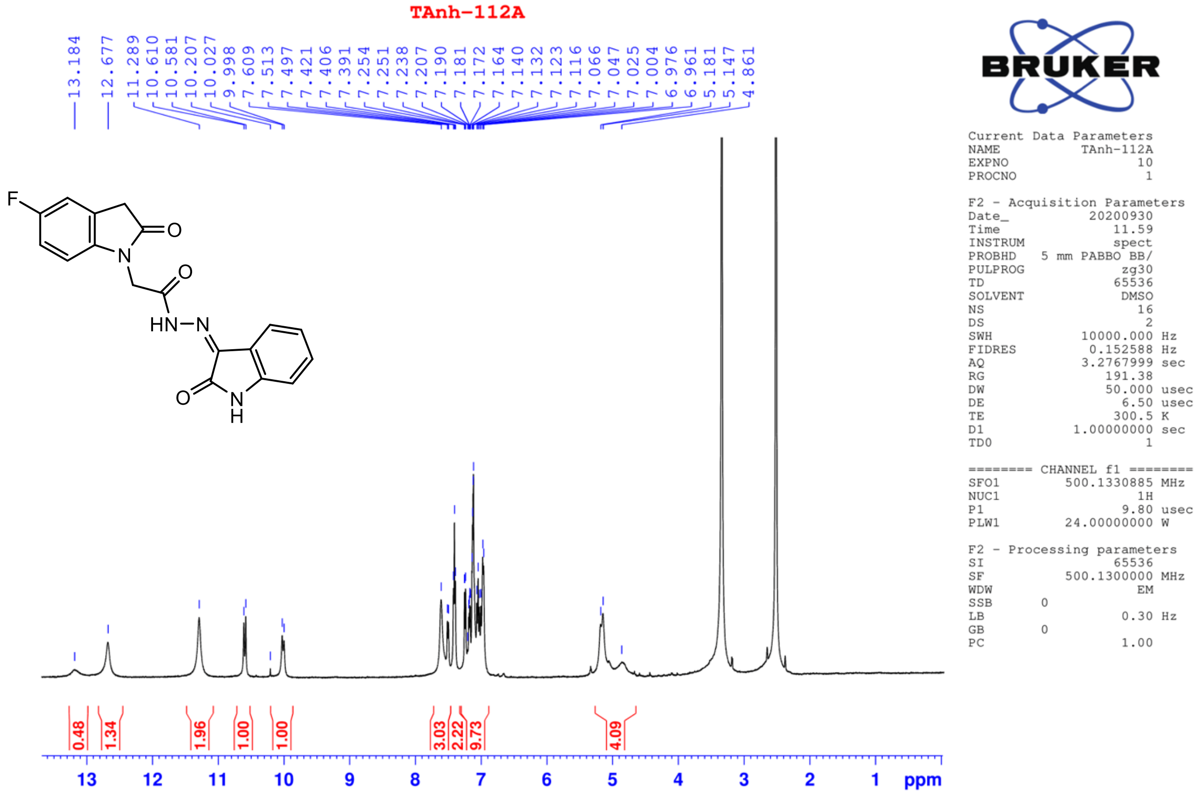

Figure S60. ^{13}C NMR of compound 5d

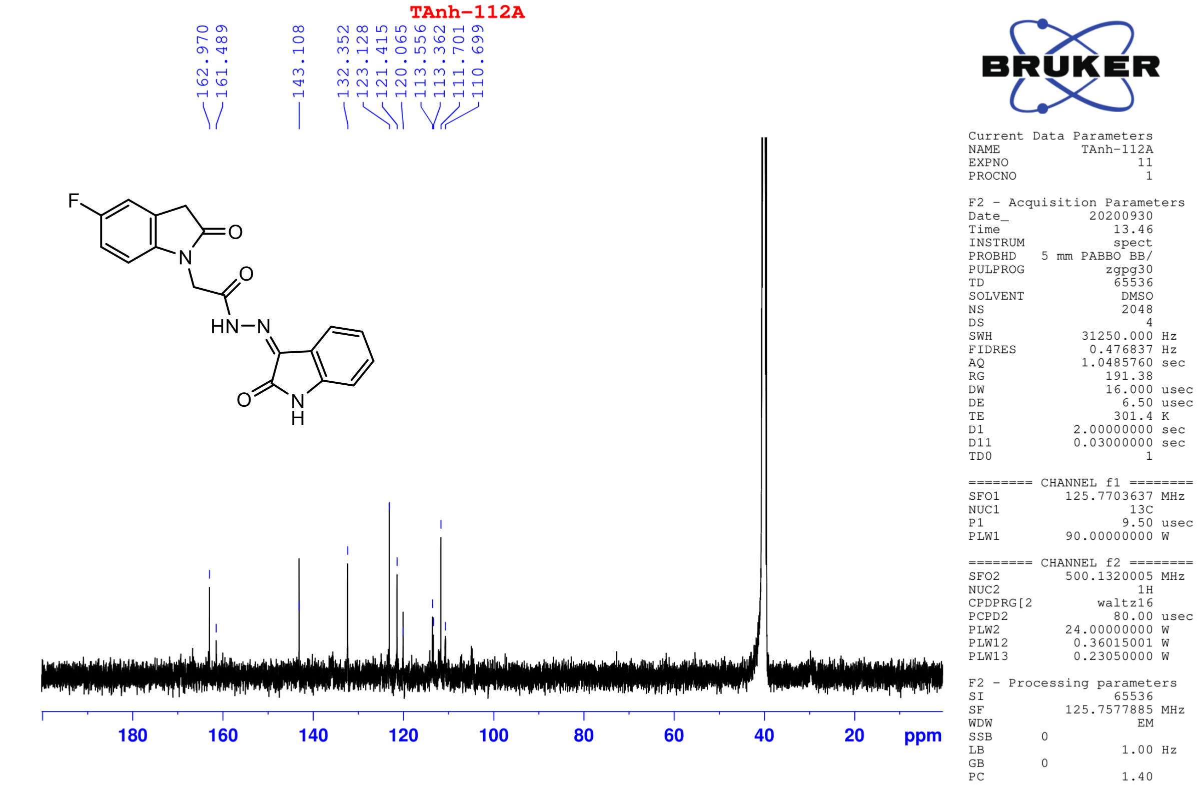

Figure S61. HRMS of compound 5e

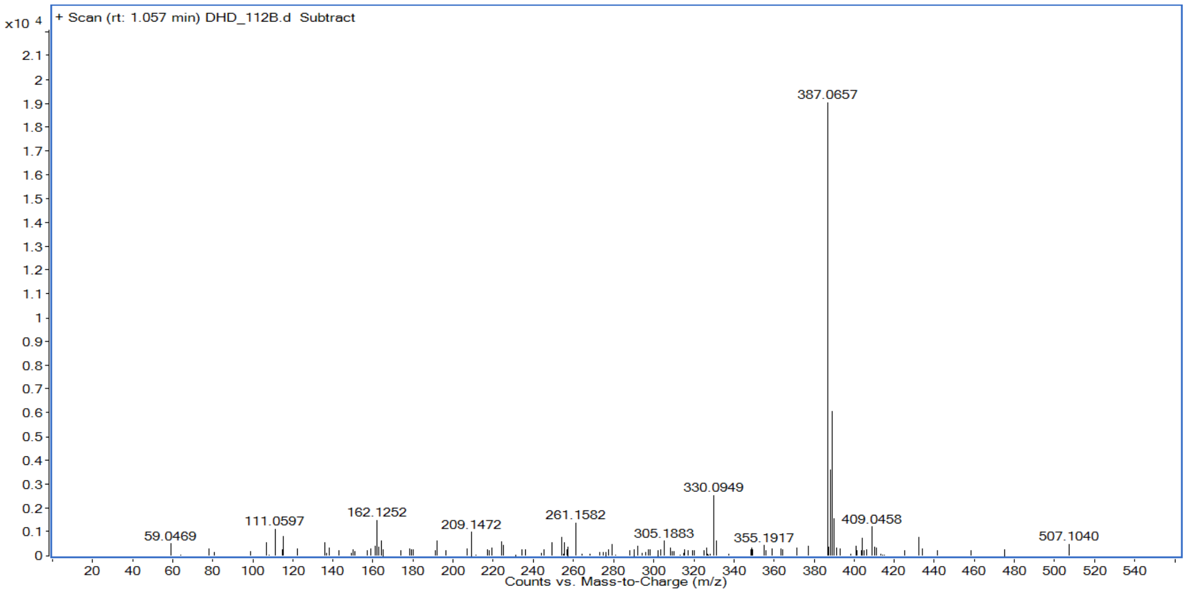

Figure S62. ^{1}H NMR of compound 5e

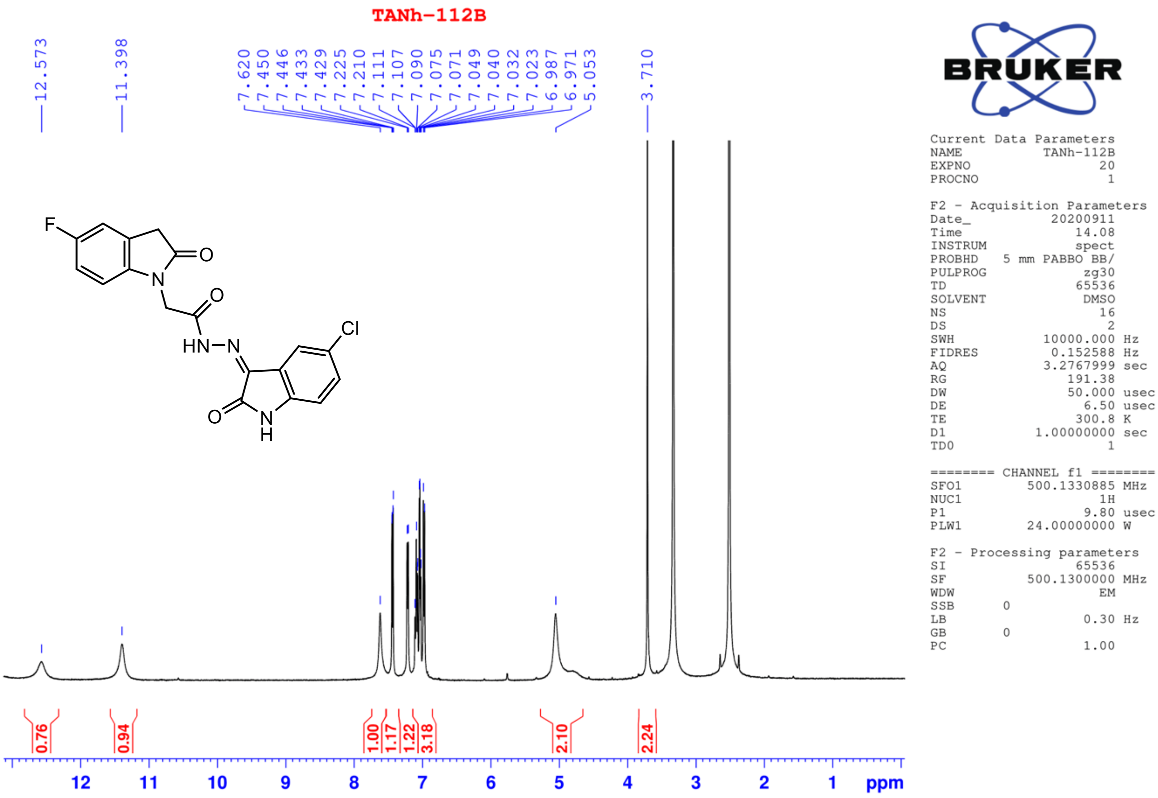

Figure S63. ^{13}C NMR of compound 5e

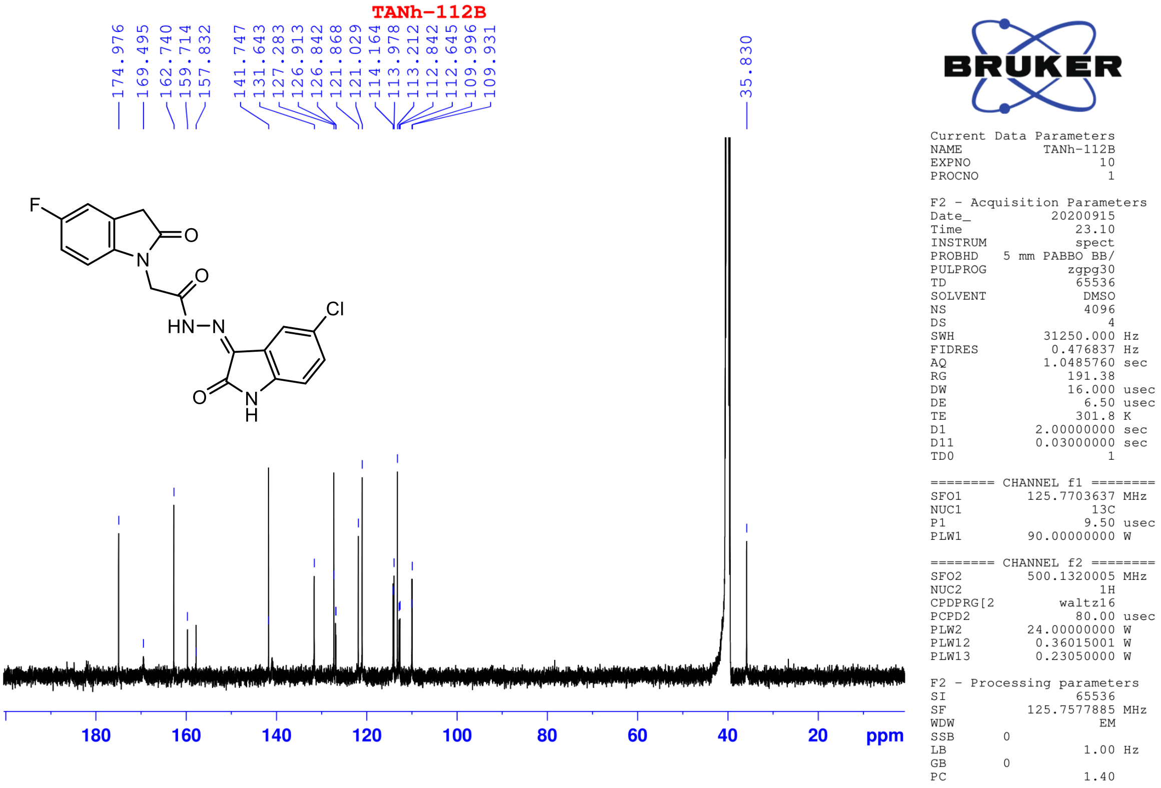

Figure S64. HRMS of compound 5f

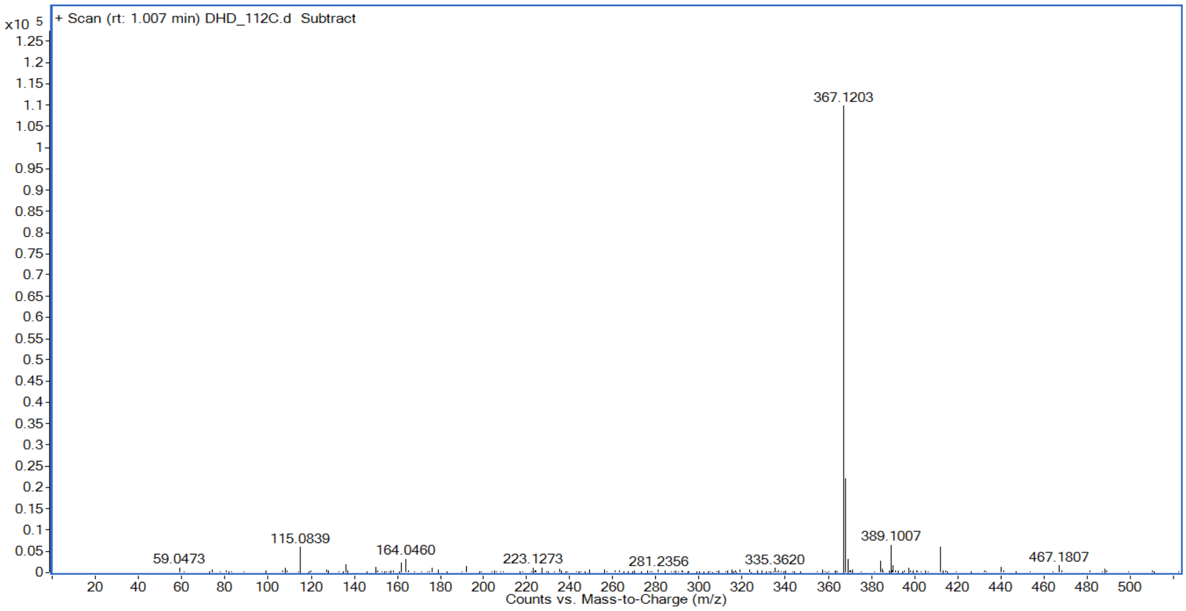

Figure S65. ^{1}H NMR of compound 5f

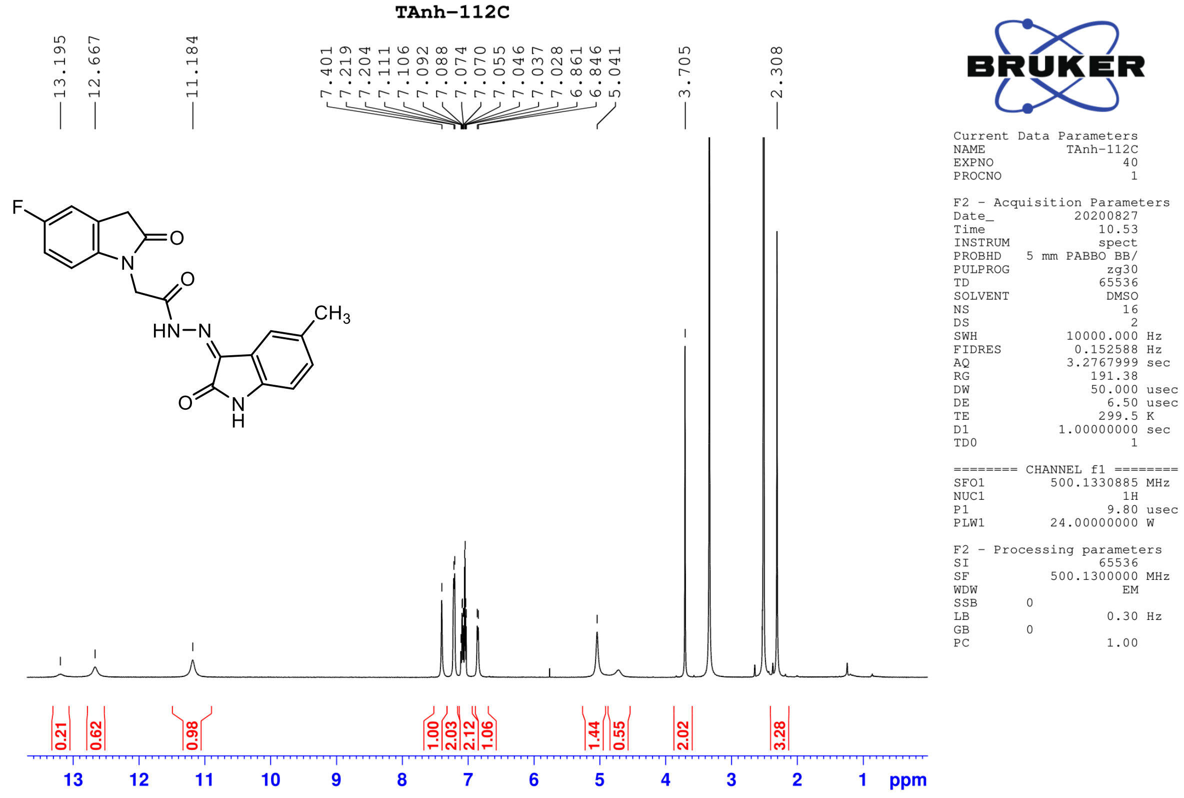

Figure S66. ^{13}C NMR of compound 5f

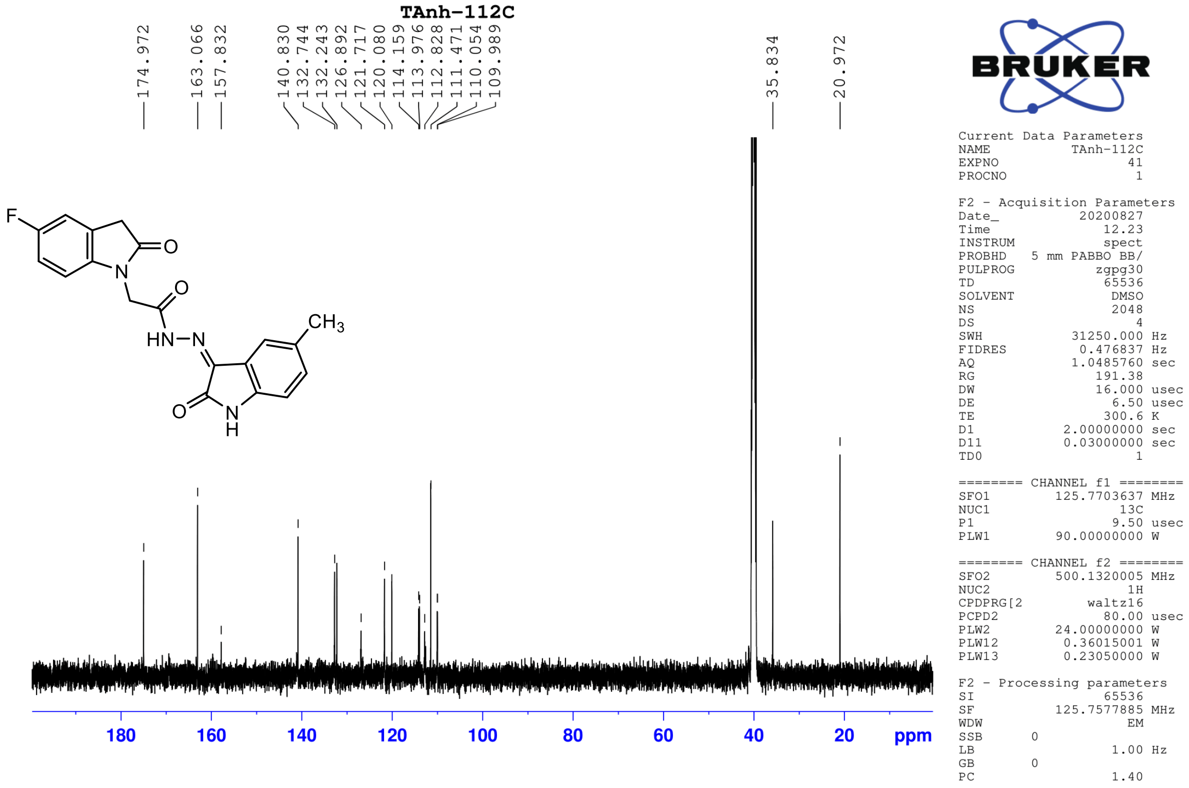

Supplement: Supplementary file 1 — Supplementary Figures. [file 41598_2022_6887_MOESM1_ESM.pdf]
